# Scavenging of Alkylperoxyl Radicals by Addition to Ascorbate: An Alternative Mechanism to Electron Transfer

**DOI:** 10.3390/antiox13101194

**Published:** 2024-10-01

**Authors:** Gabriel Robert, J. Richard Wagner

**Affiliations:** 1 Department of Biochemistry and Functional Genomics, Faculty of Medicine and Health Sciences, Université de Sherbrooke, 3001, 12e Avenue Nord, Sherbrooke, QC J1H 5N4, Canada; gabriel.robert@usherbrooke.ca; 2 Department of Medical Imaging and Radiation Sciences, Faculty of Medicine and Health Sciences, Université de Sherbrooke, 3001, 12e Avenue Nord, Sherbrooke, QC J1H 5N4, Canada

**Keywords:** antioxidant, peroxyl radicals, alkoxyl radicals, reactive oxygen species, DNA damage

## Abstract

Vitamin C (ascorbate; Asc) is a biologically important antioxidant that scavenges reactive oxygen species such as deleterious alkylperoxyl radicals (ROO^•^), which are generated by radical-mediated oxidation of biomolecules in the presence of oxygen. The radical trapping proprieties of Asc are conventionally attributed to its ability to undergo single-electron transfers with reactive species. According to this mechanism, the reaction between Asc and ROO^•^ results in the formation of dehydroascorbate (DHA) and the corresponding hydroperoxides (ROOH). When studying the reactivity of DNA 5-(2′-deoxyuridinyl)methylperoxyl radicals, we discovered a novel pathway of ROO^•^ scavenging by Asc. The purpose of this study is to elucidate the underlying mechanism of this reaction with emphasis on the characterization of intermediate and final decomposition products. We show that the trapping of ROO^•^ by Asc leads to the formation of an alcohol (ROH) together with an unstable cyclic oxalyl-l-threonate intermediate (cOxa-Thr), which readily undergoes hydrolysis into a series of open-chain oxalyl-l-threonic acid regioisomers. The structure of products was determined by detailed MS and NMR analyses. The above transformation can be explained by initial peroxyl radical addition (PRA) onto the C2=C3 enediol portion of Asc. Following oxidation of the resulting adduct radical, the product subsequently undergoes Baeyer-Villiger rearrangement, which releases ROH and generates the ring expansion product cOxa-Thr. The present investigation provides robust clarifications of the peroxide-mediated oxidation chemistry of Asc and DHA that has largely been obscured in the past by interference with autooxidation reactions and difficulties in analyzing and characterizing oxidation products. Scavenging of ROO^•^ by PRA onto Asc may have beneficial consequences since it directly converts ROO^•^ into ROH, which prevents the formation of potentially deleterious ROOH, although it induces the breakdown of Asc into fragments of oxalyl-l-threonic acid.

## 1. Introduction

Biologically relevant antioxidant molecules and enzymes play a major role in the protection of cellular components against the deleterious effects of oxidative stress. They operate on multiple levels to counteract damage by oxidants. Firstly, scavenging of primary free radicals and diffusible reactive species prevents or limits the initial generation of cellular lesions. Additionally, the reaction of small-molecule antioxidants with biomolecule-derived radical species can minimize damage and even, to a certain extent, chemically repair sites of attack by reducing carbon-centered radicals (R^•^) back to the corresponding alkyl groups (RH). However, oxygen competitively reacts with R^•^ at close to diffusion-controlled rates [1] to generate oxidizing alkylperoxyl radicals (ROO^•^) that can amplify damage further if not eliminated by radical-trapping antioxidants (RTA). In fact, the ability of ROO^•^ to undergo successive reactions with nearby macromolecules has the potential to produce two or more lesions in close proximity. This phenomenon has been typically associated with lipid peroxidation of membranes containing polyunsaturated fatty acids in which ROO^•^ undergoes a chain reaction, thereby leading to extensive oxidation of lipids from a single initiating radical [2]. The chain-breaking antioxidant α-tocopherol is essential for the inhibition of cellular lipid peroxidation, which has been shown to occur at a steady state rate of approximately 33 nM/h under basal cell culture conditions [3]. It is increasingly recognized that ROO^•^ can amplify radical-mediated damage to macromolecules, apart from the well-established case of polyunsaturated lipids [4,5,6,7]. The formation of peroxyl radical species in DNA and their subsequent reaction with neighboring constituents can lead to the modification of two vicinal nucleotides, referred to as tandem lesions [8].

**Figure 1:**
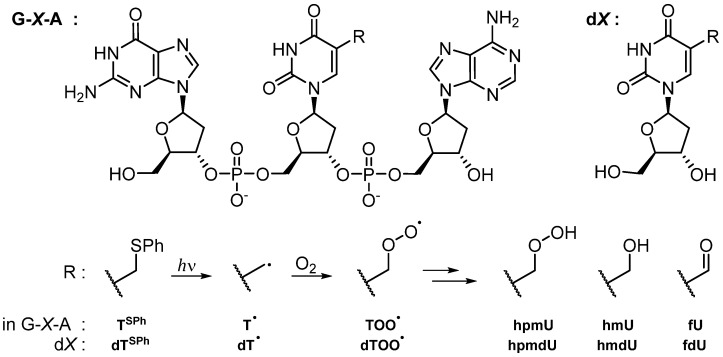
Structures of products analyzed in this study. The independent generation of 5-(uracilyl)-methylperoxyl radicals is possible by incorporation of a photolabile phenylsulfide precursor at the allylic position of thymine, either within the trinucleotide G–T–A or thymidine.

In a previous investigation [9], we studied the reactivity of the 5-(uracilyl)methyl peroxyl radical (TOO^•^), one of the most abundant peroxyl radicals formed upon exposure of DNA to HO^•^ generating systems [10,11,12]. Using a photolabile precursor incorporated in a trinucleotide with the sequence 5′-d(GpTpA)-3′ (G–T–A), it was possible to analyze in detail the fate of independently generated TOO^•^ (Figure 1). We showed that TOO^•^ undergoes addition to 5′-flanking guanines, leading to the formation of three tandem lesions, which consist of 8-oxo-7,8-dihydroguanine (oxoG) in tandem with either 5-formyluracil (fU) or 5-hydroxymethyluracil (hmU), as well as 2,4-diamino-5-formamido-pyrimid-6-one (fapyG) in tandem with fU. The effect of redox active reagents, e.g., Fe^3+^ and dithiothreitol (DTT), on the formation of tandem lesions was examined. The low rate constant estimated for the addition of TOO^•^ to guanine underlines the importance of small-molecule antioxidants to effectively compete against the formation of deleterious tandem lesions. Other biologically relevant RTA that react with ROO^•^ in the aqueous phase include l-ascorbate (Asc) and thiol-containing compounds, such as glutathione (GSH).

**Scheme 1:**
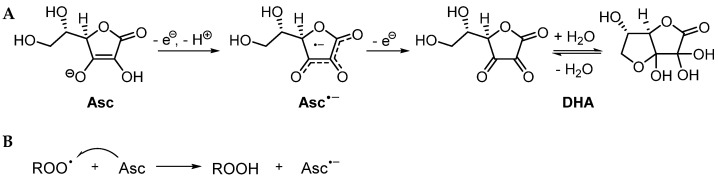
(**A**) Oxidation of ascorbate (Asc) by electron transfer leads to the ascorbyl radical anion (Asc^•−^). This intermediate decays further by one-electron oxidation or disproportionation, ultimately producing dehydroascorbate (DHA) that exists in solution as a hydrated cyclized hemiacetal. (**B**) The conventional mechanism of peroxyl radical trapping by Asc involves electron transfer followed by protonation to afford a hydroperoxide.

The main function of Asc in biology is as an antioxidant and redox cofactor in numerous biochemical reactions, including the biosynthesis of collagen, neurotransmitters, and the demethylation of epigenetic marks within histones and DNA [13,14,15]. The scavenging properties of Asc can be ascribed to its relatively elevated concentration in most cells and high rate constants of reaction with a variety of radicals. As illustrated in Scheme 1A, one-electron oxidation of Asc generates the ascorbyl radical that rapidly deprotonates (pKa < 0) [16,17] into the ascorbyl radical anion (Asc^•−^). Although the latter readily reacts with other radical species, it is a stable radical delocalized over three carbonyl groups and typically undergoes dismutation into Asc and its oxidized congener, l-dehydroascorbate (DHA). The chemistry of Asc^•−^ has been studied in detail using various one-electron oxidants, notably metal ions [18,19,20,21,22]. Likewise, numerous reports in the literature invoke single electron transfers with concurrent formation of Asc^•−^ as the underlying mechanism in the trapping of a wide range of oxygen radicals by Asc, including: hydroxyl radicals (HO^•^), superoxide radical anions (O_2_^•−^), alkoxyl radicals (RO^•^), peroxyl radicals (ROO^•^), and aryloxyl radicals (ArO^•^) [23,24,25,26,27,28,29,30,31,32,33]. Accordingly, the specific reaction between Asc and ROO^•^ affords the corresponding hydroperoxides (ROOH) (Scheme 1B).

Asc can also act as a pro-oxidant under certain circumstances. This effect arises from the ability of Asc to recycle Fe^2+^ or other redox active metal ions (Fe^3+^ + Asc → Fe^2+^ + Asc^•−^ + H^+^), which produces O_2_^•−^ and H_2_O_2_ in the presence of O_2_ that in turn can lead to the generation of HO^•^ as a potent oxidant by the well-known Fenton reaction (Fe^2+^ + H_2_O_2_ → Fe^3+^ + HO^−^ + HO^•^) [23,34,35,36]. Thereby, Asc can catalyze the oxidation of biomolecules in solution. Notably, the pro-oxidant effect of Asc can be observed in laboratory experiments due to the presence of trace amounts of Fe^2+^ and Cu^+^. Indeed, a common method to verify the presence of trace metals in aqueous solutions is the “ascorbate test” in which the decomposition of Asc as a function of time depends on the amount of transition metal ions [25]. The above properties of Asc have led to controversial results with in vitro and in vivo studies. For example, Asc was reported to induce the formation of potentially toxic lipid-derived electrophiles via the conversion of ROOH into highly reactive RO^•^ [37]. Similarly, numerous studies have reported on the toxicity of Asc in cell culture, particularly with cancer cells [38,39,40,41]. However, findings such as these can be attributed to artifactual oxidation due to the ability of Asc to recycle transition metals as well as react with components of cell-culture media such that high amounts of H_2_O_2_ (~100 µM) are generated extracellularly and taken up by cells [42,43]. Although evidence for potential detrimental effects in vivo under normal physiological conditions remains inconclusive in nutritional studies regarding vitamin C supplementation [44,45], Asc may act as a pro-oxidant at supraphysiological levels in plasma (1–2 mM), which is achieved by intravenous administration, a procedure that is under intense investigation as an alternative treatment of cancer [46,47].

In the present study, we investigate the reactivity of TOO^•^ in greater detail and report a novel reaction pathway of ROO^•^ scavenging by Asc that differs from the conventional single electron transfer mechanism.

## 2. Materials and Methods

All chemicals were of the highest purity and purchased from Sigma-Aldrich (St. Louis, MO, USA) unless stated otherwise, except thymidine (Chemgenes, Wilmington, MA, USA), 2′-deoxyuridine (Fisher Scientific, Waltham, MA, USA), ascorbic acid (VWR International, Radnor, PA, USA). The chemicals used for analyses by LC-MS/MS, i.e., ammonium formate, formic acid, and water (Fisher Scientific – Optima, Waltham, MA, USA), were LC-MS grade. NMR measurements were performed using a Bruker 400 MHz (Billerica, MA, USA). Purifications of products by HPLC were carried out using an Alliance system (Waters 2695, Milford, MA, USA) connected to a photo diode array detector (Waters 996) and controlled by a Millenium workstation (Waters version 4) using a semipreparative Luna Omega Polar C18, 250 Å, 5 µm, 10 mm × 250 mm column (Phenomenex, Torrance, CA, USA) operating at a flow rate of 3 mL/min with mobile phase systems consisting of 90% acetonitrile for eluent B and an aqueous eluent A. Elution programs and composition of eluent A are described where appropriate. High resolution mass spectrometry (HRMS) was performed by infusion on an ESI-Q-TOF (maXis) instrument (Bruker, Billerica, MA, USA).

**LC-MS/MS analyses.** Analyses of products were carried out by ultra-high-performance liquid chromatography (LC) online with tandem mass spectrometry (LC-MS/MS; API 3000 with Turbo Ionspray, AB-Sciex, Concord, Canada). The LC system (Nexera X2, Shimadzu, Kyoto, Japan) consisted of dual pumps (Model 30AD), an autosampler (SIL 30AC), a column oven (CTO-10AC), and an UV/Vis detector (SPD-20A) set at 260 and 220 nm. The chromatography was performed on an Acquity BEH C18, 130 Å, 1.7 µm, 2.1 mm × 100 mm column (Waters, Milford, MA, USA) protected by a pre-column of the same material, operating at a flow rate of 0.5 mL/min. The mobile phase programs consisted of a linear gradient of eluent B (90% acetonitrile) in eluent A (composition depending on the method) followed by a 2 min wash cycle with 95% eluent B and 3 min equilibration back to initial conditions. Modified nucleosides were separated using an elution program going from 2.5–12% eluent B over 4.5 min in eluent A composed of 0.1% formic acid + 0.5 mM ammonium formate. The MS/MS transitions used for MRM analyses are included in Table S1. Derivatives of Asc-PA were separated using an elution program going from 6% to 42% eluent B over 6 min in eluent A composed of 5 mM ammonium formate + 0.02% formic acid. The MS/MS transitions used for multiple reaction monitoring (MRM) analyses are included in Table S2.

**Synthesis of 5-hydroperoxymethyl-2′-deoxyuridine (hpmdU).** The nucleoside hpmdU was prepared on a small scale by menadione photosensitization [48,49]. Briefly, 20 µL of a 50 mM solution of 2-methyl-l,4-naphthoquinone dissolved in acetonitrile was added to a 25 mM aqueous solution of thymidine. The mixture was exposed for 15 min to near-UV light emitted from two medium-pressure 200-W Hg lamps with continuous O_2_ bubbling. The product was purified twice by HPLC using a gradient of eluent B going from 2% to 20% over 12 min in eluent A composed of 0.1% formic acid. Calibration of the product was created with UV absorption spectrometry using the extinction coefficient of hmdU (λ_264_, ε = 10.5) [10].

**Synthesis of 6-*O*-(phenylacetyl)-l-ascorbic acid (Asc-PA) and 5-*O*-(phenylacetyl)-l-ascorbic acid.** Asc-PA was synthesized using a previously published procedure with minor modifications [50]. l-Ascorbic acid (1.76 g, 10 mmol) and phenylacetic acid (1.36 g, 10 mmol) were added to 10 mL of 98% H_2_SO_4_ and the mixture was stirred vigorously at room temperature for 18 h. The dark solution was poured onto crushed ice and mixed with ethyl acetate. The aqueous layer was then extracted 3 times with ethyl acetate. The combined fractions were concentrated under reduced pressure, and the product was precipitated by addition of hexanes. 6-*O*-(phenylacetyl)-l-ascorbic acid was recrystallized twice in acetone/hexane giving 50% approximate yield (see characterization; Figures S1–S4, S32 and S33). Small quantities of 5-*O*-(phenylacetyl)-l-ascorbic acid were obtained by HPLC purification of the dried ethyl acetate fractions using an elution program going from 5% to 65% eluent B within 20 min in eluent A composed of 0.1% formic acid.

**Synthesis of l-dehydroascorbate (DHA) and 6-*O*-phenylacetyl-l-dehydroascorbate (DHA-PA).** DHA and DHA-PA were prepared according to the procedure of Washko et al. [51] by mixing 7 µL of Br_2_ to 1 mL of a 100 mM solution of ascorbic acid or Asc-PA, then flushed with air for 10 min. Stoichiometric yields are assumed based on the complete regeneration of Asc by DTT reduction at pH 7. Solutions of DHA and DHA-PA were always prepared fresh and neutralized with phosphate buffer before use.

**Synthesis of 2-*O*-oxalyl-4-*O*-(phenylacetyl)-l-threonic acid (Oxa-Thr-PA).** The preparation of Oxa-Thr-PA was readily performed by treating Asc-PA (14.7 mg, 50 µmol) with Oxone (95 mg, 155 µmol) in 30% acetonitrile in the presence of 100 mM phosphate buffer at pH 7.4 [52]. Full conversion was complete after 2 h. In the absence of buffer (at lower pH), the reaction proceeded more slowly but led to decreased isomerization. The product was purified by HPLC using an elution program going from 1% to 50% B within 30 min and held at 50% for 5 min, in eluent A composed of 10 mM ammonium formate + 0.05% formic acid.

**Synthesis of 4-*O*-(phenylacetyl)-l-threonic acid (Thr-PA).** Thr-PA was prepared using the same procedure as that of Oxa-Thr-PA, except the reaction was performed at pH 10 and heated at 60 °C for 5 h [52].

**Synthesis of 1,2-*O*-cyclo-oxalyl-4-*O*-(phenylacetyl)-l-threonate (cOxa-Thr-PA).** The preparation of cOxa-Thr-PA in the absence of water was achieved by reacting Asc-PA (10 mg, 34 µmol) with 25 mg of mCPBA (≤77%) in freshly distilled acetone for 3 h at room temperature [52]. For MS analyses, the reaction mixture was diluted in dry acetonitrile. To obtain the NMR spectra of the product, the reaction was performed instead in (CD_3_)_2_CO (99.9 atom-% D) and analyzed directly. The preparation of cOxa-Thr-PA in aqueous solution was also possible with the method employed for the preparation of Oxa-Thr-PA in the absence of buffer and by limiting the reaction time to 15 min. Using the same HPLC elution program, the product eluted after 30 min.

**Synthesis of 5,6-O-isopropilidene-l-ascorbic acid (Asc-iPr).** Asc-iPr was synthesized according to the method of Jackson and Jones [53].

**Photolysis.** UV irradiation was carried out using a 1000 W Hg-Xe arc lamp (Spectra Physics, Oriel Instruments, Stratford, CT, USA) fitted with a water filter and diffracting monochromator (Spectral Energy Corp., Chester, NY, USA) set at 260 nm. The beam of light was focused on a quartz cuvette (1 cm path length). The average number of incident photons was 4.0 × 10^15^ photons s^−1^ as determined by a photometer (Industrial Fiber Optics, Inc., Tempe, AZ, USA). The solutions were bubbled with O_2_ for 5 min prior to and during photolysis.

## 3. Results

### 3.1. Fate of 5-(Uracilyl)Methyl Peroxyl Radicals in the Presence of Ascorbate

#### 3.1.1. Single and Tandem Lesions Formation in a Trinucleotide Model

**Figure 2:**
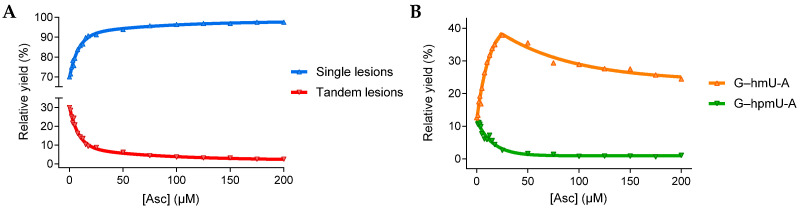
Distribution of lesions obtained by LC-MS/MS arising from TOO^•^ within G–T–A as a function of Asc concentration. (**A**) Total single and total tandem lesions. (**B**) Formation of ROOH (G–hpmU–A; green curve) and ROH (G–hmU–A; orange curve).

The selective photoinduced generation of TOO^•^ within G–T–A (Figure 1) and the analysis of associated lesions by LC-MS/MS were performed as described [9]. The phenylsulfide photocleavable system allows one to investigate the specific reactions of TOO^•^ with reducing agents that produce single modifications of thymine in competition with the addition to vicinal guanine that leads to tandem base modifications. When TOO^•^ was generated in the absence of Asc, single and tandem lesions accounted for approximately 70% and 30% of products, respectively. The addition of Asc efficiently blocked the production of tandem lesions, with less than 25 µM required to decrease their formation to below 10% of the total products (Figure 2A). To our surprise, the formation of the expected ROOH product 5-hydroperoxymethyluracil (hpmU) was minor and decreased to near zero levels from 0 to 25 µM Asc, while the formation of hmU greatly increased (Figure 2B). The kinetics of hmU formation as a function of Asc matched the kinetics of single lesion formation, indicating that the reaction of Asc with TOO^•^ leads to hmU rather than hpmU. These results contradicted the classical mechanism of electron transfer from Asc to ROO^•^ to produce ROOH. Thus, a series of experiments including mechanistic studies were undertaken to better understand this phenomenon.

#### 3.1.2. Reactivity in a 2′-Deoxyribonucleoside Model

**Figure 3:**
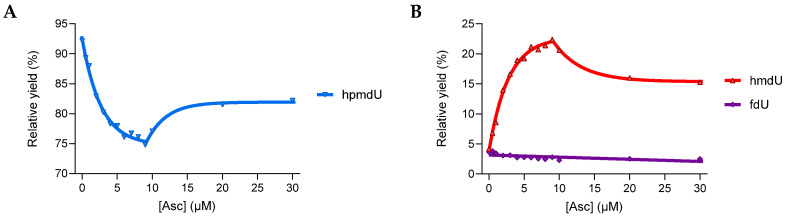
Distribution of products obtained by LC-MS/MS arising from 2′-deoxynucleoside dTOO^•^ as a function of Asc concentration. (**A**) Formation of ROOH (hpmdU; blue curve). (**B**) Formation of ROH (hmdU; red curve) and aldehyde (fdU; purple curve).

To simplify the chemistry, the trinucleotide system was changed to a 2-deoxyribonucleoside monomer model (d*X* in Figure 1). This eliminates reactions with vicinal nucleotides and allows for the accurate quantification of 5-hydroperoxymethyl-2′-deoxyuridine (hpmdU), which is relatively stable as a free nucleoside [49]. The photolabile precursor homologue 5-phenylthiomethyl-2′-deoxuridine (dT^SPh^) was used to selectively generate 5-(2′-deoxyuridinyl)methylperoxyl radicals (dTOO^•^). Product analysis carried out under control conditions (dT^SPh^ concentration = 125 µM, 2 min photolysis, phosphate-buffered (pH 7.4), O_2_-saturated solutions) showed that methyl oxidation products of thymidine amount to approximately 5 µM, affording hydroperoxide hpmdU as the predominant product (93%) while the formation of 5-hydroxymethyl-2′-deoxyuridine (hmdU) and 5-formyl-2′-deoxyuridine (fdU) was minor (ca. 4% each). The high yield of hpmdU in control experiments likely arises from the reduction in dTOO^•^ by intermediate products derived from thiophenyl photocleavage. In the presence of 10 µM Asc, however, the relative yield of fdU remained unchanged, whereas hmdU increased from 4 to 22% and hpmdU dropped from 93 to 75%, a difference of 18% (Figure 3). Thus, the results indicate that Asc modulates the distribution of products by diverting the conventional pathway giving hpmdU to a novel pathway involving the formation of hmdU. This pathway also accounts for the slight increase in hpmdU observed at higher concentrations of Asc (10–30 µM).

### 3.2. Mechanistic Studies

Several mechanisms can tentatively explain the unconventional Asc-mediated production of alcohols (ROH) from initial ROO^•^. The following section details our investigation of 3 possible mechanisms that are summarized in reaction pathways 1, 2, and 3.

#### 3.2.1. Possible Mechanism #1: Electron Transfers Involving Intermediate Alkoxyl Radical

The radical trapping character of Asc is generally attributed to its low oxidation potential that allows it to undergo single-electron transfers with reactive species. According to this well-established mechanism, a series of single-electron transfers may explain the formation of ROH from ROO^•^ (Pathway 1). First, Asc reduces ROO^•^ to ROOH (R1). Next, the hydroperoxide reacts with a second electron in a Fenton-type cleavage, generating an intermediate alkoxyl radical (RO^•^) (R2). Lastly, Asc reduces RO^•^ to provide ROH (R3). To clarify if the proposed mechanism accurately explains the formation of ROH from ROO^•^, the chemistry of RO^•^ was investigated independently.

**Pathway 1**

(R1)
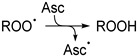

(R2)
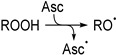

(R3)
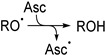


**Figure 4:**
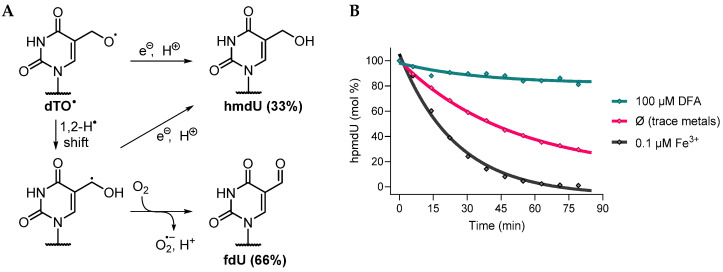
(**A**) Fate of the 5-(2′-deoxyuridinyl)methoxyl radical under reducing conditions. Reaction of 5 µM hpmdU with either 100 µM Fe^2+^ or Asc (0.1–5 mM) without DFA yields fdU and hmdU in a 2:1 ratio. (**B**) Ascorbate-induced decomposition of hpmdU. Phosphate-buffered (pH 7.4) solutions containing 5 µM of hpmdU were treated at 37 °C with 100 µM Asc either in the absence of additive (pink curve), in the presence of 100 µM DFA (green curve), or in the presence of 0.1 µM Fe^3+^ (black curve).

A suitable procedure to generate RO^•^ consists of treating ROOH with one-electron reductants, such as Fe^2+^, in a so-called Fenton-type reaction. With this method, the specific chemistry of 5-(2′-deoxyuridinyl)methoxyl radicals (dTO^•^) was investigated starting with hpmdU. It should be noted that HPLC purified hpmdU has a relatively long lifetime in aqueous solution (months at 5 °C), and thus, thermal decomposition does not contribute to the formation of products during our experiments [49]. Treatment of 5 µM hpmdU with 20 equivalents of Fe^2+^ consumed the compound instantaneously and gave a mixture of fdU and hmdU in a 2:1 ratio. The high relative yields of fdU can be explained by the propensity of alkoxyl radicals to undergo fast unimolecular decay in competition with intermolecular proton-coupled electron transfer. Here, the formation of fdU from dTO^•^ is rationalized by a 1,2-H-atom shift followed by O_2_ oxidation of the ensuing ketyl radical (Figure 4A) [54,55,56,57,58,59,60,61]. Alternatively, 2′-deoxyuridine (dU), the expected product of C–C β-scission of dTO^•^, did not form in the reaction mixtures.

Next, the ability of Asc to react with hpmdU was assessed. The reaction of 5 µM hpmdU with 20 equivalents of Asc afforded an identical ratio of products but with much slower kinetics (*t*_1/2_ between 17 and 35 min). The same 2:1 carbonyl-to-alcohol ratio obtained in this transformation indicates that the reaction proceeds via a Fenton-type cleavage of the O–O bond of hpmdU. Higher concentrations of Asc (0.1–5 mM) had no effect on product distribution. Our results are consistent with a recent study investigating the chemistry of cholesterol alkoxyl radicals generated in the presence of Asc, which observed high carbonyl-to-alcohol ratios of products [62]. However, the addition of a strong chelator of iron, i.e., deferoxamine (DFA; 100 µM), drastically inhibited the Asc-induced decomposition of hpmdU, while the addition of 0.1 µM Fe^3+^ greatly accelerated it (Figure 4B). Thus, one can conclude that the decomposition of hpmdU by Asc occurs primarily through catalysis by metal ions present in trace amounts in aqueous solutions. A complete inhibition of hpmdU decomposition in the presence of DFA demonstrates that Asc does not undergo direct electron transfer with the hydroperoxide to generate dTO^•^ (R2). Previous claims that report a direct reaction of hydroperoxides with Asc are likely incorrect due to the presence of trace metal ions in buffered solutions [37]. Furthermore, because fdU is a prominent and characteristic product of dTO^•^, and the formation of fdU was not observed in the reaction of dTOO^•^ with Asc (Figure 3B), the intermediacy of dTO^•^ may be excluded as a possible mechanism. In conclusion, our results show that the mechanistic Pathway 1 does not explain the formation of ROH from ROO^•^ in the presence of Asc.

#### 3.2.2. Possible Mechanism #2: Peroxyl Radical Addition

Peroxyl radicals display various uni- and bi-molecular decomposition pathways. The conversion of ROO^•^ into ROOH via proton-coupled electron transfer takes place by either a stepwise mechanism (i.e., one-electron reduction followed by protonation) or a concerted mechanism (i.e., formal H-atom transfer (HAT)). The former pathway is conventionally ascribed to reactions of ROO^•^ with Asc, while the latter one is involved in the chain propagation step of lipid peroxidation and scavenging reactions by thiols (e.g., GSH) and tocopherol. On the other hand, peroxyl radical addition (PRA) to double bonds represents a major pathway that often competes with HAT [63], as exemplified by the autoxidation of cholesterol [64]. In DNA, a similar competition arises between intramolecular PRA to vicinal guanine and intermolecular HAT with protective thiols [8]. PRA leads to the generation of peroxide adducts, i.e., endoperoxyl radicals, whose fate depends on the chemical structure of the adduct and can be modulated by the presence of redox agents. For instance, in our previous study involving the addition of TOO^•^ to 5′-flanking guanine, the formation of three distinct tandem modifications was shown to originate from the subsequent reactions of a common guanine-thymine endoperoxyl radical intermediate [9]. In this case, the guanine-centered radical undergoes either oxidation or reduction due to its redox ambivalence (illustrated in Scheme 5 of Ref. [8]). A similar series of reactions can be proposed for Asc (Pathway 2).

**Pathway 2**

(R4)
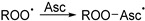

(R5)
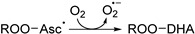

(R6)
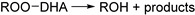


In theory, the addition of ROO^•^ to the enediol portion of Asc results in the formation of a ketyl radical anion (R4). Ketyl radicals are a type of species known to rapidly undergo oxidation in aerated conditions to the corresponding carbonyl-containing product via O_2_ addition followed by HO_2_^•^ elimination [56,57,58,62]. The fast elimination of HO_2_^•^ facilitated by the α-hydroxyl group generally prevents subsequent reactions by intermediate peroxyl radicals [65,66,67,68,69,70]. Additionally, the rate constant of O_2_^•−^ elimination from α-hydroxyalkylperoxyl radical *anions* (i.e., with deprotonated α-OH groups) is accelerated by multiple orders of magnitude compared to their protonated counterparts [66,71]. Consequently, it is reasonable to propose initial PRA to Asc followed by oxidation of ROO-Asc^•^ ketyl radical anions by O_2_ providing nonradical peroxide adducts (R5).

**Scheme 2:**
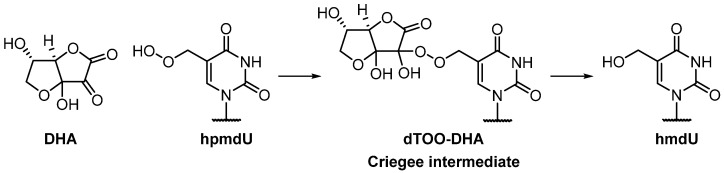
Proposed reaction between hpmdU and DHA giving rise to hmdU via the formation of a Criegee intermediate.

**Figure 5:**
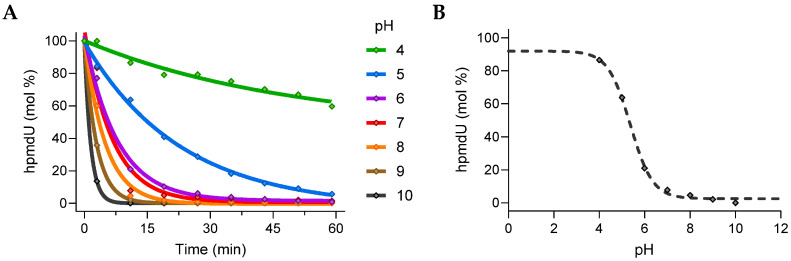
(**A**) Kinetics of decomposition of hpmdU (5 µM) by DHA (750 µM; 150 eq.) in the pH range 4–10. (**B**) Sigmoidal representation of the effect of pH on the kinetics of the reaction (pH_V50_ = 5.4).

The proposed ROO–DHA adducts are structurally analogous to so-called Criegee intermediates (*gem*-hydroxy peroxides) arising from the nucleophilic addition of hydroperoxides/peroxyacids to aldehydes and ketones that can subsequently undergo Baeyer-Villiger rearrangements [52,72]. To determine whether this pathway gives rise to the major alcohol product hmdU (R6), the reaction between hpmdU and DHA was examined independently (Scheme 2). As predicted, treatment of hpmdU with a very large excess of DHA (150 eq.) induced its complete transformation into hmdU (95% yield). The use of lower concentrations of DHA led to decreased rates of conversion, probably because of its fast hydrolysis at neutral pH (*t*_1/2_ = 22 min [73]). The implication of a Baeyer-Villiger rearrangement is supported by an acceleration of the reaction rate as a function of pH (Figure 5). At pH > 7, conversion of hpmdU into hmdU was complete in approximately 10 min, whereas acidic conditions retarded the reaction considerably (*t*_1/2_ = 75 min at pH 4). Similar to Dakin reactions [74], base catalysis observed here is rationalized by deprotonation of the *gem*-OH group of a Criegee intermediate, which accelerates migration to the peroxide, the rate-limiting step [75]. From the inflection point of the pH curve (Figure 5B), it is reasonable to assume that the p*K*a of the *gem*-OH of dTOO–DHA is approximately 5.4. The above results demonstrate that ROO–DHA adducts undergo rearrangement to provide ROH products, thus supporting reaction R6 of Pathway 2. The formation of hmdU from the reaction of dTOO^•^ with Asc under our conditions does not arise from the nonradical addition of hpmdU to DHA because very little amounts of DHA are formed in photolysis mixtures (<10 µM). Furthermore, no conversion was observed when 5 µM hpmdU was treated with 10 µM of DHA for 10 min under similar conditions as those during photolysis. A substantial excess of DHA (>10 eq.) is necessary for the nonradical reaction to proceed. Thus, we conclude that both the reaction between hpmdU and DHA and the one between dTOO^•^ and Asc lead to a common intermediate (dTOO–DHA Criegee adduct) and that the formation of hmdU is explained by the subsequent decomposition of this intermediate via a Baeyer–Villiger rearrangement.

#### 3.2.3. Possible Mechanism #3: Peroxyl Radical Addition Followed by Epoxide Formation

Following PRA, the resulting endoperoxyl radicals can subsequently undergo various types of reactions depending on the redox properties of the radical and the chemical environment. For example, carbon-centered radicals generated by PRA to alkenes engage in two competing pathways: bimolecular addition of O_2_, affording additional peroxyl radicals, or unimolecular decay via O–O homolytic substitution with concomitant formation of RO^•^ and an epoxide [76,77,78]. These reactions are showcased in the oxidation of various lipids [64,79,80]. The latter pathway may be proposed as an alternative explanation for the Asc-induced formation of ROH from initial ROO^•^, as expressed in Pathway 3.

**Pathway 3**

(R4)
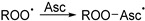

(R7)
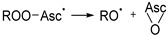

(R3)
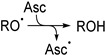

(R8)
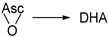


This pathway features PRA to the C2=C3 bond of Asc (R4), followed by O–O homolytic substitution, and the release of RO^•^ and epoxidized Asc (R7), which can decompose into ROH (R3) and DHA (R8), respectively. However, as already shown in the previous section, Asc-mediated transformation of dTOO^•^ into hmdU does not involve the intermediacy of alkoxyl radicals because dTO^•^ leads to the characteristic formation of fdU, and the yield of this product was negligible (Figure 3B). Thus, both Pathways 1 and 3 can be discarded leaving Pathway 2 as the most likely route that converts dTOO^•^ into hmdU.

### 3.3. Product Characterization

**Figure 6:**
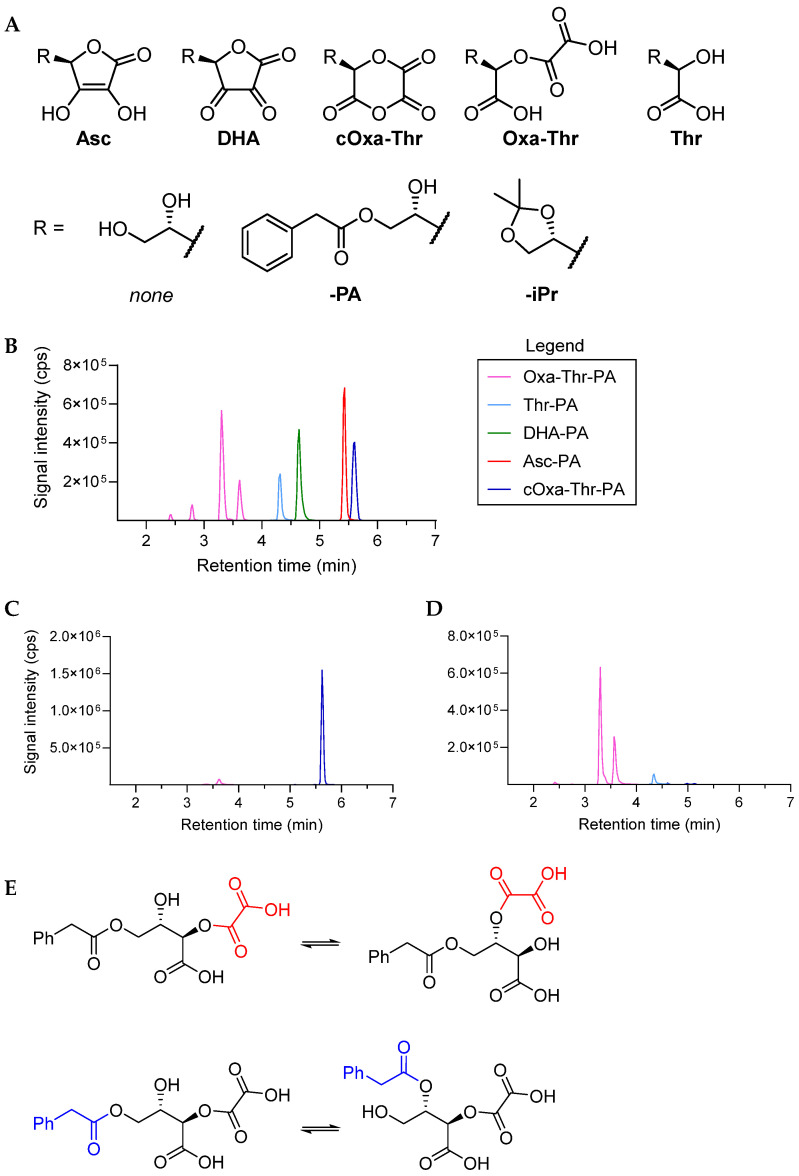
Analysis of Asc-PA derivatives by LC-MS/MS. (**A**) Structure of products identified from peroxide-mediated oxidations. (**B**) Representative chromatographic separation of products from the oxidation of Asc-PA. (**C**) Purification and immediate analysis of cOxa-Thr-PA and (**D**) its hydrolysis products following decomposition in phosphate-buffered solution (pH 7.4). (**E**) Example of reversible acyl shifts observed in Oxa-Thr-PA involving either the oxalyl group (red) or the phenylacetyl group (blue).

The oxidation of Asc was examined using the ester derivative 6-*O*-(phenylacetyl)-l-ascorbic acid (Asc-PA). The presence of a nonpolar substituent at C6 of Asc greatly improves the analysis of Asc-derived oxidation products with respect to their retention on reversed phase chromatography and detection by UV and MS. A mixture of oxidation products of Asc-PA was observed by LC-MS/MS following the independent generation of dTOO^•^ upon photolysis of dT^SPh^ in the presence of 50 µM Asc-PA (Figure 6B). The same products with a similar profile were also observed by treatment of Asc-PA with mild oxidants, including oxone (KHSO_5_), meta-chloroperoxybenzoic acid (mCPBA), and H_2_O_2_ in aqueous solutions at pH 7.4 (Figure 6). Of the products obtained, two are well-known oxidation products of Asc (products at 4.65 and 4.30 min; Figure 6B). The product at 4.65 min was identified as the 6-*O*-phenylacetate ester of dehydroascorbate (DHA-PA) on the basis of MS analysis (Figures S5–S11, S28 and Table 1) and comparison of the LC and MS properties with authentic compounds obtained by the reaction of Asc-PA and Br_2_ that quantitatively gives DHA-PA [51]. The identity of another common oxidation product of Asc, i.e., 4-*O*-(phenylacetyl)-l-threonic acid (Thr-PA; peak at 4.30 min in Figure 6B) was confirmed by MS (Figures S12–S15, S29 and Table 1) and NMR analysis (Figures S34–S37). The phenylacetyl group attached to Asc-PA, Thr-PA, and other products depicted the corresponding proton and carbon atoms of the substituent (Table 2). Based on ^1^H-^13^C heteronuclear analysis (HMBC), the carbonyl associated with the phenylacetyl group shows strong cross-peaks with the vicinal CH_2_ group (Figure S37). The presence and position of the carboxylic acid group of Thr-PA were confirmed by ^13^C-NMR showing a peak at 174.6 ppm and corresponding ^1^H-^13^C cross-peaks with H3 and H4 in the HMBC spectrum (Figures S36 and S37). These analyses are consistent with those reported in the literature on synthetic compounds [81].

In addition to known products, several unidentified peaks of variable stability were observed in the oxidation mixture of Asc-PA, with four of them having a mass of 326 Da (peaks at 2.4, 2.7, 3.3, and 3.6 min; Figure 6B). The least polar product at 5.6 min, which had a mass of 308 Da, was found to be particularly unstable. Following purification by HPLC and addition to a phosphate-buffered solution at pH 7.4, the latter compound rapidly converted into additional products found at 3.3 and 3.6 min (Figure 6C,D). Moreover, upon purification followed by its incubation at pH 7.4 and 37 °C, the major product at 3.3 min was found to give rise to the three other polar products with an equal molecular mass (peaks at 2.4, 2.7, and 3.6 min; Figure 6B) and a small amount of Thr-PA. *These results clearly show that the product at 5.6 min is the sole precursor of the four early eluting products.* Thus, we conclude that the initial product of oxidation of Asc-PA by dTOO^•^ is the product at 5.6 min (308 Da) and that this compound irreversibly undergoes hydrolysis (+18 Da) into the series of four polar isomers described above (326 Da; peaks at 2.4, 2.7, 3.3, and 3.6 min). Furthermore, the mass and corresponding fragment ions indicate that the early eluting products at 2.4, 2.7, 3.3, and 3.6 min are isomers, bearing an intact Thr-PA portion and an oxalate substituent. A very similar product profile was also observed from the reaction of other peroxide-based oxidants with Asc-PA (oxone and mCPBA).

The structures of unknown products were determined by extensive MS and NMR analyses. The product at 5.6 min was identified as the diglycolic anhydride derivative, namely, 1,2-*O*-cyclo-oxalyl-4-*O*-(phenylacetyl)-l-threonate (cOxa-Thr-PA). The exact mass (Table 1) and fragmentation of the parent compound, showing fragments at 235 *m*/*z* (M–OH–COCO_2_) and 189 *m*/*z* (M–PhCH_2_CO) in negative mode (Figures S18 and S30), support the proposed structure. To examine the structure by NMR, it was necessary to carry out the oxidation of Asc-PA *in situ* in the NMR tube using mCPBA as the oxidant in a non-nucleophilic solvent (deuterated acetone). The oxidation was complete under these conditions and afforded cOxa-Thr-PA (70%) together with a product from hydrolysis (30%) (Figures S38–S44). Although the spectra contained signals from excess mCPBA and meta-chlorobenzoic acid, the signals associated with cOxa-Thr-PA were clearly discernable from those of the reagent and byproducts that appeared in a specific aromatic region of the spectra. To simplify comparison between compounds in the remaining text, the numbering of atoms refers to carbon positions in the original Asc backbone. The NMR spectra of cOxa-Thr-PA were similar to those of Asc-PA and Thr-PA (Table 2) except that the protons corresponding to –CH_2_–CH(OH)–CH– of the Asc side chain (carbons from left to right are numbered C6, C5, and C4) were shifted to the lower field, likely due to deshielding caused by the cyclic oxalate substituent at C4 of the backbone. The carbonyl groups of the cyclic oxalate group at C1, C2, and C3 were assigned based on observable cross-peaks with H4 and H5 from 2D HMBC analysis (Figure S44). In addition, the assignment of C1 and C2 to the oxalate group of cOxa is supported by simulation of the ^1^H- and ^13^C-NMR spectra (Figure S52).

The major polar product at 3.3 min was identified as 2-*O*-oxalyl-4-*O*-(phenylacetyl)-l-threonic acid (2-Oxa-Thr-PA). This product was observed as a minor component (30%) in the spectra of cOxa-Thr-PA (Figure S38–S44). It was also purified by HPLC and subjected to detailed NMR analyses (Figure S45–S51; Table 2). Based on integration of CH_2_ protons of the phenylacetyl substituent, ^1^H-NMR analysis indicates that the purified product at 3.3 min transforms into a mixture of four products with a ratio of 65:20:8:7 (Figures S45 and S46). The relative proportion of each product aligns with the intensity of the four peaks observed previously by LC-MS/MS. The major product representing 65% of the mixture depicted the phenylacetyl substituent, the –CH_2_–CH(OH)–CH– side chain, as well as additional carbonyl groups associated with an oxalate substituent and a carboxylic acid (Figures S48–S51). In comparison to the major product, the product representing 20% of the mixture showed dramatic changes in the chemical shifts of H4 and H5. In particular, the chemical shift of H4 decreased from 5.23 to 4.56 ppm (−0.8 ppm), while that of H5 increased from 4.49 to 5.59 ppm (+1.1 ppm). In addition, the chemical shift of C4 decreased by −0.8 ppm while that of C5 increased by 1.3 ppm. These changes are consistent with the migration of a largely deshielding oxalate group from O4 to O5. Thus, the other major product in the mixture was identified as a regioisomer 3-*O*-oxalyl-4-*O*-(phenylacetyl)-l-threonic acid (3-Oxa-Thr-PA; Figure 6E). In addition to the major products at 3.3 and 3.6 min, two minor products that arise from isomerization of 2-Oxa-Thr-PA and that have the same mass were observed at 2.4 and 2.7 min by LC-MS/MS. To ascertain the structure of these products, we examined the oxidation of Asc bearing the phenylacetate group at C5 rather than C6 (i.e., 5-*O*-(phenylacetyl)-l-ascorbate rather than 6-*O*-(phenylacetyl)-l-ascorbate). In this case, the predominant product was identified by MS and HPLC as the same product that elutes at 2.4 min. Thus, we conclude that peaks at 2.4 and 2.7 min arise from migration of the phenylacetate group, i.e., either 1,4-shift for 2-Oxa-Thr-PA or 1,5-shift for 3-Oxa-Thr-PA (Figure 6E). The migration of oxalate and phenylacetate from one OH group to another results in a mixture of four regioisomers. The proportion of Oxa-Thr-PA isomers depends on the nucleophilicity of the OH group, such that substitution was more efficient for the primary alcohol at O6 relative to the secondary alcohol at O5. These findings were corroborated using 5,6-*O*-isopropylidene-ascorbate (Asc-iPr), in which both O5 and O6 are substituted by the derivatization group. Upon oxidation, the disubstituted derivative produced only one open-chain oxalate derivative, i.e., 2-*O*-oxalyl-3,4-*O*-isopropylidene-l-threonic acid (Oxa-Thr-iPr); the product nevertheless was slightly susceptible to hydrolysis into 3,4-*O*-isopropylidene-l-threonic acid (Thr-iPr). A final structural characterization was achieved by converting the carboxylic acid-containing phenylacetate derivatives into amides of phenylethanamine using EDC-mediated coupling methods (Figures S53–S67). This provided further support for the structures of Oxa-Thr-PA regioisomers, as well as threonate and oxalate.

In conclusion, the cyclic compound cOxa-Thr-PA was unambiguously identified as a novel intermediate in the peroxide-mediated oxidation of Asc-PA, as inferred by detailed MS and NMR analysis. In neutral aqueous solutions, this product rapidly hydrolyzes into the open chain Oxa-Thr-PA, which in turn can undergo acyl group migrations to yield a mixture of four regioisomers, as well as hydrolysis into Thr-PA. The identification of cOxa-Thr-PA validates the proposed mechanism presented in reaction Pathway 2, which features a key step involving a Baeyer–Villiger rearrangement following PRA to Asc.

### 3.4. Oxidation of Asc-PA by Peroxyl Radicals Derived from Azo Compounds

To further validate the novel reaction pathway, we examined the oxidation of Asc-PA by ROO^•^ derived from the thermal decomposition of azo compounds, i.e., 2,2′-azobis(2-amidinopropane) dihydrochloride (ABAP) and 2,2′-azobis(2-methylpropionitrile) (AIBN). Asc-PA (50 µM) was exposed at 37 °C to either ABAP (100 µM) in aqueous solution buffered at pH 7.4 in the presence of DFA (100 µM), or AIBN (100 µM) in acetonitrile (unbuffered). As expected, we observed a time-dependent formation of Oxa-Thr-PA in the aqueous reaction mixture, and cOxa-Thr-PA in the organic reaction mixture, with minute amounts of hydrolyzed products (Figure S68). These results confirm that the PRA reaction to Asc occurs for alkylperoxyl radicals generated by ABAP and AIBN.

## 4. Discussion

Ascorbate is an effective antioxidant in vivo because it scavenges reactive species with high rate constants of reaction, it usually exists at elevated intracellular concentrations, and it is rapidly recycled upon oxidation. The reactivity of Asc toward radicals is commonly attributed to its ability to donate electrons, like its reaction with oxidizing metal ions. However, pioneering work demonstrated that Asc oxidation by HO^•^ does not exclusively afford Asc^•−^ via direct electron transfer but rather produces a mixture of ascorbate radicals, including C2=C3 addition products in considerable proportions [16,82,83,84,85,86]. Likewise, a very detailed kinetic analysis by Cabelli and Bielski showed that the reaction between Asc and O_2_^•−^ does not properly generate Asc^•−^; instead, they proposed the formation of a “*transient adduct or product*” which rapidly engages in redox reactions involving either O_2_^•−^ or disproportionation [87]. These studies, along with the present one, reflect the unusual reactivity of Asc toward oxygen radicals and demonstrate that, besides electron transfer, addition reactions are involved in radical trapping by Asc. Peroxyl radical addition to double bonds is a well-known phenomenon, especially in the context of hydrocarbon autoxidation. The present study provides direct evidence of PRA to support product analysis and mechanistic studies.

Our results show that a reaction between a peroxyl radical (i.e., dTOO^•^) and Asc leads to the distinctive formation of an alcohol (i.e., hmdU). The intermediacy of an alkoxyl radical in the reaction sequence is ruled out because of the lack of formation of the corresponding carbonyl product (i.e., fdU). The absence of a carbonyl product excludes alternative mechanisms: stepwise electron transfers involving O–O bond cleavage by one-electron reduction (reaction Pathway 1), and PRA to Asc followed by unimolecular decay via radical epoxidation (reaction Pathway 3). To explain the formation of an alcohol, we propose PRA followed by O_2_-mediated oxidation and Baeyer–Villiger rearrangement of the resulting peroxide adduct (reaction Pathway 2). This mechanism is substantiated by the modulating effect of pH on the rate of rearrangement and unambiguous identification of the ring-expansion product, cOxa-Thr. Furthermore, our results clarify the chemistry of Asc oxidation as well as the reaction of DHA with H_2_O_2_, which afford oxalate esters of threonic acid (Oxa-Thr) and threonic acid (Thr) as observed in earlier reports [88,89,90,91]. Previously, Fry and coworkers proposed a cyclic oxalate intermediate to explain the oxidation of Asc and DHA by H_2_O_2_ [90,91]. They hypothesized that this intermediate undergoes decomposition into three open-chain oxalyl-threonate derivatives that interconvert via oxalate migration (note that the authors studied unsubstituted Asc with a free hydroxyl group at C6 that can participate in migration). The latter oxidation products of Asc were tentatively identified based uniquely on their electrophoretic and chromatographic properties and comparison to threonate and oxalate derivatives. Although the structure of the cyclic oxalate intermediate proposed by Fry and colleagues was incorrect, the basic structure of the more stable open-chain threonate derivatives compares well with those deduced from our complete analysis.

**Scheme 3:**
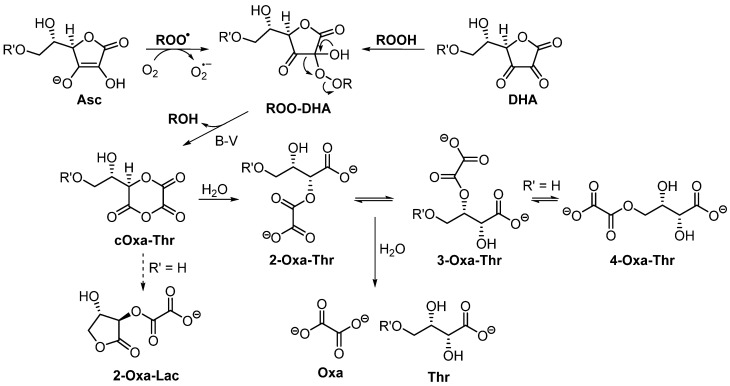
Proposed mechanism for the oxidation of Asc via PRA, and DHA by either hydroperoxides or H_2_O_2_, involving Baeyer–Villiger rearrangement that generates cOxa-Thr. Subsequent hydrolysis of cOxa-Thr produces open-chain Oxa-Thr regioisomers. R’ = H, Asc derivatives; R’ = COCH_2_Ph, 6-*O*-phenylacetate derivatives.

Here, we show that either reactions of Asc with ROO^•^, or DHA with ROOH and H_2_O_2_, lead to a Criegee intermediate that undergoes a Baeyer–Villiger rearrangement to generate cOxa-Thr. This intermediate hydrolyzes into 2-Oxa-Thr, which interconverts via acyl migration into 3-Oxa-Thr and the more stable 4-Oxa-Thr if this position is unsubstituted. Further hydrolysis of the latter oxalate esters leads to the formation of two final decomposition products: threonic acid and oxalic acid. It is reasonable to assume that a similar mechanism takes place not only for 6-*O*-(phenylacetyl)-ascorbate but also for the nonderivatized Asc in its biologically active form. Thus, we propose a revised mechanism for the oxidation of Asc by peroxyl radicals and other peroxides shown in Scheme 3. Fry and coworkers also proposed the formation of an additional compound arising from H_2_O_2_ oxidation, namely l-threonolactone [91]. Based on our revision of the chemistry, we suggest that this product arises from an alternative mechanism involving intramolecular cyclization of unsubstituted C6-OH of cOxa-Thr to produce 2-*O*-oxalyl-l-threonolactone (2-Oxa-Lac) in competition with hydrolysis that gives Oxa-Thr isomers.

It is unclear whether PRA to Asc occurs at position C2 or C3, since both are probable and would lead to the formation of cOxa-Thr. On one hand, addition at C3 generates a stable radical that is delocalized through C1 and C2, compared to the C2 adduct that simply generates a radical anion. On the other hand, nucleophilic addition of hpmdU to DHA likely takes place at the more electron-deficient C2 position, which gives an intermediate that demonstrably undergoes Baeyer–Villiger rearrangement leading to hmdU. It has been previously shown that HO^•^ addition to Asc can occur at both C2 and C3 [85,86]. Thus, the addition of ROO^•^ may occur at both sites, although it is likely more selective than HO^•^ suggesting that addition to the C2 position is the most probable.

**Scheme 4:**
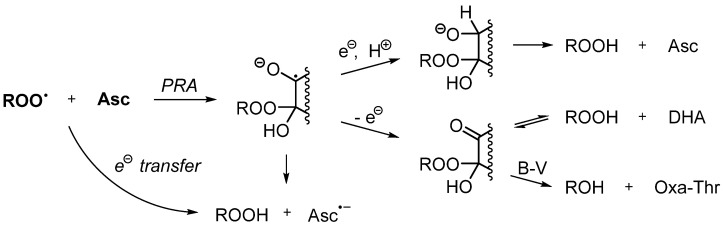
Reaction pathways of ROO^•^ with Asc involving either conventional electron transfer that produces ROOH and Asc^•−^, or peroxyl radical addition (PRA). Oxidation of the PRA adduct by O_2_ leads to the formation of either ROH and Oxa-Thr via Baeyer–Villiger rearragement or ROOH and DHA via dissociation. Conversely, reduction in the PRA adduct by another molecule of Asc may lead to release of ROOH by dissociation with concomitant regeneration of initial Asc.

The fate of ROO–Asc^•^ PRA adducts may involve alternative pathways that were not evaluated in the present study (depicted in Scheme 4). Firstly, it is possible that ROO–Asc^•^ undergo unimolecular decay into Asc^•−^ and peroxide anions, which are the same products obtained via the classical electron transfer mechanism. Secondly, we have shown that O_2_-mediated oxidation of ROO–Asc^•^ (R4 in Pathway 2) ultimately leads to the formation of an alcohol via a key Baeyer–Villiger rearrangement. However, the Criegee intermediate required for this reaction is in equilibrium with the dissociation products, which include ROOH and DHA. Thus, the effectiveness of the formation of ROH is limited by both the rate of ROO^•^ addition to Asc and the rate of dissociation of ROO–DHA adducts. Moreover, in parallel with previous propositions related to O_2_^•−^ and Asc adducts [87], the oxidation ROO–Asc^•^ may compete with their reduction. A gradual increase in hmpdU accompanied with a loss of hmdU at high concentrations of Asc (>10 µM) (Figure 2B and Figure 3B) can be explained by the reduction in PRA adducts by Asc. Reduction in ROO–Asc^•^ will prevent the Baeyer–Villiger rearrangement necessary for the formation of ROH and divert the pathway to the formation of ROOH, the same product that arises from initial electron transfer.

Because both Asc and Asc^•−^ undergo electron transfer with reactive species according to the classical mechanism (Scheme 1A), the stoichiometry of peroxyl radical trapping theoretically involves two ROO^•^ for the oxidation of Asc to DHA. Inferior values have been obtained experimentally, decreasing as low as ~0.1 [29,85,92,93]. Such small values are generally rationalized in terms of Asc depletion by trace metal-catalyzed autoxidation. In view of the present work, the observed lack of stoichiometry can be explained by the ability of Asc to undergo PRA leading to the formation of Oxa-Thr. Thus, two outcomes for the reactions of alkylperoxyl radicals with Asc need to be taken into consideration. The trapping of ROO^•^ and their conversion into corresponding ROOH has the potential to be detrimental due to the degradation of hydroperoxides into electrophilic carbonyl compounds and highly reactive radicals such as RO^•^, R^•^, and ROO^•^ that can propagate oxidative damage. On the other hand, the conversion of ROO^•^ into the corresponding ROH by Asc (as evidenced here) circumvents the generation of subsequent reactive radical species, with the trade-off being the irreversible loss of a molecule of Asc. In biological contexts, the latter reaction likely carries less severe consequences.

## 5. Conclusions

The ability of Asc to undergo single electron transfers with ROO^•^ is a widely accepted reaction that underscores its role as a biological antioxidant. We demonstrate a novel pathway of reaction between ROO^•^ and Asc that leads to unconventional products and likely occurs in competition with the electron transfer mechanism. This pathway consists of initial peroxyl radical addition to the enediol portion of Asc, followed by one-electron oxidation of the resulting radical site by O_2_. The intermediate thereby produced undergoes facile Baeyer–Villiger rearrangement that ultimately leads to the conversion of ROO^•^ into the corresponding alcohol product (ROH) and conversion of Asc into the unstable cOxa-Thr. The decomposition of cOxa-Thr affords a series of products that arise from its hydrolysis, including Oxa-Thr regioisomers, together with threonate and oxalate as final decomposition products. Although it was necessary to employ a C6-substiuted derivative of Asc to demonstrate this reaction in the current study, one can expect that similar reactions between Asc and peroxyl radicals take place in cells and tissues. The present findings expand our understanding of the complex chemistry of Asc, which should help investigate its function as a major antioxidant in redox biology.

## Figures and Tables

**Table 1:** (−)ESI-TOF HRMS analysis of compounds [M−H]^−^.

Compd	Calcd for	Calcd	Found	Δ ppm
Asc-PA	C_14_H_14_O_7_	293.0667	293.0675	2.7
DHA-PA	C_14_H_12_O_5_	291.0510	291.0515	1.7
cOxa-Thr-PA	C_14_H_12_O_8_	307.0459	307.0458	0.3
Oxa-Thr-PA	C_14_H_14_O_9_	325.0565	325.0567	0.6
Thr-PA	C_12_H_14_O_6_	253.0718	253.0720	0.8

**Table 2:** Summary of ^1^H and ^13^C-NMR analyses.

**^1^H-NMR**	**Asc-PA** **(Ace)**	**cOxa-Thr-PA** **(Ace)**	**Oxa-Thr-PA** **(Ace)**	**Oxa-Thr-PA** **(Major/Minor)** **(D_2_O)**	**Thr-PA** **(DMSO)**
H4	4.72 (d)	5.66 (d)	5.30 (d)	5.23 (d)/4.56	3.56 (dd)
H5	4.24–4.29 (m)	5.37 (ddd)	4.52 (dd)	4.49 (m)/5.59	3.81 (m)
H6a	4.12–4.21 (m)	4.84 (dd)	4.24 (dd)	4.30 (m)/4.45	3.88 (m)
H6b	4.12–4.21 (m)	4.64 (dd)	4.31 (dd)	4.23 (m)/4.43	4.03 (dd)
PhCH_2_	3.68 (s)	3.65 (s)	3.69 (s)	3.77 (s)	3.66 (s)
Ar-H	7.19–7.30 (m)	7.22–7.34 (m)	7.22–7.34 (m)	7.28–7.44 (m)	7.24–7.35 (m)
**^13^C-NMR**	**Asc-PA** **(Ace)**	**cOxa-Thr-PA** **(Ace)**	**Oxa-Thr-PA** **(Ace)**	**Oxa-Thr-PA** **(Major/Minor)** **(D_2_O)**	**Thr-PA** **(DMSO)**
C1	170.3	152.5	158.4	161.3/161.2	---
C2	120.0	153.2	158.6	161.7/161.2	---
C3	151.1	169.2	168.1	170.7/174.0	174.6
C4	76.1	76.0	75.3	74.2/73.4	70.1
C5	67.5	76.0	69.1	67.9/69.2	69.6
C6	65.8	64.0	64.9	64.3/62.9	66.3
PhCH_2_	41.3	41.0	41.3	40.3/40.5	40.2
PhCH_2_CO	171.7	171.1	171.6	174.2/174.0	171.3
Ar-C	127.7, 129.2, 130.2, 135.4	127.9, 129.3, 130.3, 134.7	127.7, 129.2, 130.2, 134.3	127.5, 128.9, 129.4, 133.8	126.7, 128.3, 129.4, 134.5

## Data Availability

Data is contained within the article and supplementary material.

## Supplementary Materials

The following supporting information can be downloaded at: https://www.mdpi.com/article/10.3390/antiox13101194/s1, MS/MS transitions used for MRM analyses, MS, and NMR spectra of phenylacetate derivatives, textual interpretation of NMR spectra, identification of compounds via EDC coupling, oxidation of Asc-PA by ABAP and AIBN.
