# Supplementary material for: Scavenging of Alkylperoxyl Radicals by Addition to Ascorbate: An Alternative Mechanism to Electron Transfer
Source: Antioxidants (Basel). 2024 Oct 1;13(10):1194. doi: 10.3390/antiox13101194 (PMC11504153; doi:10.3390/antiox13101194)
Supplement: Supplementary file 1 [file antioxidants-13-01194-s001.zip › antioxidants-3221873-supplementary.pdf]

Supporting Information

Scavenging of alkylperoxyl radicals by ascorbate: an alternative mechanism to electron transfer

Gabriel Robert[1] and J Richard Wagner[2]*

[1]Department of Biochemistry, [2]Department of Nuclear Medicine and Radiobiology, Faculty of Medicine and Health Sciences, Université de Sherbrooke, 3001, 12e Avenue Nord, Sherbrooke, Québec, J1H 5N4, Canada

*Correspondence: richard.wagner@usherbrooke.ca

Table of Contents

MS/MS transitions used for MRM analyses S3

MS characterization of phenylacetate derivatives S4-S19

- Asc-PA MS characterization S4
- DHA-PA MS characterization S6
- Thr-PA MS characterization S10
- cOxa-Thr-PA MS characterization S12
- Oxa-Thr-PA MS characterization S14
- MS/MS fragmentation of phenylacetate derivative S18

NMR characterization of phenylacetate derivatives S20-S45

- Asc-PA NMR characterization S20
- Thr-PA NMR characterization S23
- cOxa-Thr-PA NMR characterization S28
- Oxa-Thr-PA NMR characterization S36
- NMR simulations of phenylacetate derivatives S45

EDC-mediated coupling of Oxa-Thr-PA with 2-phenylethanamine S46

Oxidation of Asc-PA by peroxyl radicals derived from azo compounds S54

MS/MS transitions used for MRM analyses

Table S1. MS/MS transitions of thymidine derivatives (positive mode)

ID	(+)Q1 Mass (Da)	(+)Q3 Mass (Da)	CE (Volts)
dU	229.000	113.000	30.000
dT	243.200	127.000	14.000
hmdU	259.020	125.000	23.500
hpmdU	275.200	159.200	10.000
fdU	256.960	140.980	14.500
cadU	273.200	157.200	14.000

Table S2. MS/MS transitions of phenylacetate derivatives (negative mode)

ID	(-)Q1 Mass (Da)	(-)Q3 Mass (Da)	CE (Volts)
Asc-PA	293.000	135.100	-16.000
Asc-PA	292.900	174.800	-19.000
DHA-PA	290.800	172.900	-19.000
cOxa-Thr-PA	306.800	135.000	-13.000
Oxa-Thr-PA	325.000	135.000	-21.000
Oxa-Thr-PA	325.000	252.000	-11.000
Thr-PA	253.000	91.000	-25.000
Thr-PA	253.000	135.000	-15.000
PA	135.000	91.000	-12.000

Asc-PA MS characterization

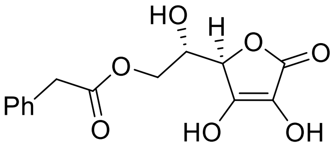

Exact Mass: 294.0740

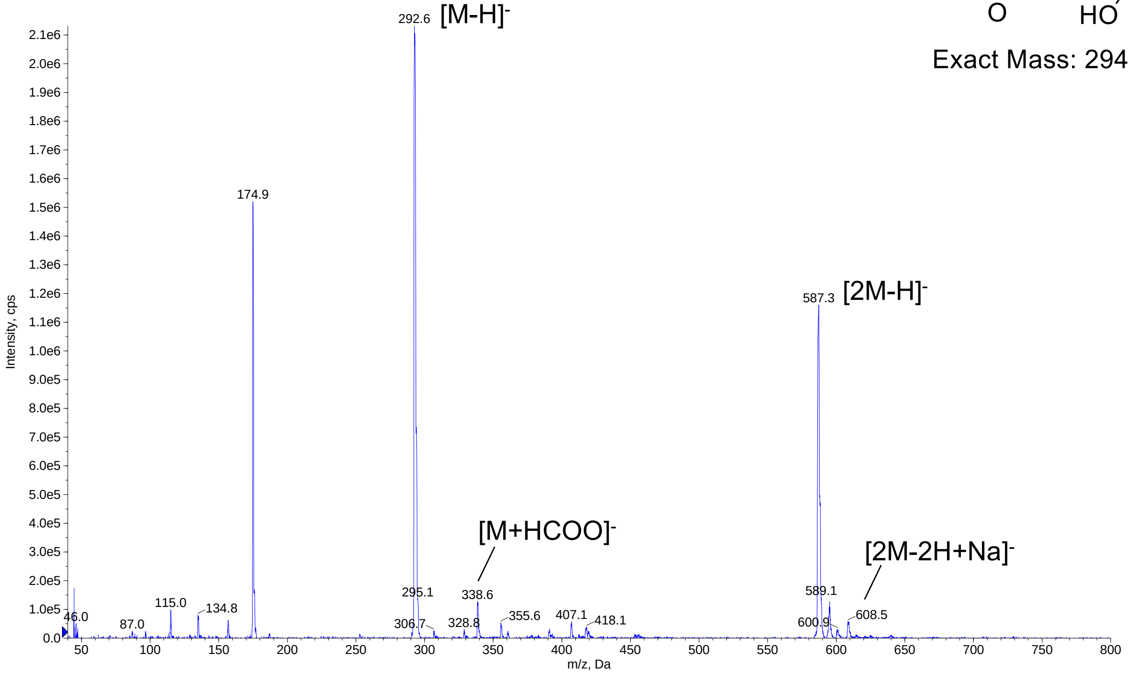

Figure S1. (-)Q1 MS spectrum of Asc-PA.

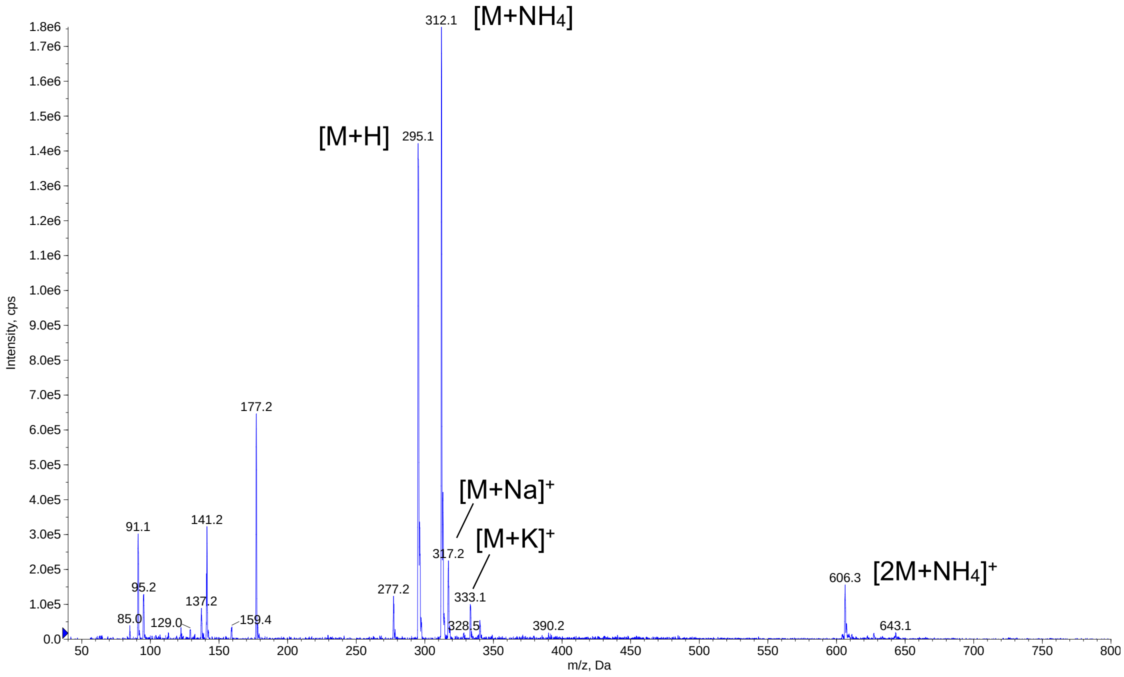

Figure S2. (+)Q1 MS spectrum of Asc-PA.

Asc-PA MS characterization (continued)

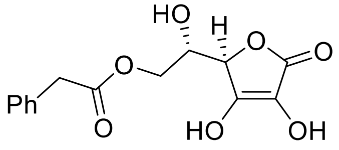

Exact Mass: 294.0740

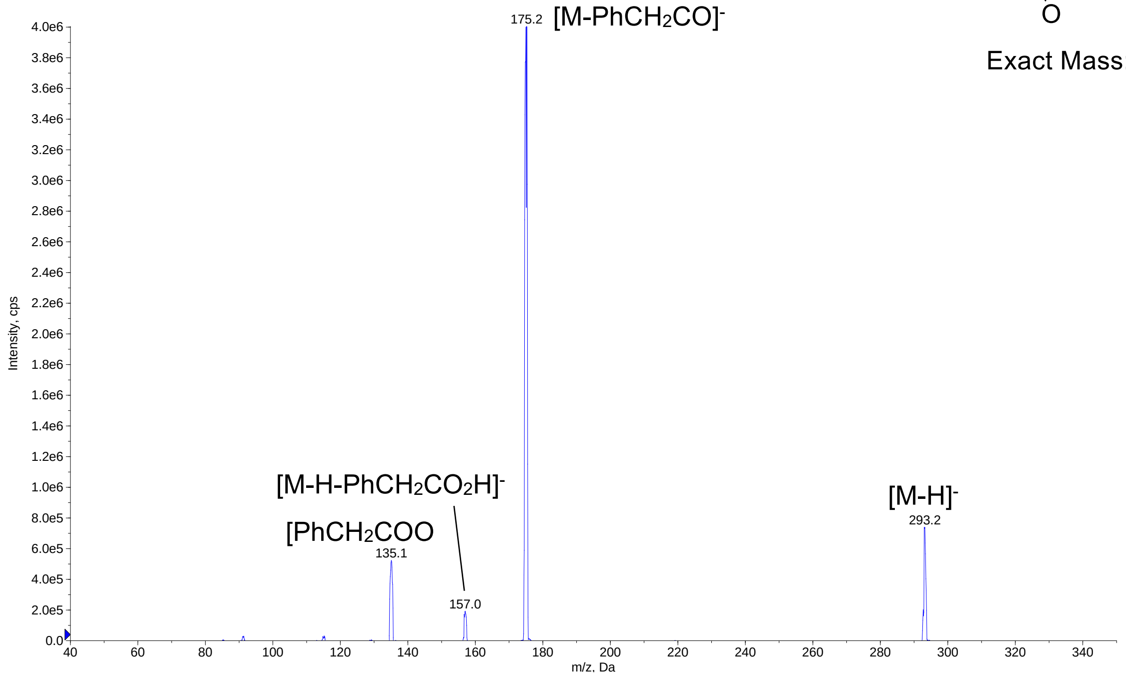

Figure S3. (-)293 MS/MS spectrum of Asc-PA.

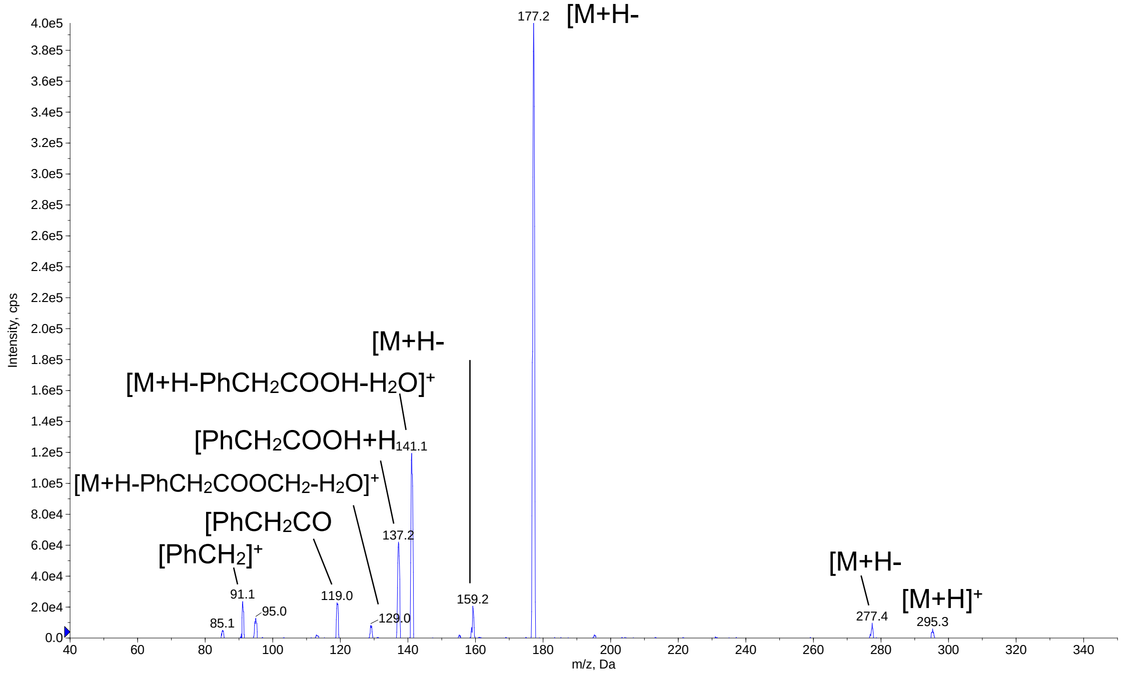

Figure S4. (+)295 MS/MS spectrum of Asc-PA.

DHA-PA MS characterization

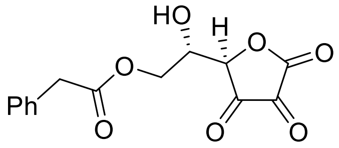

Exact Mass: 292.0583

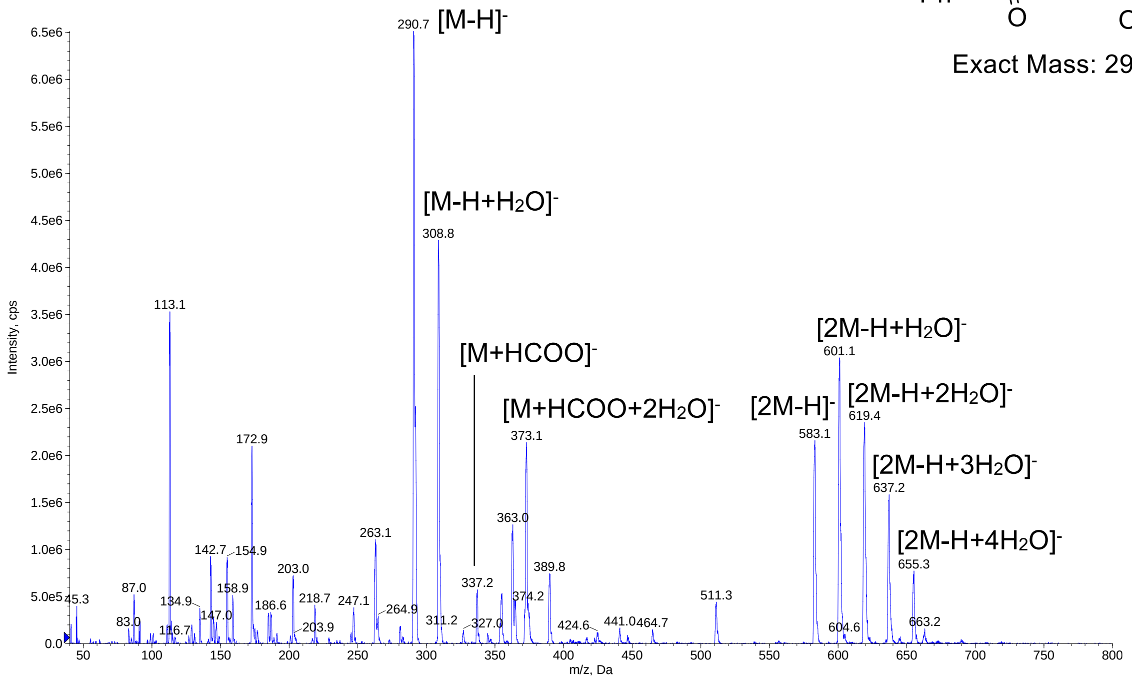

Figure S5. (-)Q1 MS spectrum of DHA-PA.

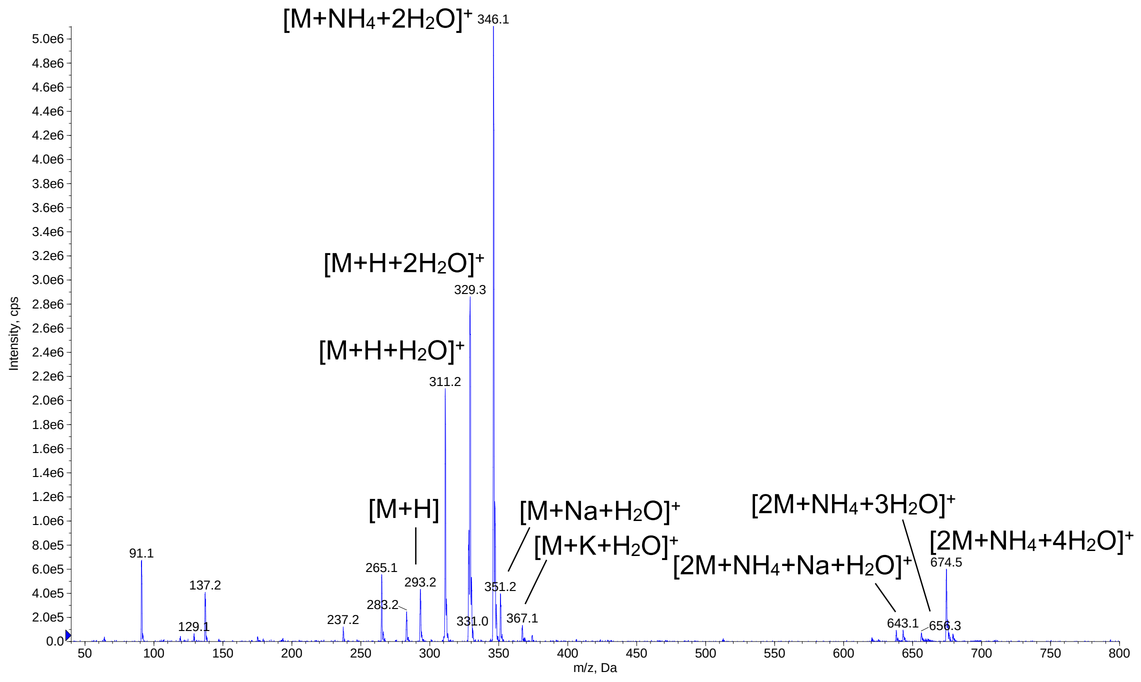

Figure S6. (+)Q1 MS spectrum of DHA-PA.

DHA-PA MS characterization (continued)

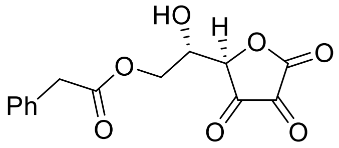

Exact Mass: 292.0583

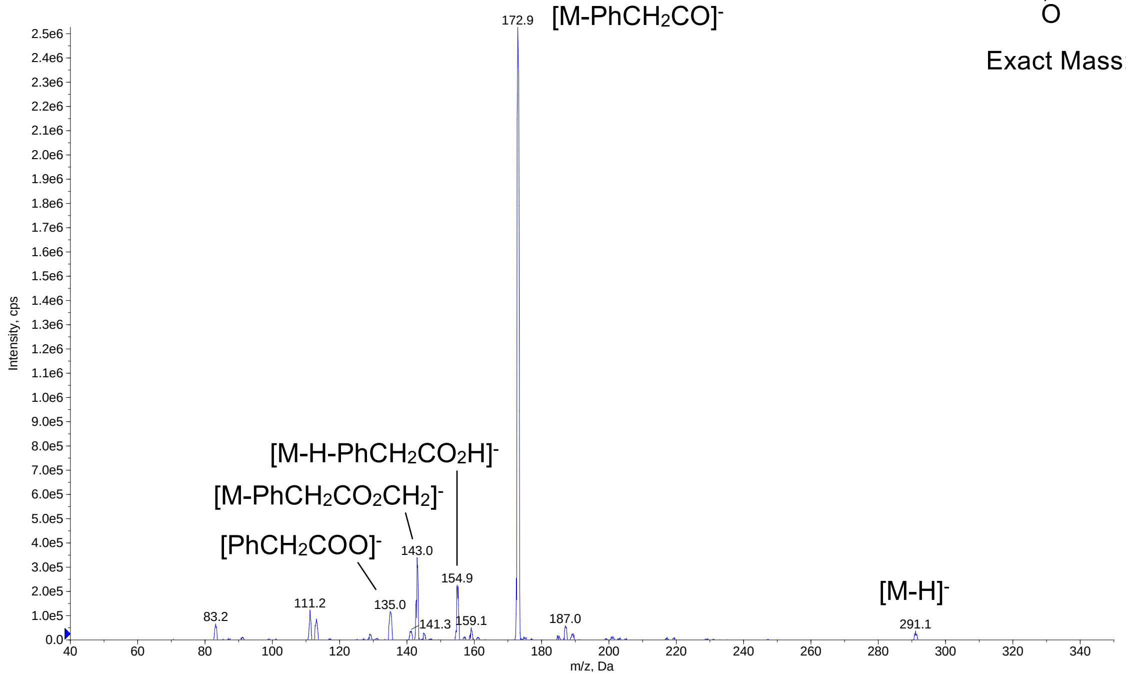

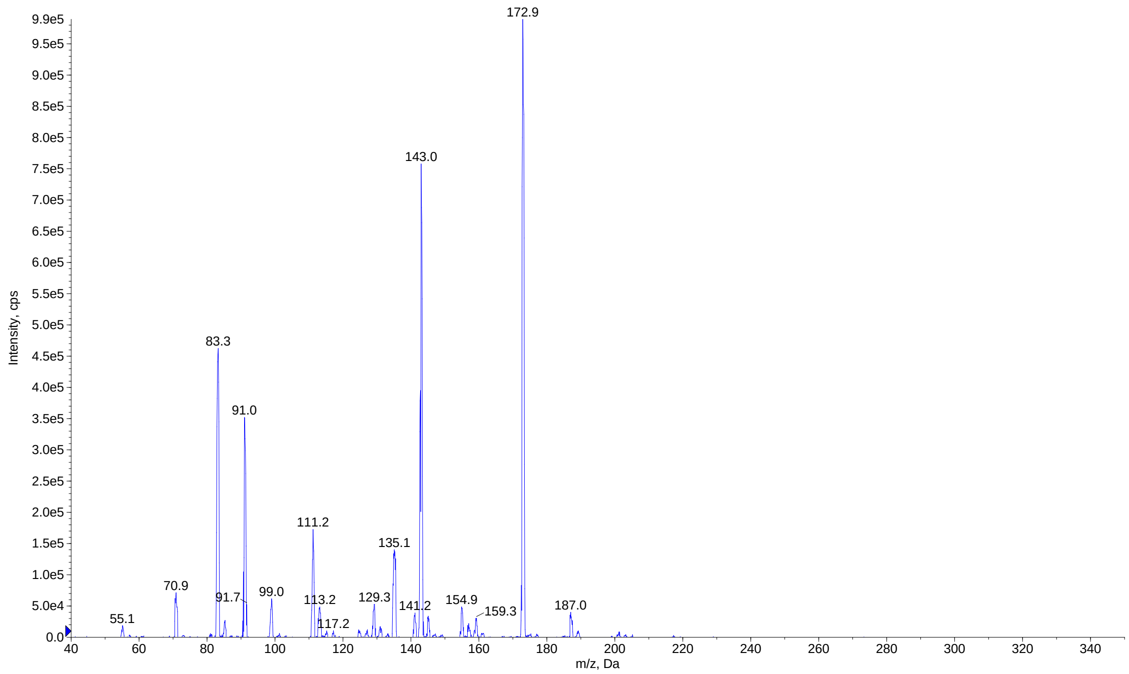

Higher collision energy

Figure S7. (-)291 MS/MS of DHA-PA.

DHA-PA MS characterization (continued)

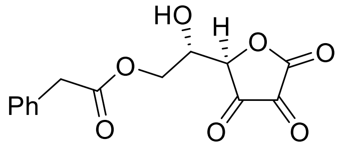

Exact Mass: 292.0583

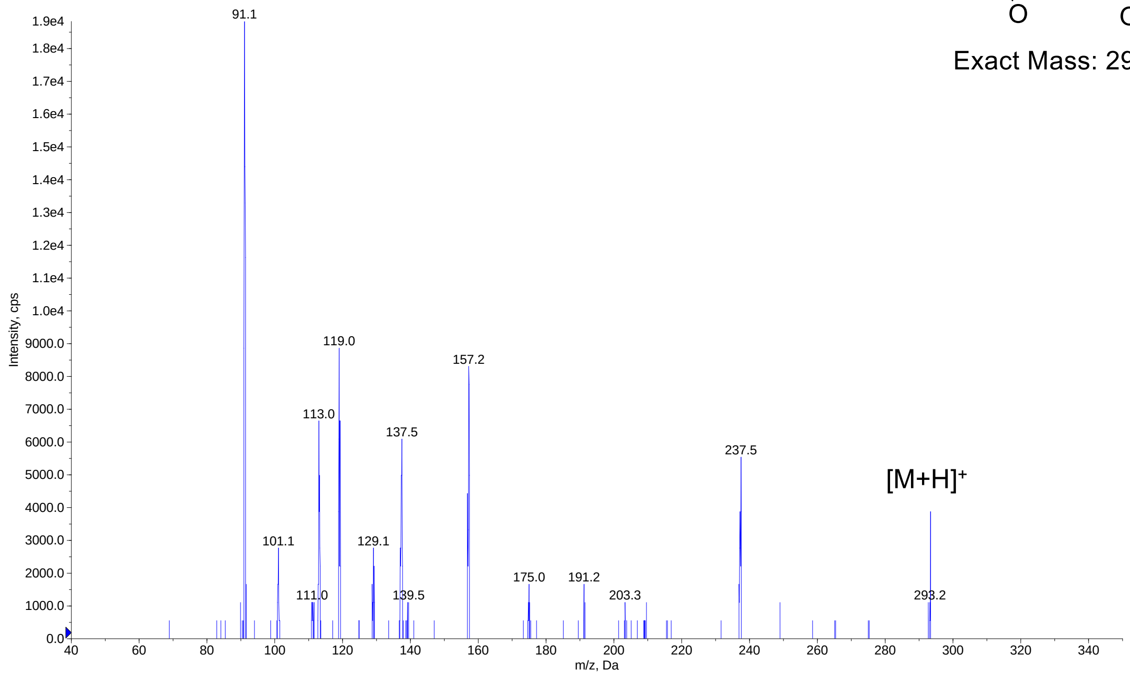

Figure S8. (+)293 MS/MS spectrum of DHA-PA.

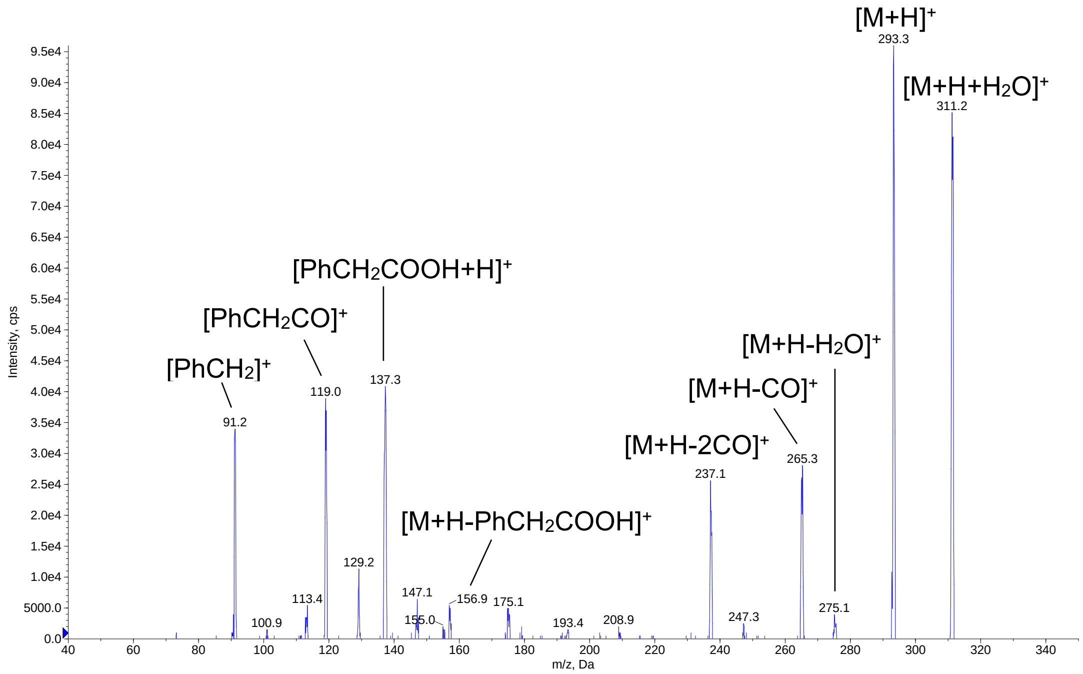

Figure S9. (+)311 MS/MS spectrum of DHA-PA.

DHA-PA MS characterization (continued)

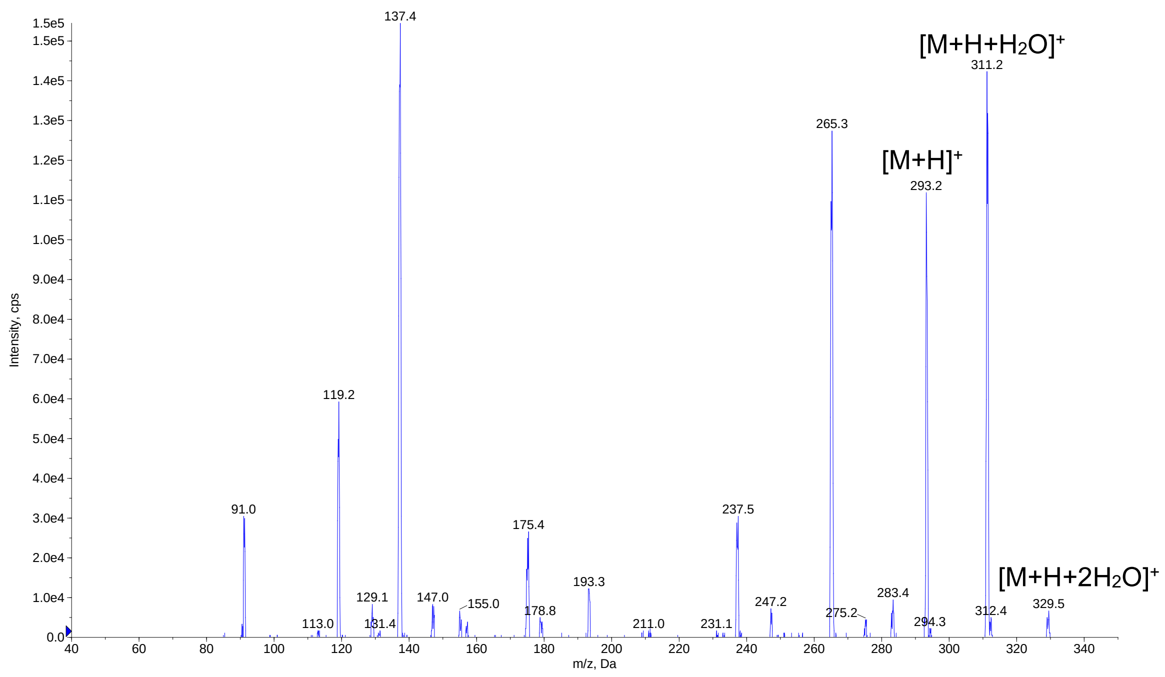

Figure S10. (+)329 MS/MS spectrum of DHA-PA

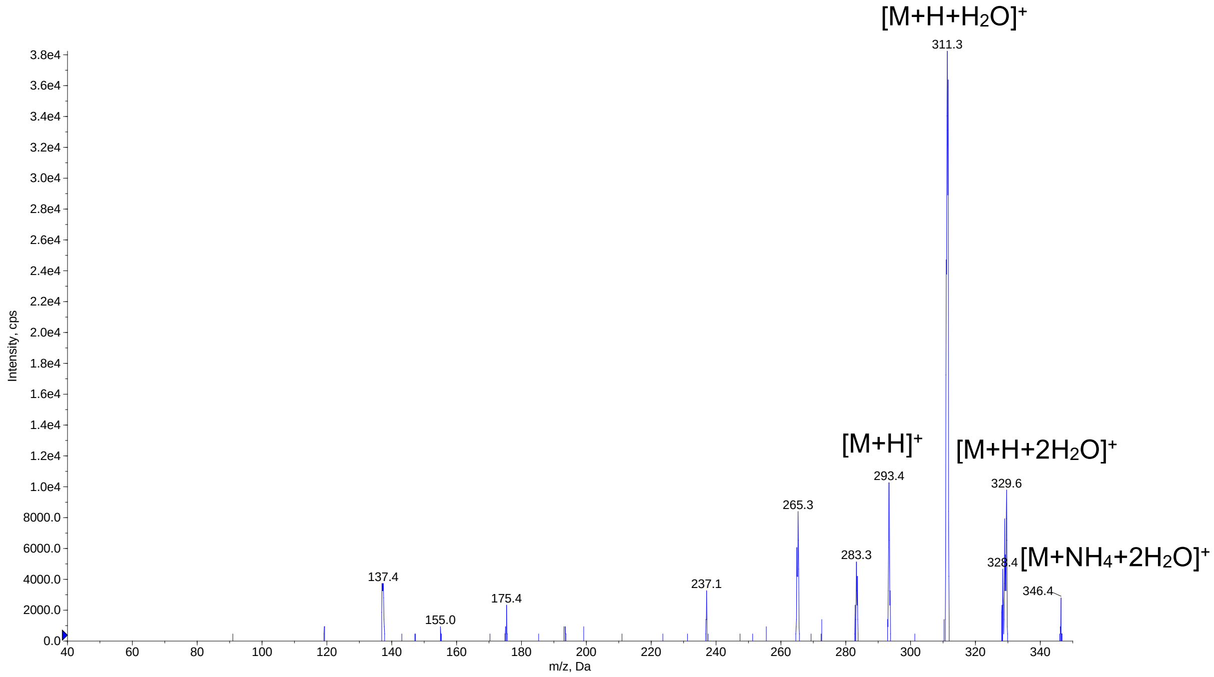

Figure S11. (+)346 MS/MS spectrum of DHA-PA.

Thr-PA MS characterization

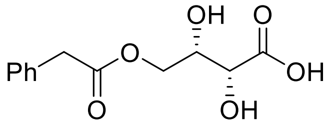

Exact Mass: 254.0790

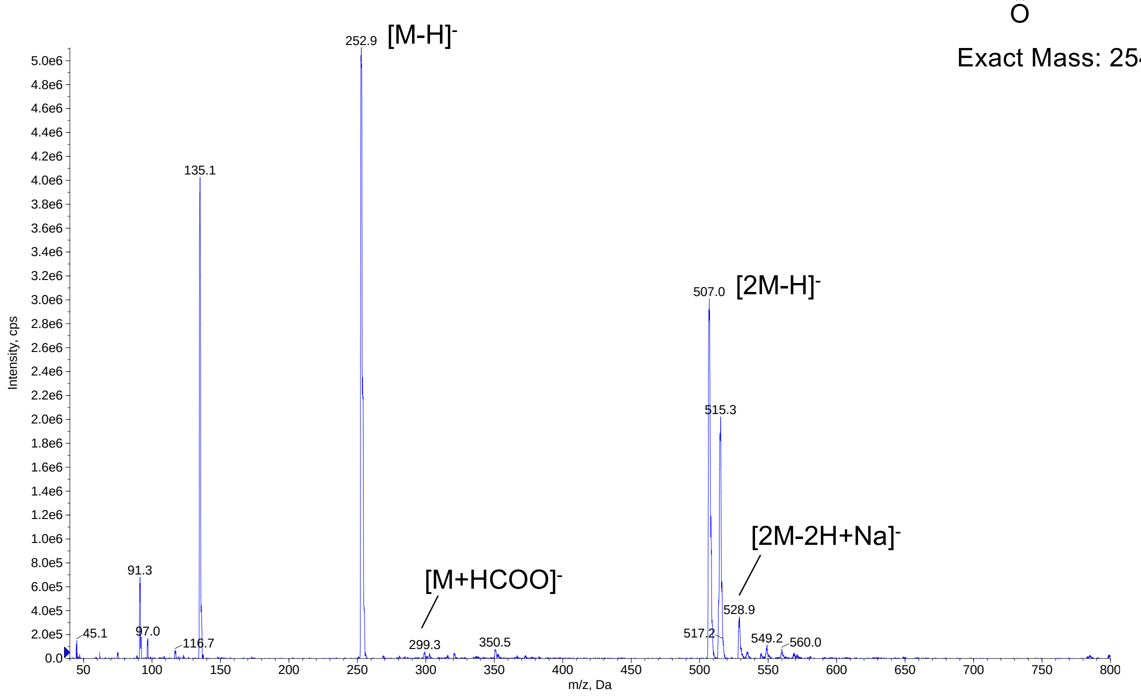

Figure S12. (-)Q1 MS spectrum of Thr-PA.

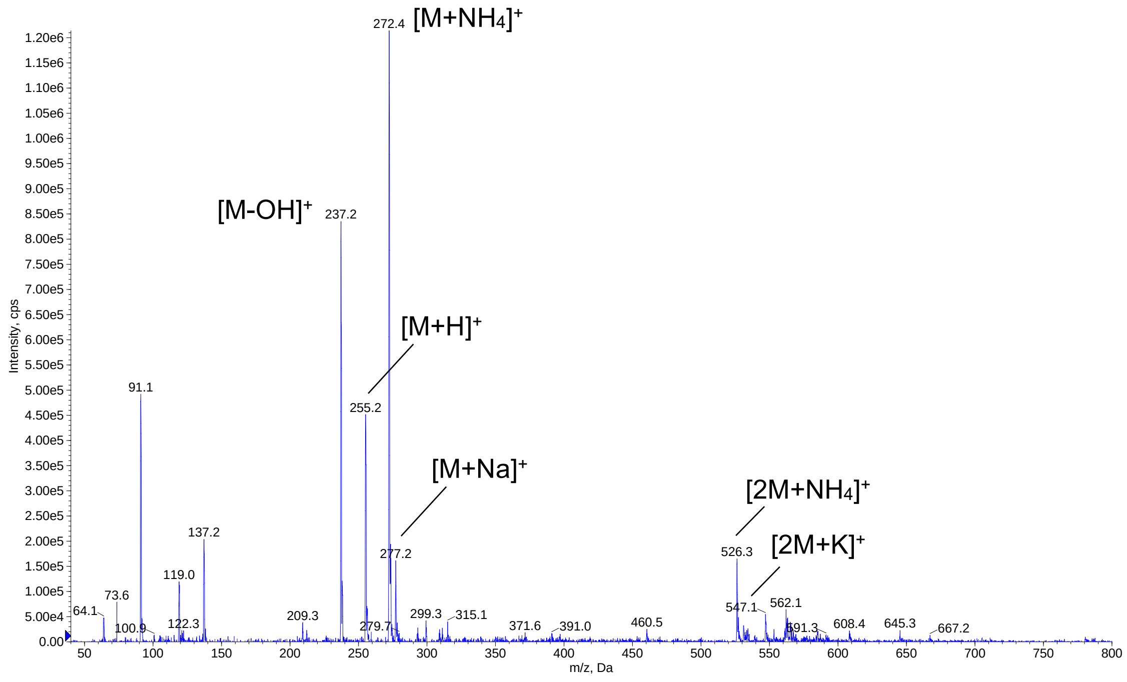

Figure S13. (+)Q1 MS spectrum of Thr-PA.

Thr-PA MS characterization (continued)

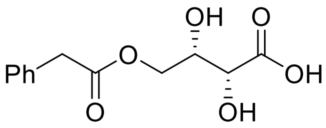

Exact Mass: 254.0790

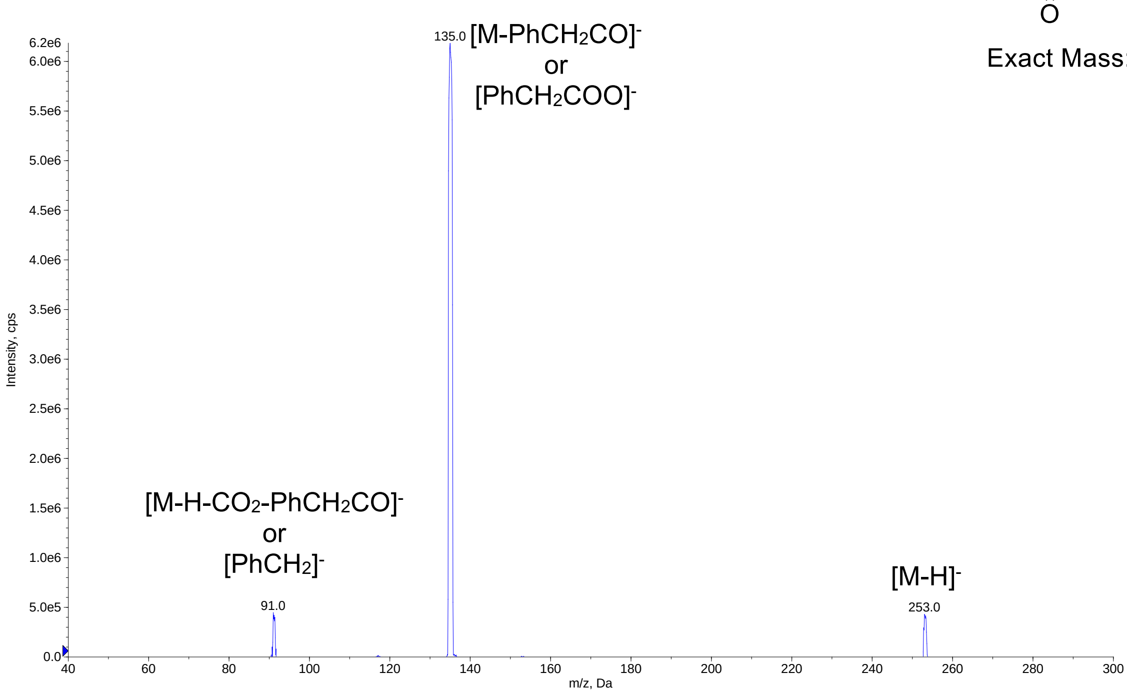

Figure S14. (-)253 MS/MS spectrum of Thr-PA.

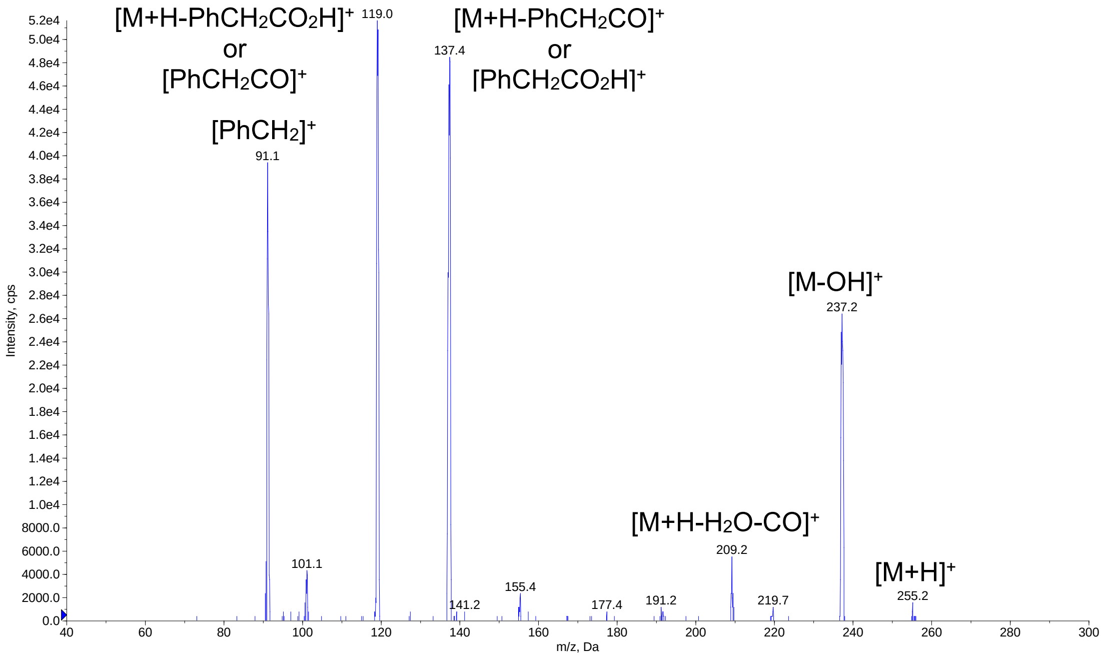

Figure S15. (+)255 MS/MS spectrum of Thr-PA.

cOxa-Thr-PA MS characterization

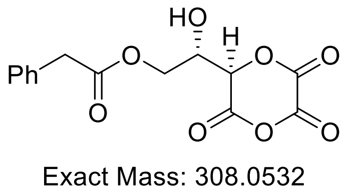

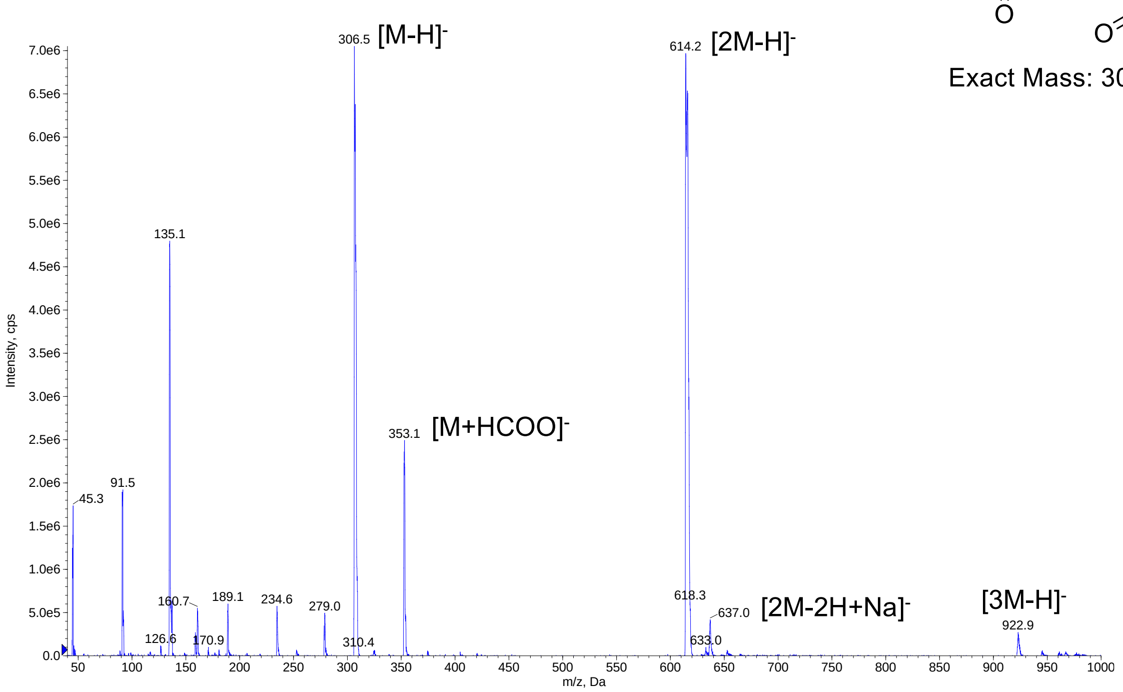

Figure S16. (-)Q1 MS spectrum of cOxa-Thr-PA.

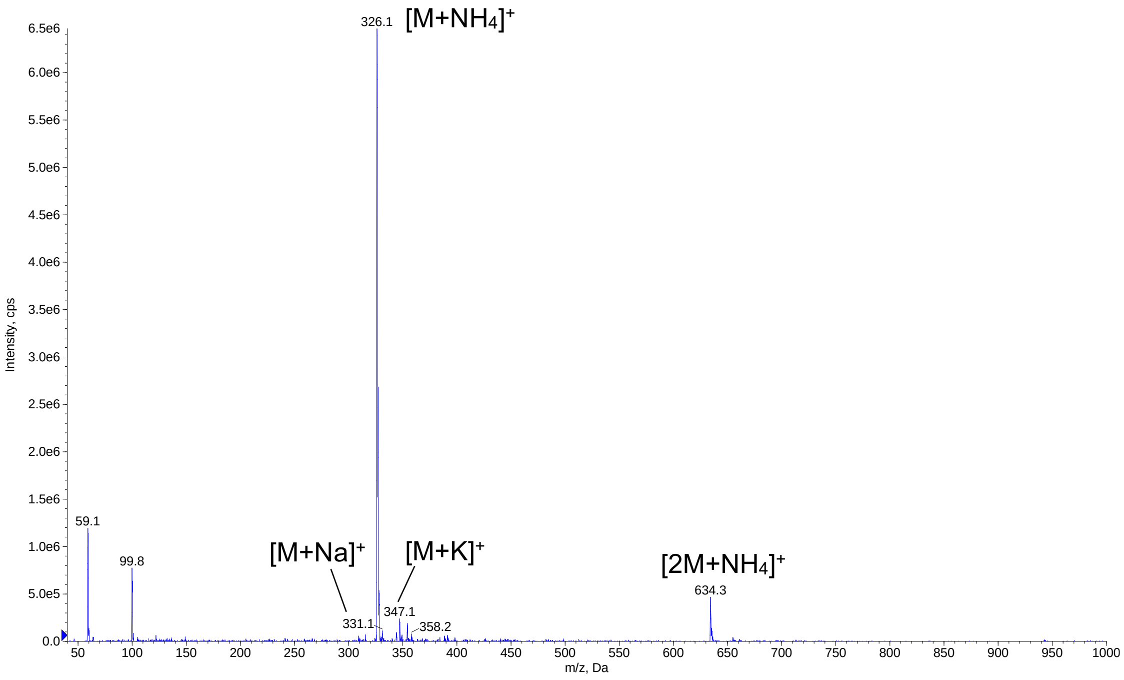

Figure S17. (+)Q1 MS spectrum of cOxa-Thr-PA.

cOxa-Thr-PA characterization MS (continued)

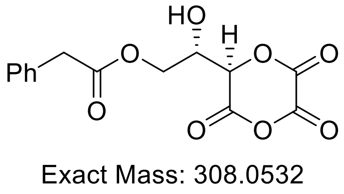

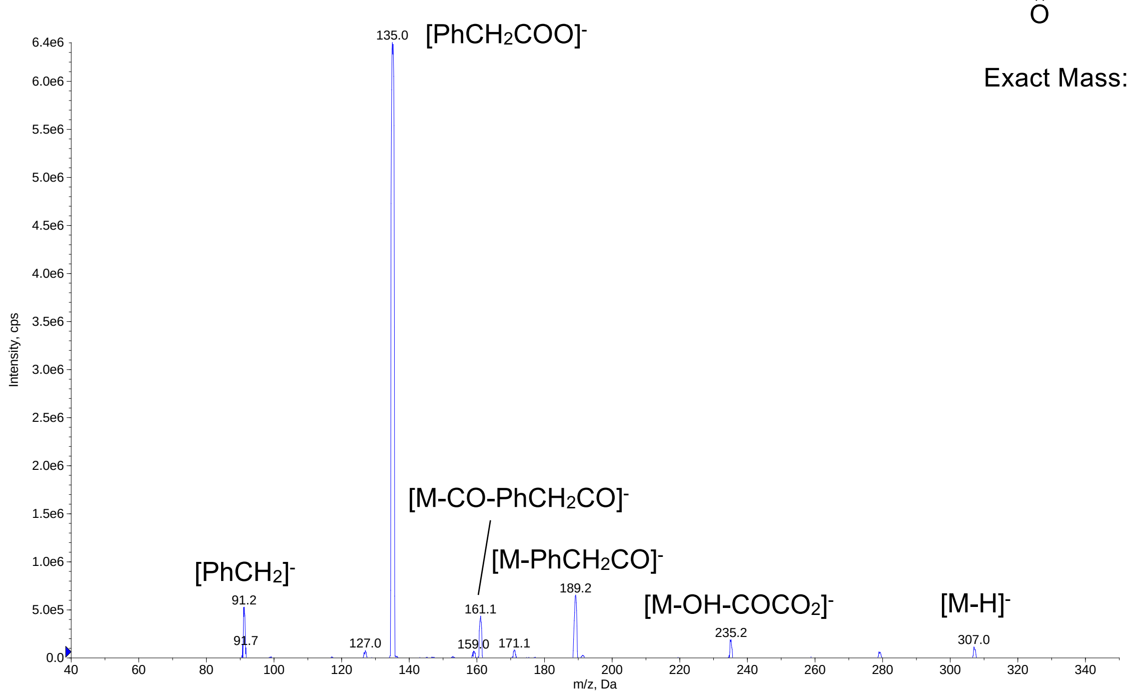

Figure S18. (-)307 MS/MS spectrum of cOxa-Thr-PA.

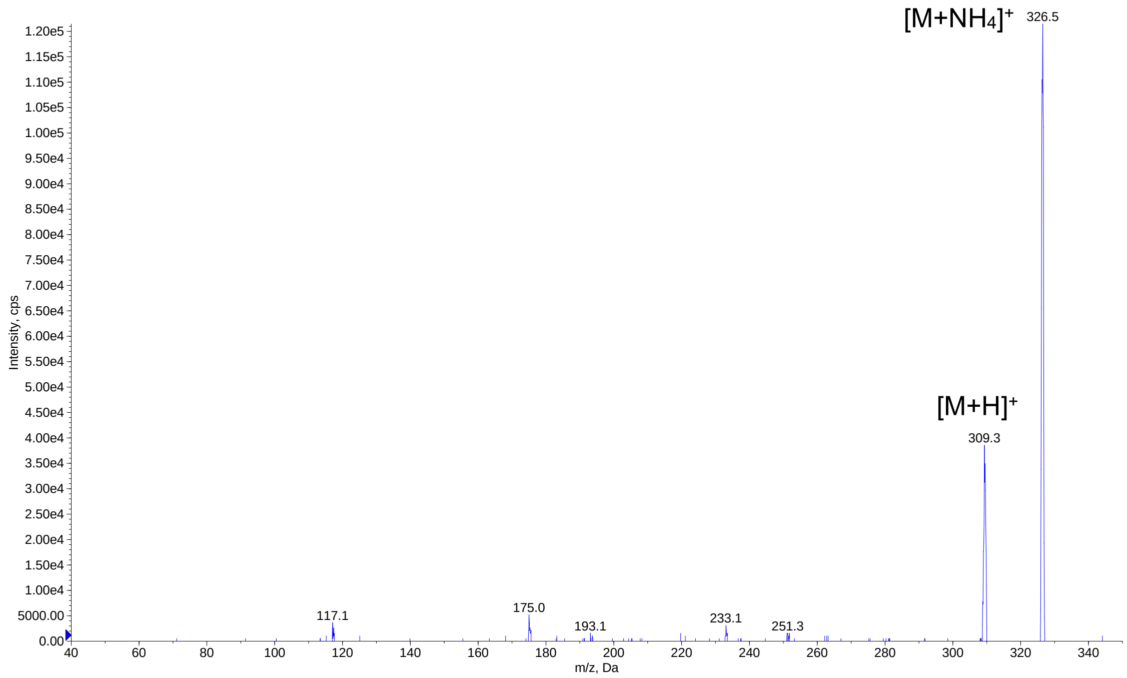

Figure S19. (+)326 MS/MS spectrum of cOxa-Thr-PA.

Oxa-Thr-PA MS characterization

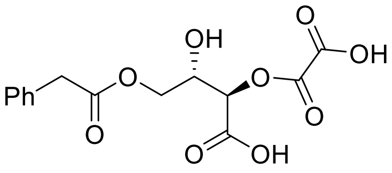

Exact Mass: 326.0638

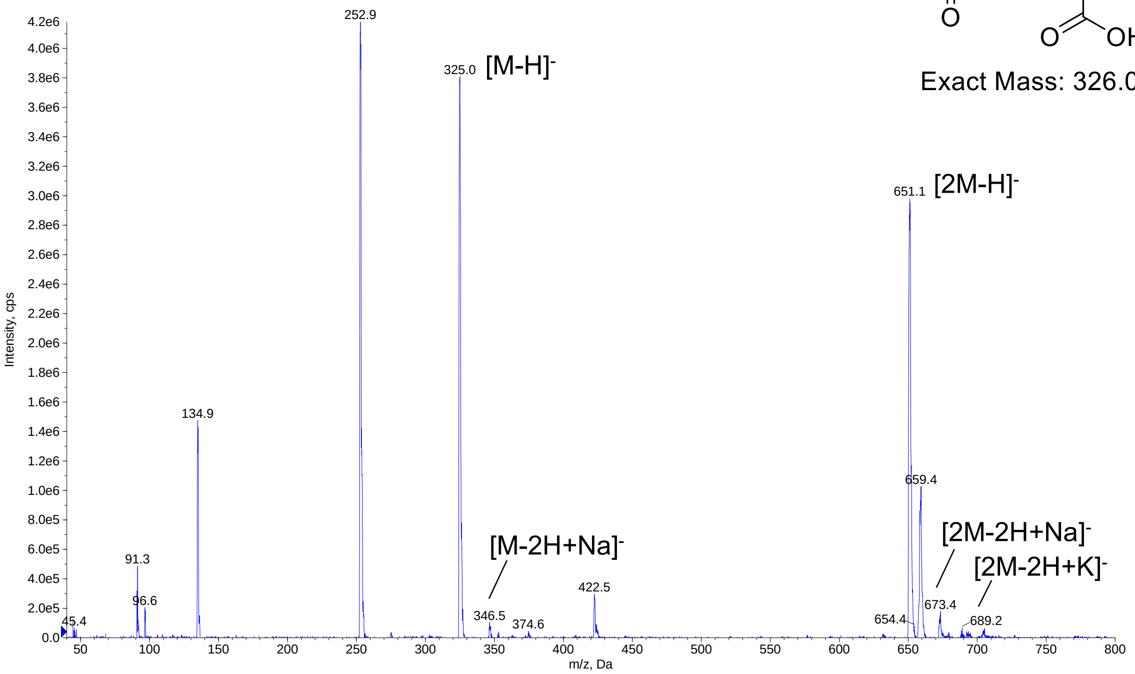

Figure S20. (-)Q1 MS spectrum of Oxa-Thr-PA.

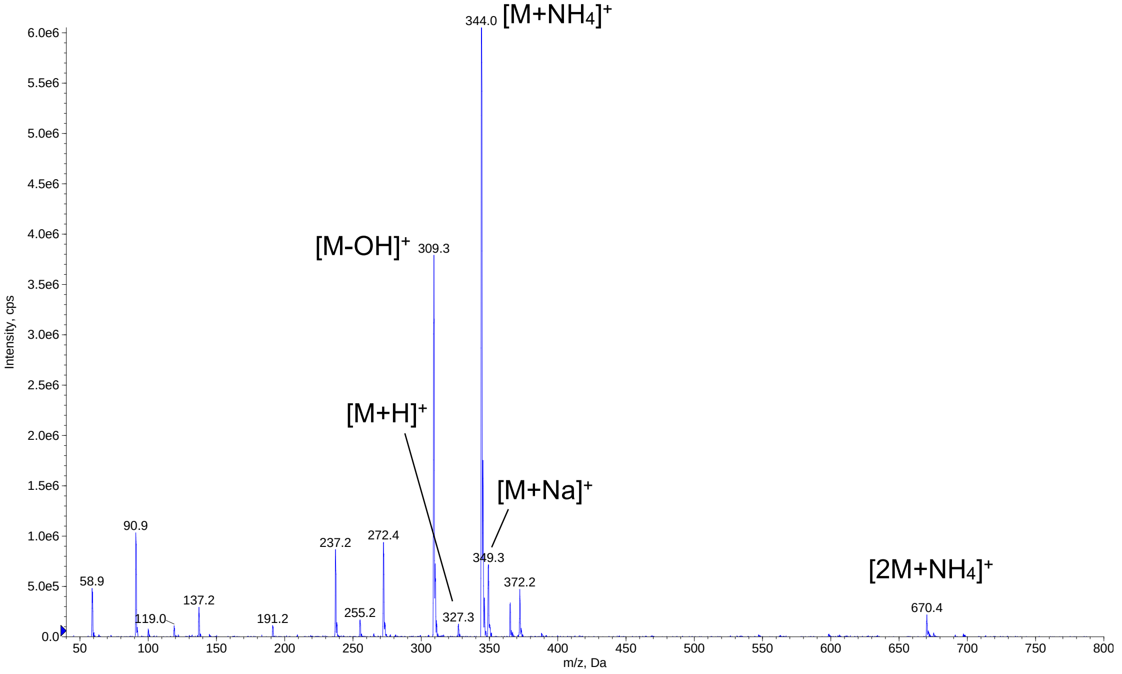

Figure S21. (+)Q1 MS spectrum of Oxa-Thr-PA.

Oxa-Thr-PA MS characterization (continued)

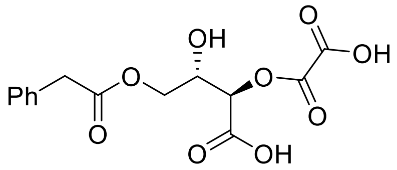

Exact Mass: 326.0638

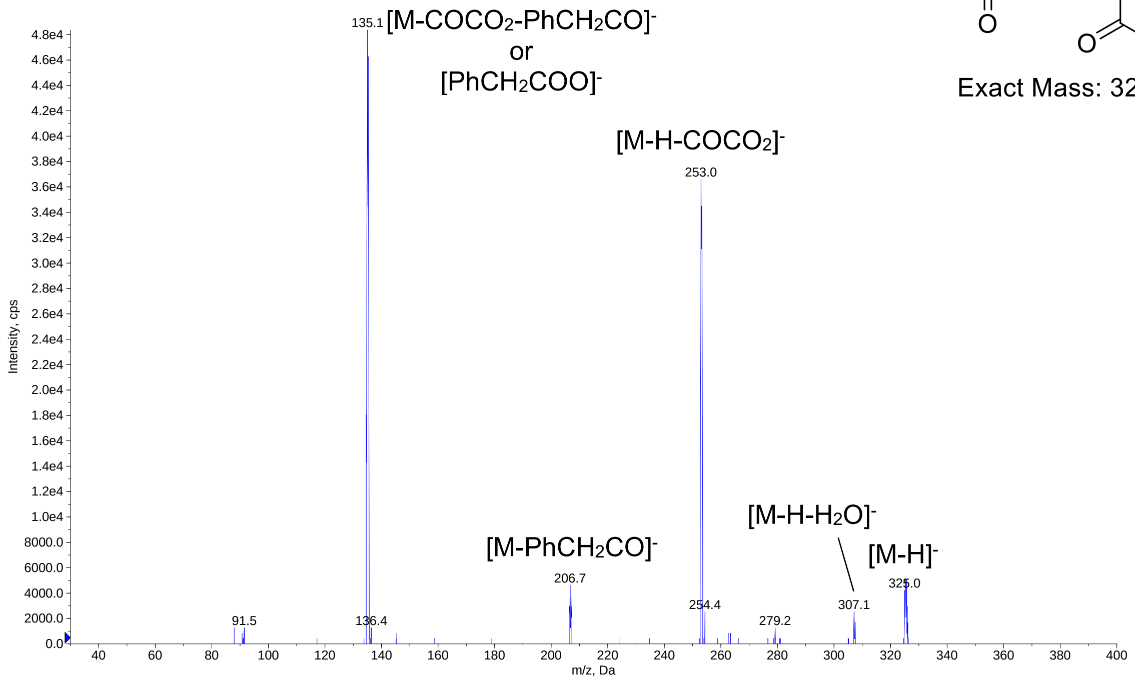

Figure S22. (-)325 MS/MS spectrum of Oxa-Thr-PA.

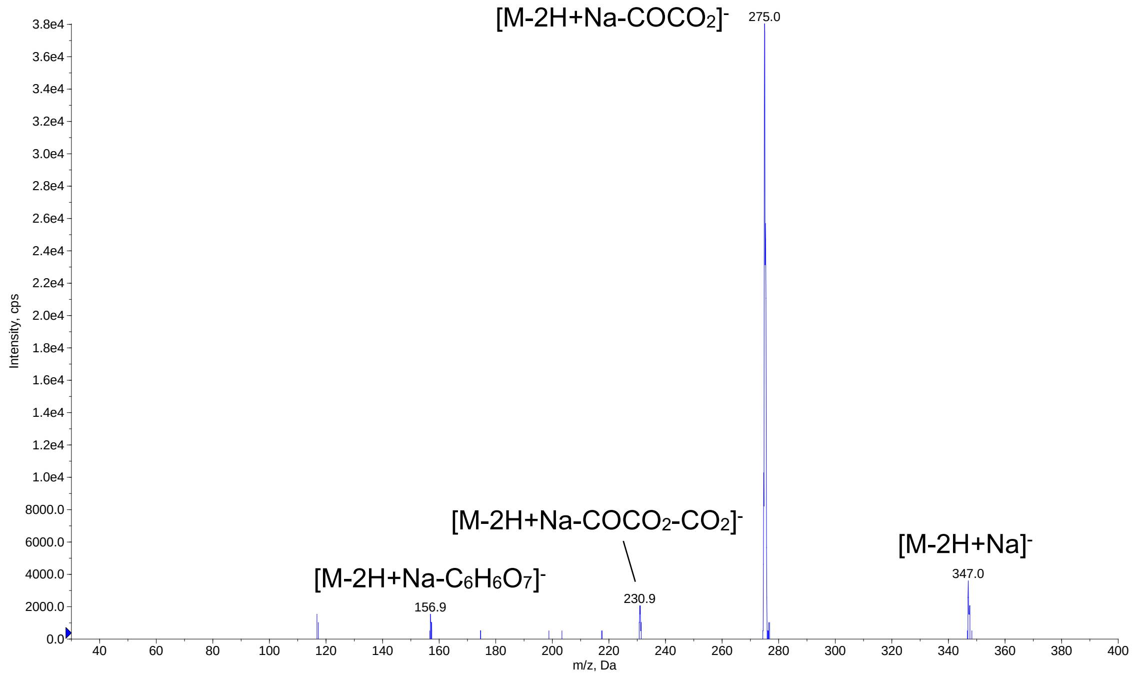

Figure S23. (-)347 MS/MS spectrum of Oxa-Thr-PA.

Oxa-Thr-PA MS characterization (continued)

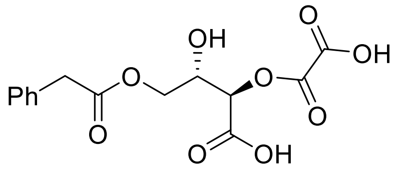

Exact Mass: 326.0638

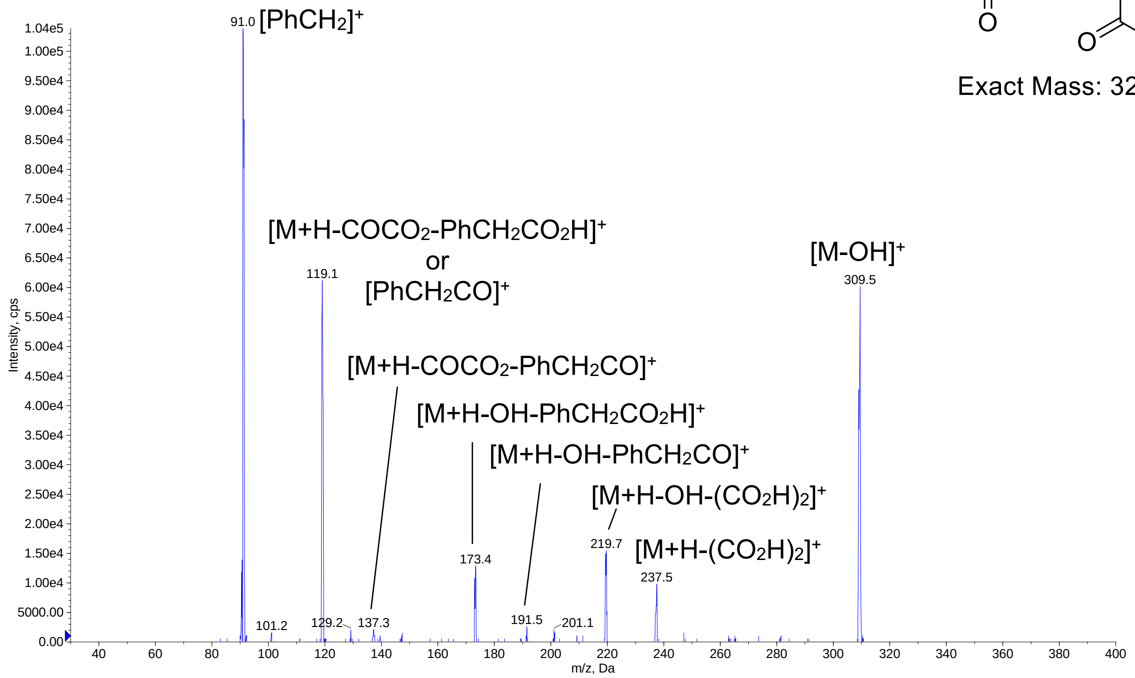

Figure S24. (+)327 MS/MS spectrum of Oxa-Thr-PA.

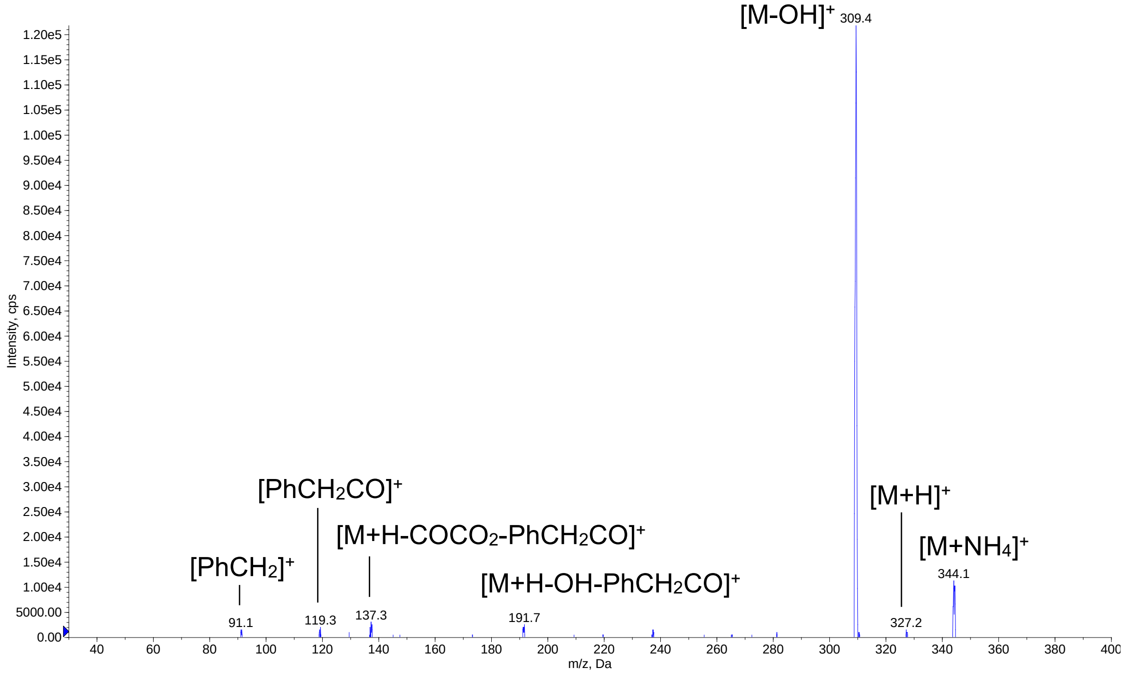

Figure S25. (+)344 MS/MS spectrum of Oxa-Thr-PA.

Oxa-Thr-PA MS characterization (continued)

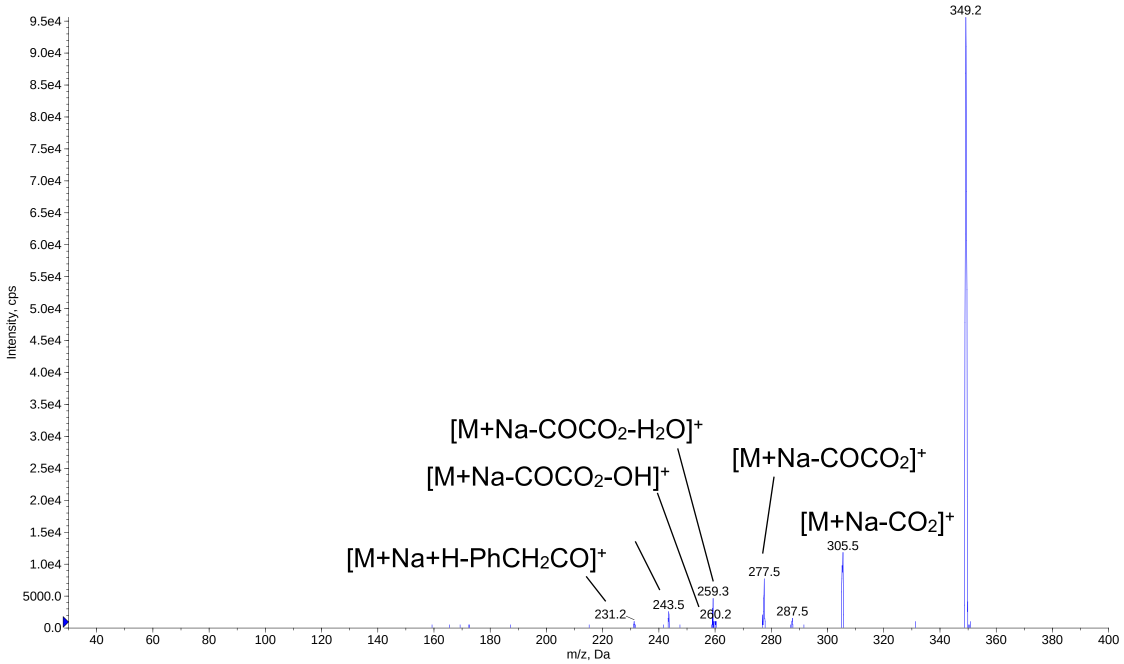

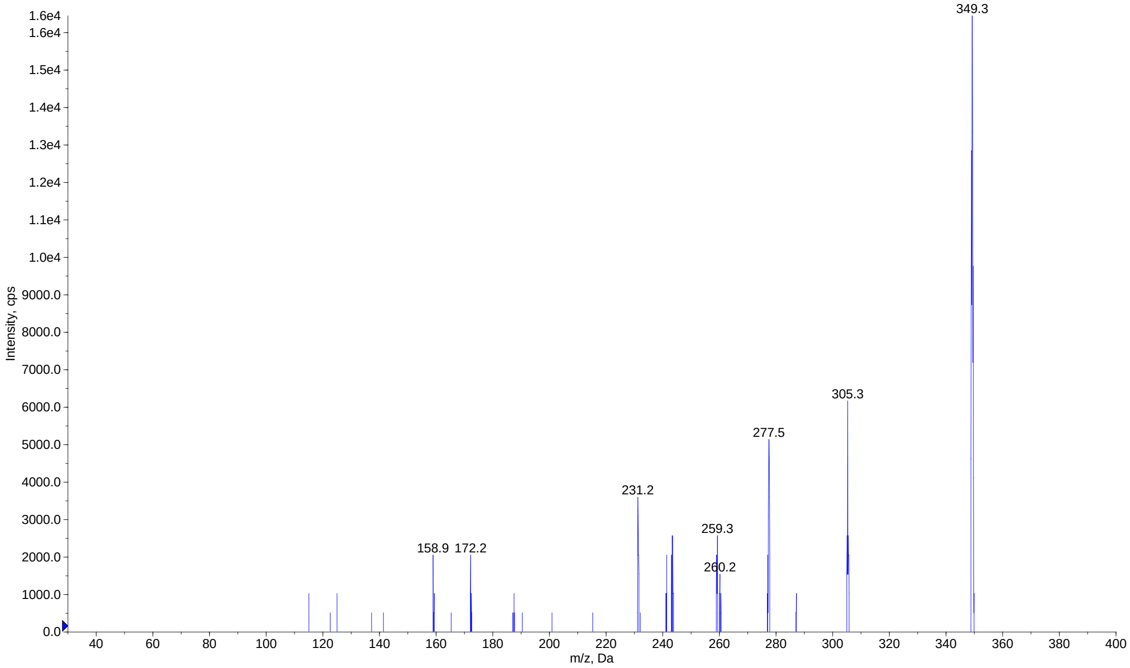

↯ *Higher collision energy*

Figure S26. (+)349 MS/MS spectrum of Oxa-Thr-PA.

MS/MS fragmentation of phenylacetate derivative

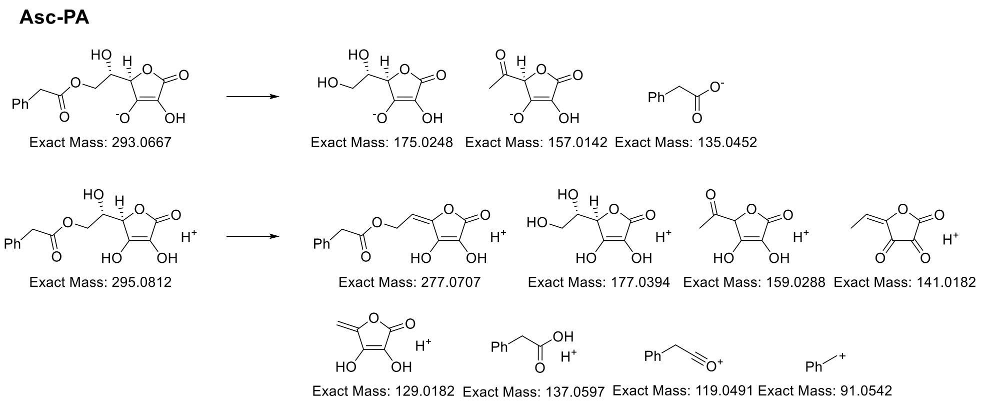

Figure S27. (-)MS/MS and (+)MS/MS fragmentation of Asc-PA.

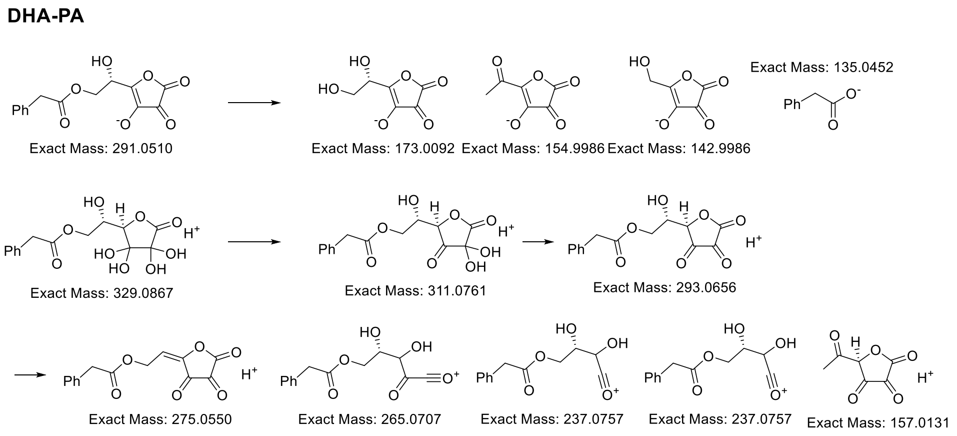

Figure S28. (-)MS/MS and (+)MS/MS fragmentation of DHA-PA.

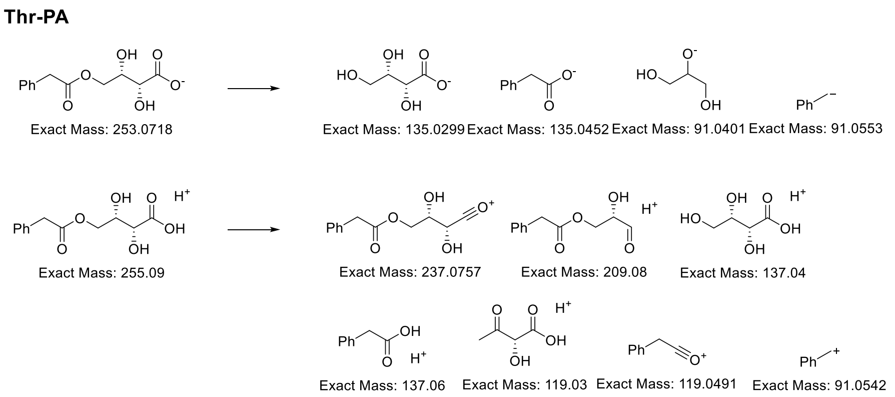

Figure S29. (-)MS/MS and (+)MS/MS fragmentation of Thr-PA.

MS/MS fragmentation of phenylacetate derivatives (continued)

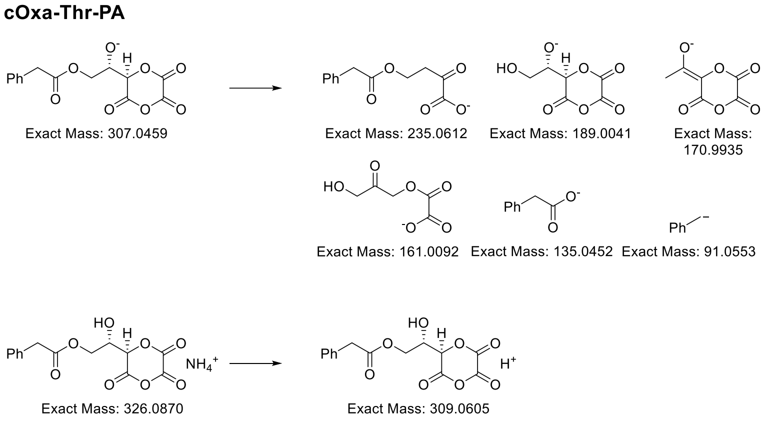

Figure S30. (-)MS/MS and (+)MS/MS fragmentation of cOxa-Thr-PA.

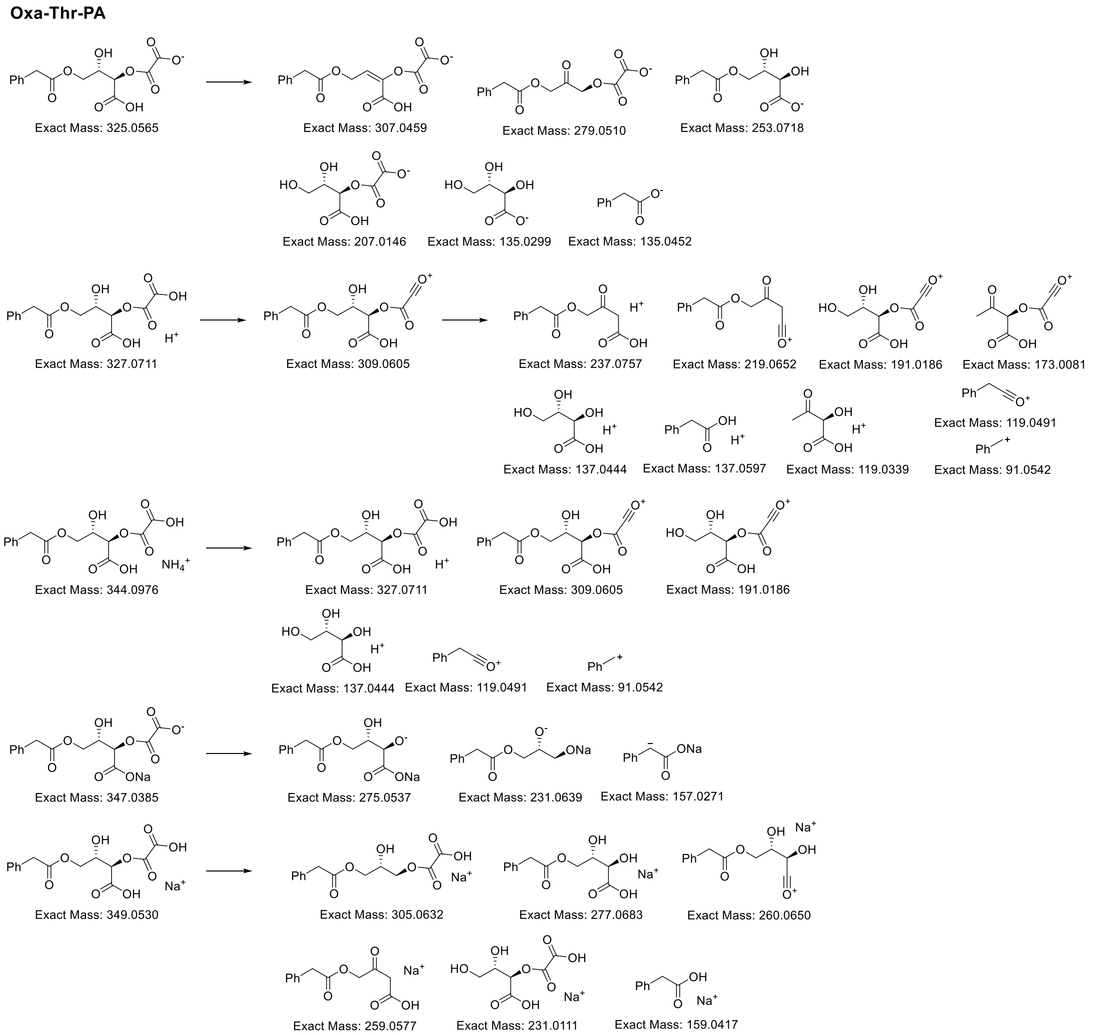

Figure S31. (-)MS/MS and (+)MS/MS fragmentation of Oxa-Thr-PA.

Asc-PA NMR characterization

The ^{1}H-NMR spectrum of Asc-PA in $(CD_3)CO$ (Figure S32) showed the signals of non-exchangeable protons that represent the -CH_2-CH(OH)-CH- backbone of Asc with partial overlap of the H5 and H6 signals (4.12-4.29 ppm) such that H5 was coupled to both H6 and H4 while both protons at H6 were coupled to H5. In contrast, H4 gave a doublet with coupling to H5. The other protons included CH_2 from the phenylacetate group (3.68 ppm) and five protons associated with the phenyl substituent. The ^{1}H spectrum in DMSO showed three additional protons that can be assigned to the OH groups of Asc, which appeared at 11.13, 8.41 and 5.36 ppm. The ^{13}C-NMR spectrum (Figure S33) displayed the different segments of the molecule with signals from phenylacetate substituent, which included $PhCH_2CO$ (171.7 ppm), six aromatic carbons (127.7-135.4 ppm), and the presence of an isolated methylene group, Ph-CH_2 (41.3 ppm). The other signals in the region between 60-80 ppm can be assigned to C4, C5 and C6 while the associated carbonyl and enol carbon atoms can be assigned to the other signals in the region between 120-180 ppm.

Asc-PA NMR characterization (continued)

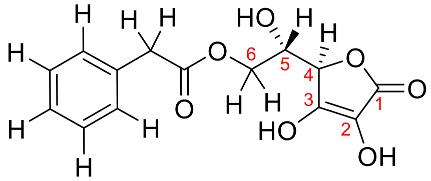

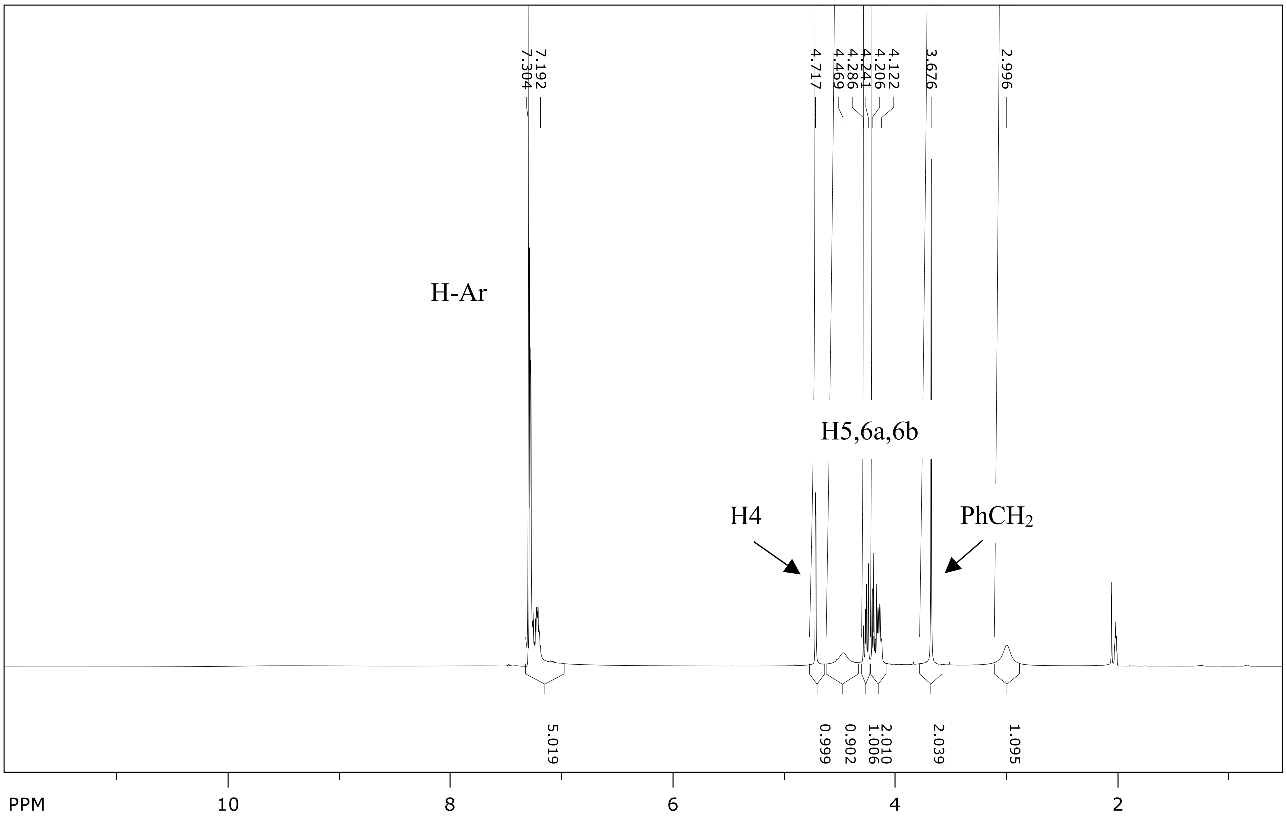

Figure S32. ^{1}H NMR (400 MHz, $(CD_3)_2CO$) of Asc-PA

δ (ppm) 7.30-7.19 (m, 5H, Ar-H), 4.72 (d, *J* = 3.6 Hz, 1H, H4), 4.47 (br, 1H, *O*3-H), 4.24-4.29 (m, 1H, H5), 4.12-4.21 (m, 2H, H6a, H6b), 3.68 (s, 2H, $PhCH_2$), 3.00 (br, 1H, *O*2-H**)**.

Asc-PA NMR characterization (continued)

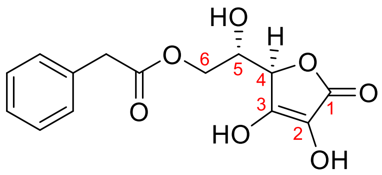

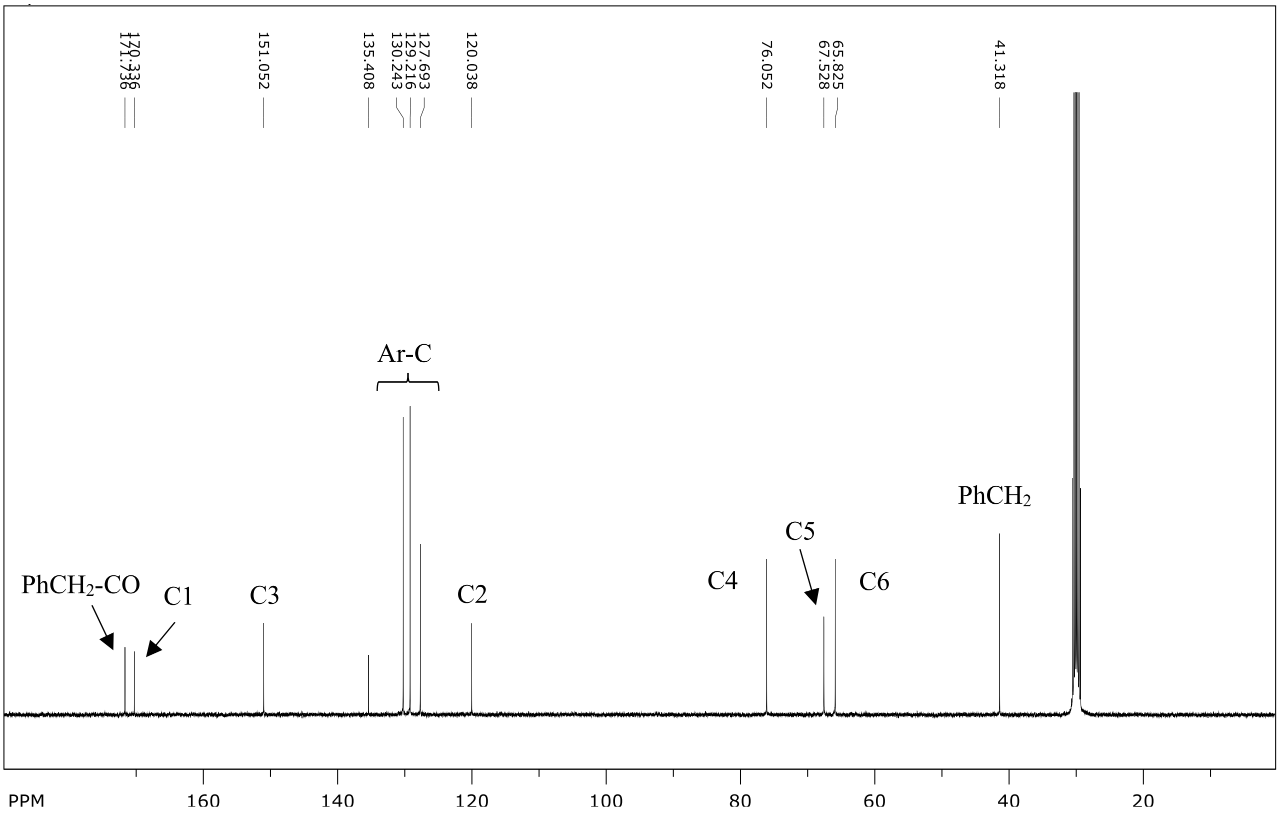

Figure S33. ^{13}C NMR (100 MHz, $(CD_3)_2CO$) of Asc-PA

δ (ppm) 171.7 (PhCH$_2$<u>C</u>O), 170.3 (C1), 151.1 (C3), 135.4 (Ar *ipso*-C), 130.2 (Ar o*rtho*-C), 129.2 (Ar *meta*-C), 127.7 (Ar *para*-C), 120.0 (C2), 76.1 (C4), 67.5 (C5), 65.8 (C6)*, 41.3 (Ar-<u>C</u>H$_2$)*.

* Signals appear as negative peaks in ^{13}C-DEPT analysis.

Thr-PA NMR characterization

The ^{1}H-NMR spectrum of Thr-PA (Figure S34) displayed three proton signals of equal intensity between 3.5 and 4.1 ppm, which can be assigned to the protons of threonate. In addition, a singlet representing two protons can be assigned to the CH_2 group of phenylacetate ($PhCH_2$). The COSY showed strong coupling between the two H6 protons at 4.03 and 3.88 ppm (Figure S35). Additional coupling appears between H5 (3.81 ppm) and the two H6 protons as well as between H5 and H4 (3.56 ppm). As expected, there were no crosspeaks for the isolated CH_2 group at 3.66 ppm. The ^{13}C-NMR spectrum (Figure S36) displayed 3 upfield signals at 66.3, 69.6 and 70.1 ppm, which can be assigned to C6, C5 and C4, respectively. The signal at 174.6 ppm was assigned to the carboxylic group at C4 based on ^{13}C and HMBC analysis, which depicts correlations with H5 (2-bond) and H6 (3-bond) (Figure S37). The phenylacetate group shows an array of 6 signals that depict the ^{13}C signals for the aromatic phenyl ring in a ratio of 1:2:2:1. Although the CH_2 group of the phenylacetate moiety is masked by that of DMSO (38.6-40.4 ppm), the signal was observed as a small signal immediately before DMSO and the DEPT spectrum depicted this group as expected as a negative signal at 40.2 ppm. The signal at 171.3 ppm can be assigned to the corresponding carbonyl group of the phenylacetate moiety in view of its correlation with CH_2 (2-bond) in the HMBC spectrum.

Thr-PA NMR characterization (continued)

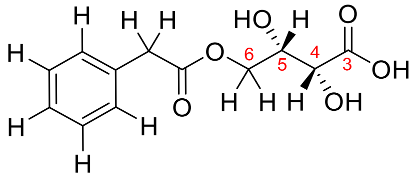

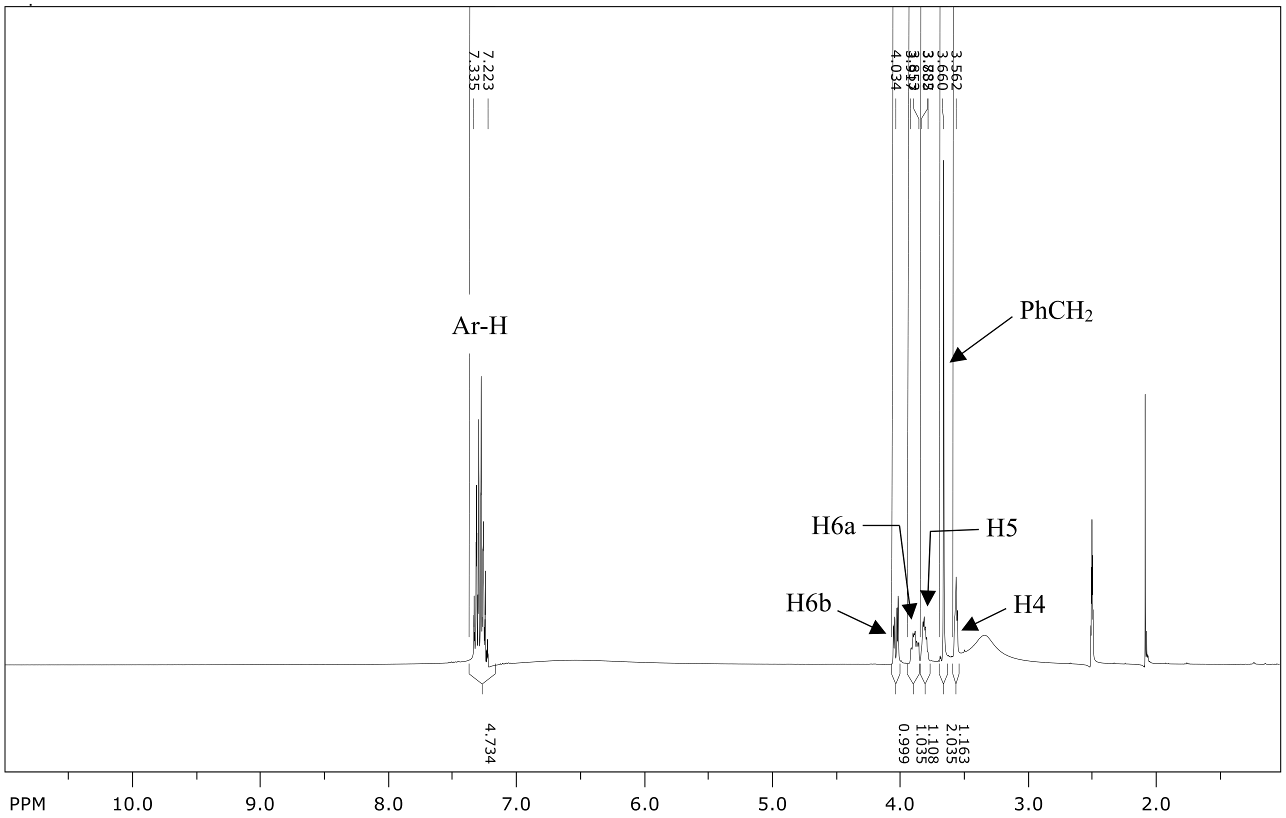

Figure S34. ^{1}H NMR (400 MHz, $(CD_3)_2SO$) of Thr-PA

δ (ppm) 7.34-7.22 (m, 5H, Ar-H), 3.81 (dd, J = 4.1, 10.8 Hz, 1H, H5) 4.03 (m, 1H, H6a), 3.88 (m, 1H, H6b), 3.66 (s, 2H, $PhCH_2$), 3.56 (dd, 1H, H4).

Thr-PA NMR characterization (continued)

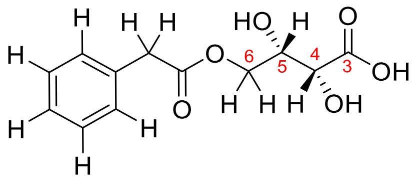

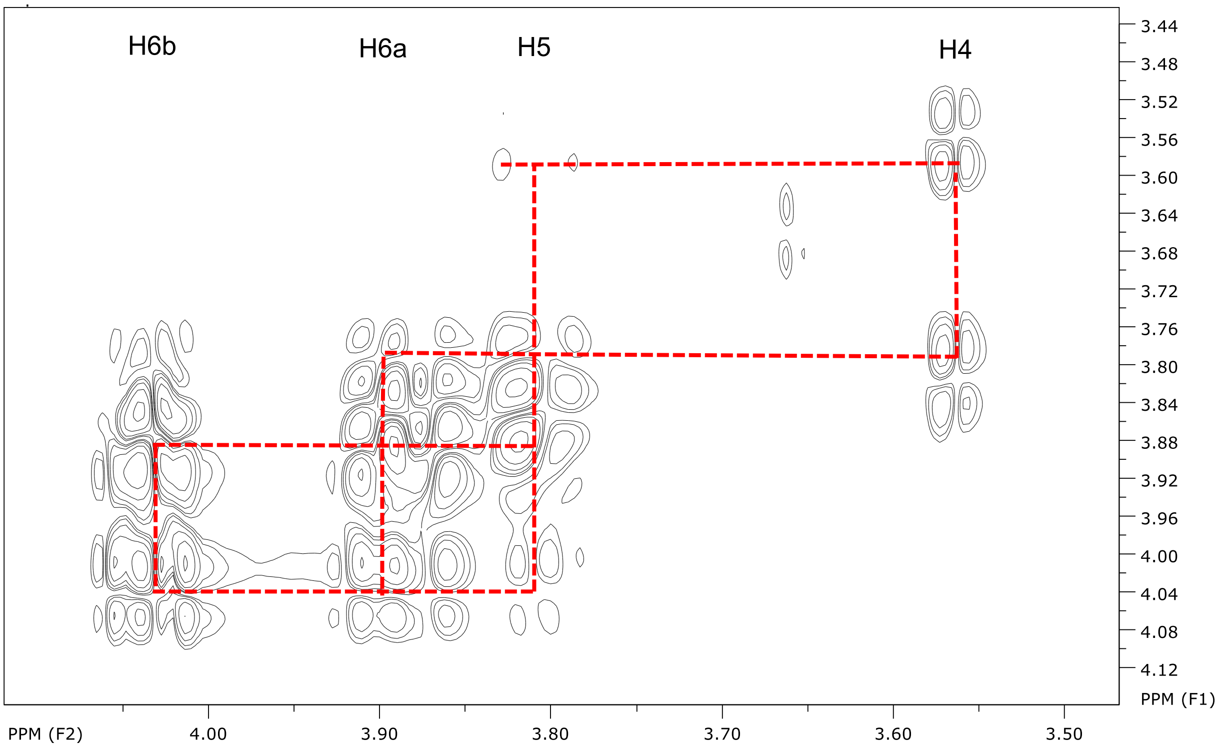

Figure S35. COSY 2D NMR (400 MHz, $(CD_3)_2SO$) of Thr-PA.

Thr-PA NMR characterization (continued)

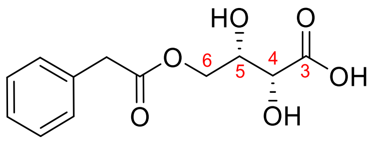

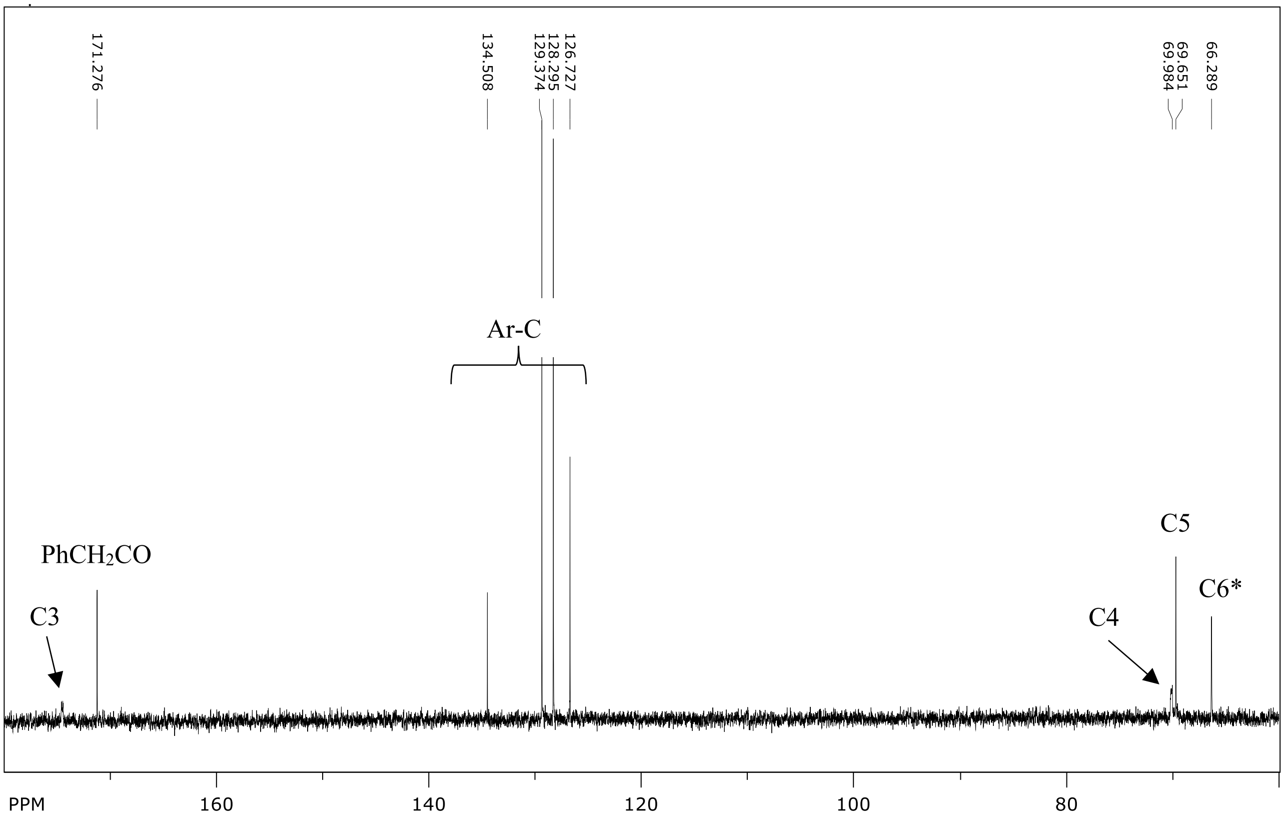

Figure S36. ^{13}C NMR (100 MHz, $(CD_3)_2SO$) of Thr-PA

δ (ppm) 174.6 (C3), 171.3 ($PhCH_2\underline{C}OOR$), 134.5 (Ar *ipso*-C), 129.4 (Ar *ortho*-C), 128.3 (Ar *meta*-C), 126.7 (Ar *para*-C), 70.1 (C4), 69.6 (C5), 66.3 (C6)*, 40.2 ($Ph\underline{C}H_2$)*.

* Signals appear as negative peaks in ^{13}C-DEPT analysis. Note that the signal for $PhCH_2$ overlapped with those for $(CD_3)_2SO$ but was clearly visible in the DEPT spectrum.

Thr-PA NMR characterization (continued)

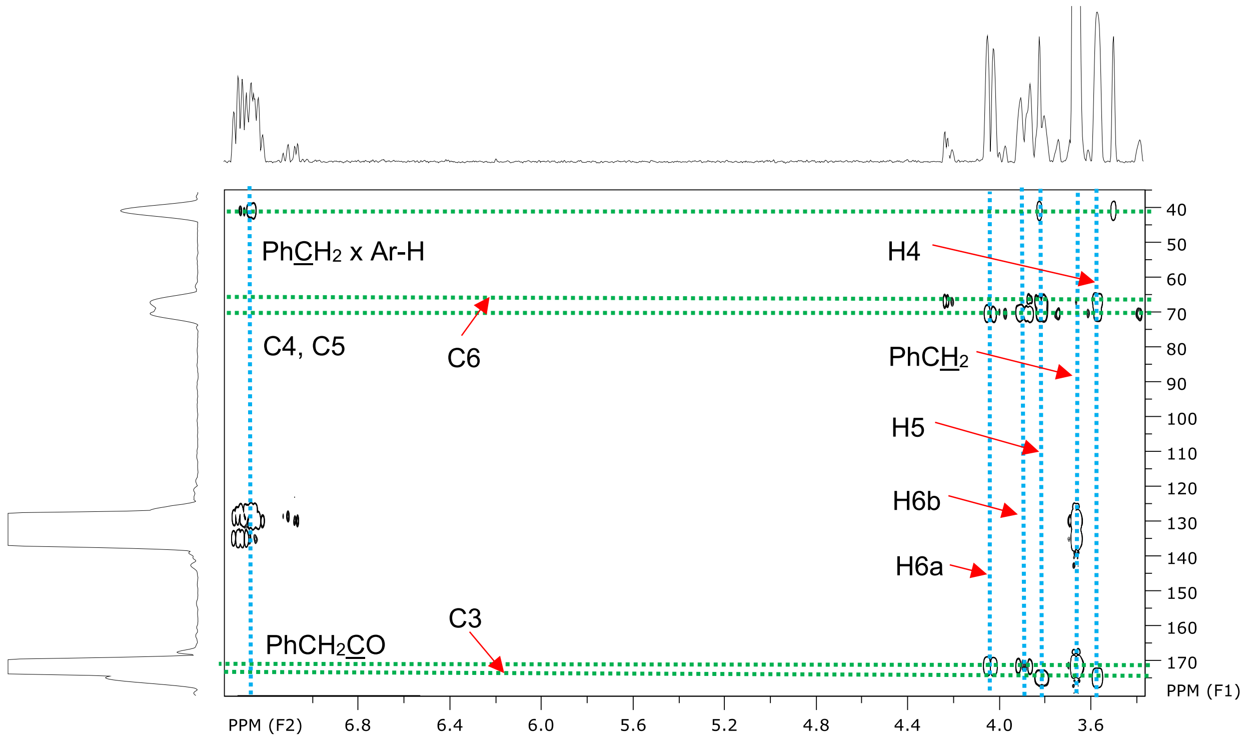

Figure S37. HMBC 2D NMR (400 MHz, $(CD_3)_2SO$) of Thr-PA.

cOxa-Thr-PA NMR characterization

This product was prepared in situ for NMR analysis, and thus, the reagent (mCPBA) and main byproduct of the reagent (mCBA) appear in the spectra. The ^{1}H signals from mCBA and mCBA, which appear between 7.50 and 8.00 ppm, however, do not interfere with the signals from the product. The ^{1}H-spectrum shows six protons that can be assigned to cOxa-Thr-PA (Figure S38, S39): 2 protons at 3.68 ppm that can be assigned to the CH_2 group of $PhCH_2$ followed with two protons at H6 (H6a and H6b: 4.64 and 4.84 ppm), H5 (5.37 ppm) and H4 (5.66 ppm). The COSY spectrum (Figure S40) displays strong geminal coupling between H6a and H6b, moderate coupling between H5 and H6a, H6b, and weaker coupling involving H4 and H5. There are two important regions of the ^{13}C-NMR spectrum (Figure S41). The region between 60-80 ppm shows three signals for C4, C5, C6 (Figure S42). The ^{13}C signals for C4 and C5 were separated by only 0.02 ppm. The region between 152-174 ppm (Figure S43) contains prominent ^{13}C signals at 152.2, 153.2, 169.2 and 171.1 ppm. The signal at 166.4 ppm is a byproduct of the mCPBA reaction. The connectivity of carbon atoms with protons was determined from HMBC analysis (Figure S44). The HMBC analyses were carried out using a slightly modified pulse sequence. Normally, the first ^{13}C 90 degree pulse, which is applied $1/(2\ ^{1}J_{CH})$ after the ^{1}H 90 degree pulse, serves as a J-filter to suppress one-bond C-H correlations. Omission of this pulse allows for one bond correlations to appear in the spectra as satellite peaks on each side of the proton such that it indicates the position of the attached carbon atom. Thereby, the carbon attached to H6 can be assigned to 64 ppm, that at H5 to 76.0 ppm, that at H4 to 76.0 ppm, and that at $PhCH_2$ to 41.0 ppm. The HMBC spectrum shows the expected coupling between H4, H5, H6 and $PhCH_2$ and C4, C5, and C6 and $PhCH_2CO$ with 2-bond and 3-bond connectivity. Both H6a and H6b were correlated to the carbonyl of $PhCH_2CO$ located at 171.1 ppm. Furthermore, HMBC analysis showed crosspeaks between H4 (5.66 ppm) and C1 (152.5 ppm-2-bond) and C3 (169 ppm-3-bond); thereby, identifying two of the three carbonyl groups of the cyclic oxalate moiety. The third carbonyl (C2) is out of range for coupling with H4 and thus was assigned to the carbonyl signal at 153.2 ppm observed in direct ^{13}C-NMR analysis (Figure S42). The experimental chemical shift of ^{13}C and ^{1}H atoms are consistent with the values obtained from predicted spectra (Figure S52).

cOxa-Thr-PA NMR characterization (continued)

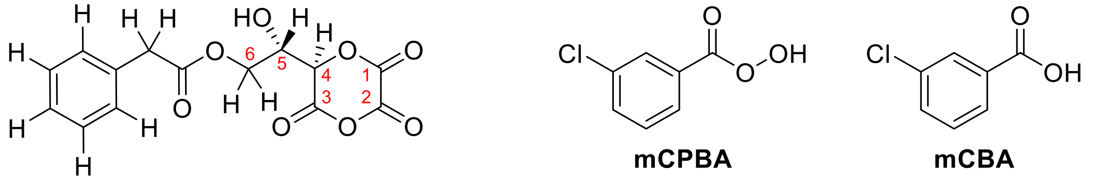

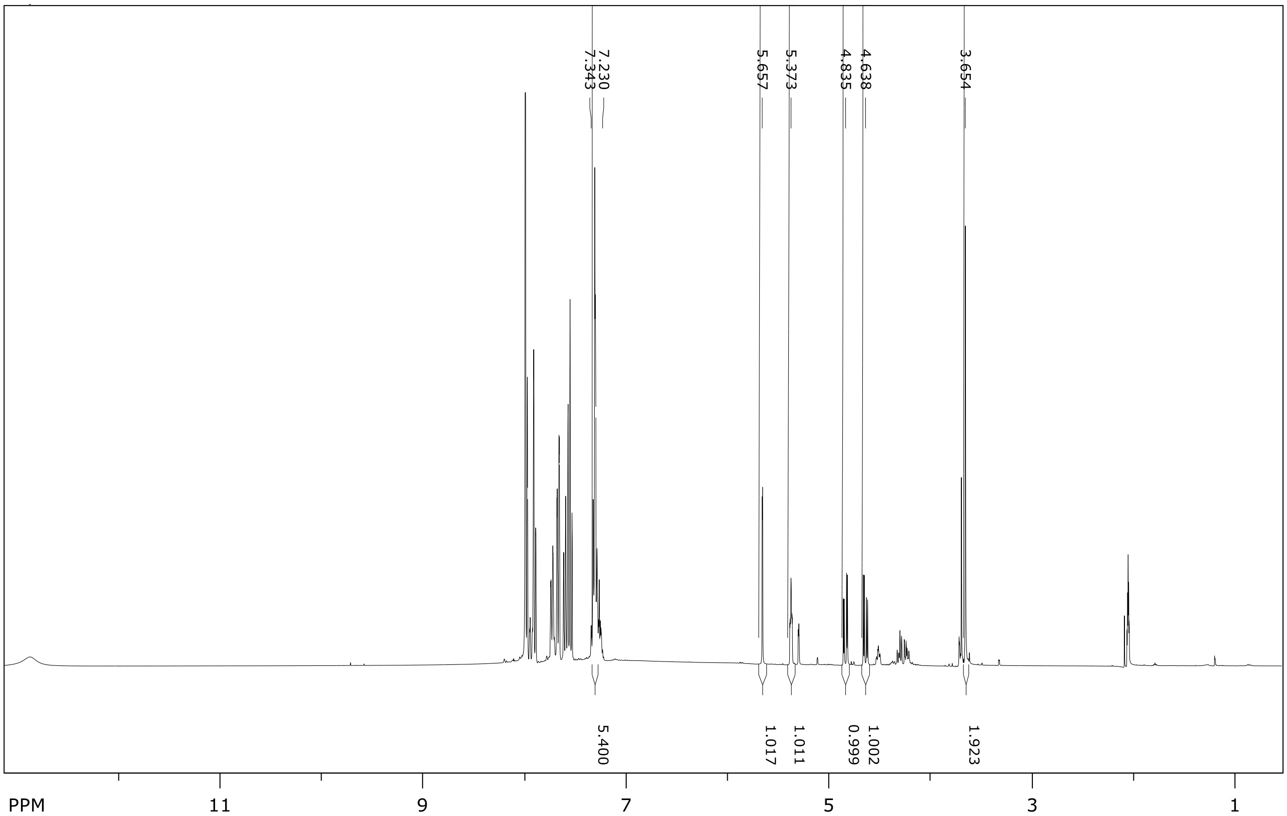

Figure S38. ^{1}H NMR (400 MHz, $(CD_3)_2CO$) of cOxa-Thr-PA

δ (ppm) 7.34-7.22 (m, 5H, Ar-H), 5.66 (d, *J* = 1.6 Hz, 1H, H4), 5.37 (ddd, *J* = 1.7, 3.0, 4.6 Hz, 1H, H5), 4.84 (dd, *J* = 12.8, 3.4 Hz, 1H, H6a), 4.64 (dd, *J* = 12.8, 4.5 Hz, 1H, H6b), 3.65 (s, 2H, $PhCH_2$).

<u>Other components</u> :

Oxa-Thr-PA : δ (ppm) 7.34-7.22 (m, 5H, Ar-H), 5.30 (d, *J* = 1.7 Hz, 1H, H4), 4.52 (dd, *J* = 2.6, 3.7, 6.6 Hz, 1H, H5), 4.31 (dd, *J* = 6.5, 11.1 Hz, 1H, H6a), 4.24 (dd, *J* = 6.7, 11.1 Hz, 1H, H6b), 3.69 (s, 2H, $PhCH_2$).

mCPBA+mCBA : δ (ppm) 12.86 (COOH), 8.05-7.50 (Ar-H).

cOxa-Thr-PA NMR characterization (continued)

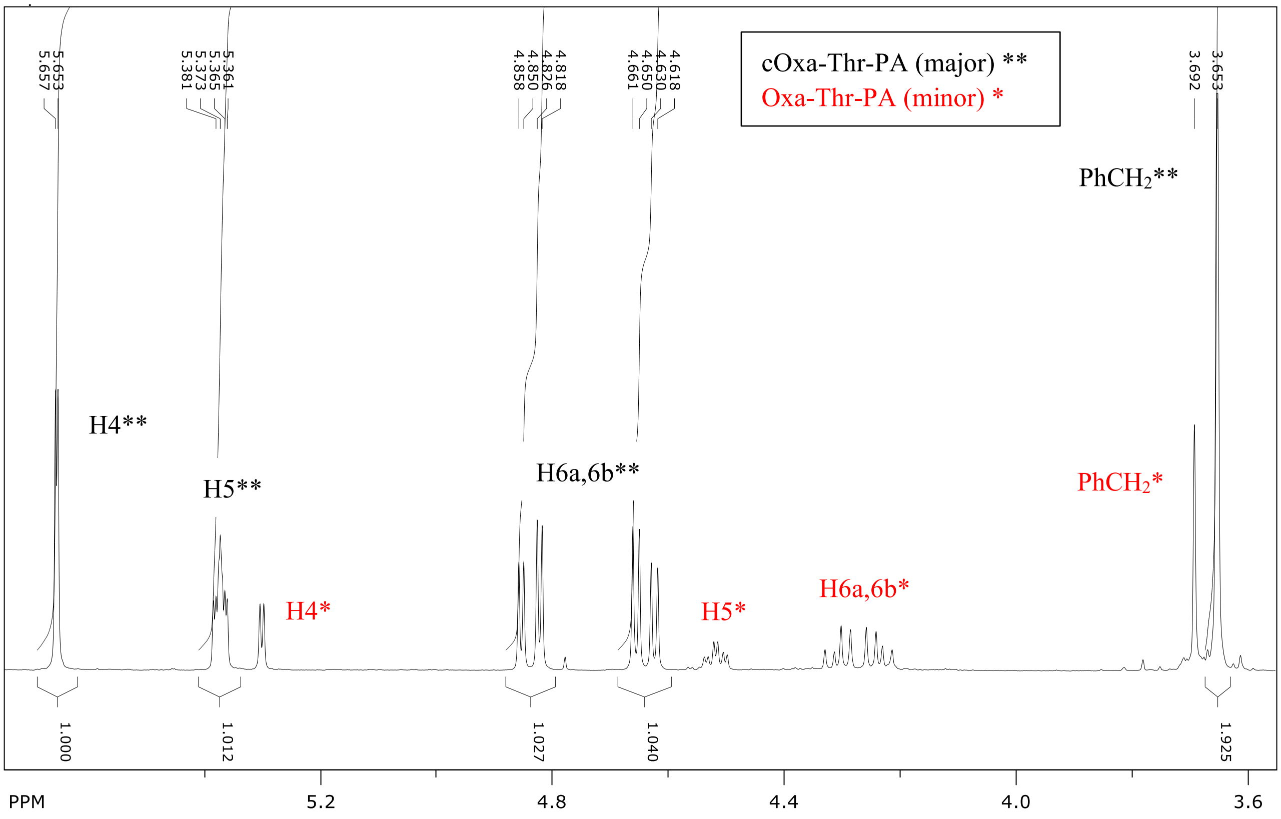

Figure S39. ^{1}H NMR (400 MHz, $(CD_3)_2CO$) of cOxa-Thr-PA (zoom).

cOxa-Thr-PA NMR characterization (continued)

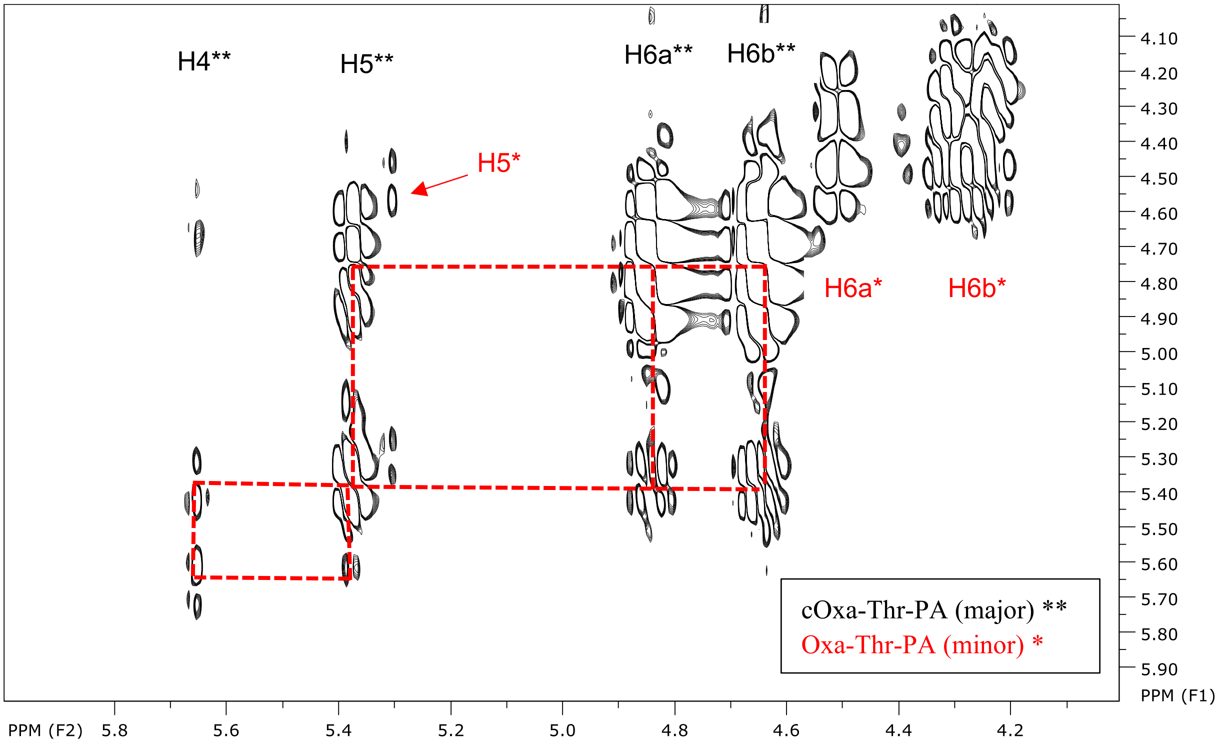

Figure S40. COSY 2D NMR (400 MHz, $(CD_3)_2CO$) of cOxa-Thr-PA**.** Signals for Oxa-Thr-PA are also present in the spectra: H4* (4.52 ppm); H6a* (4.31 ppm) and H6b* (4.24 ppm).

cOxa-Thr-PA NMR characterization (continued)

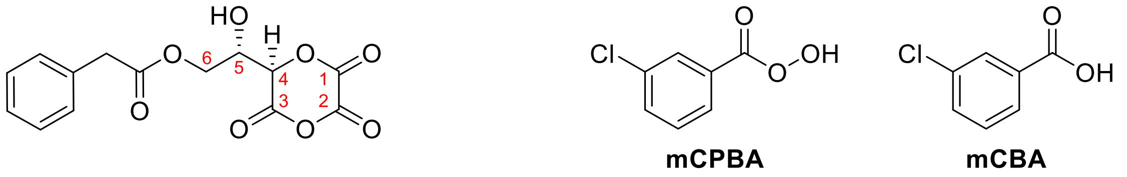

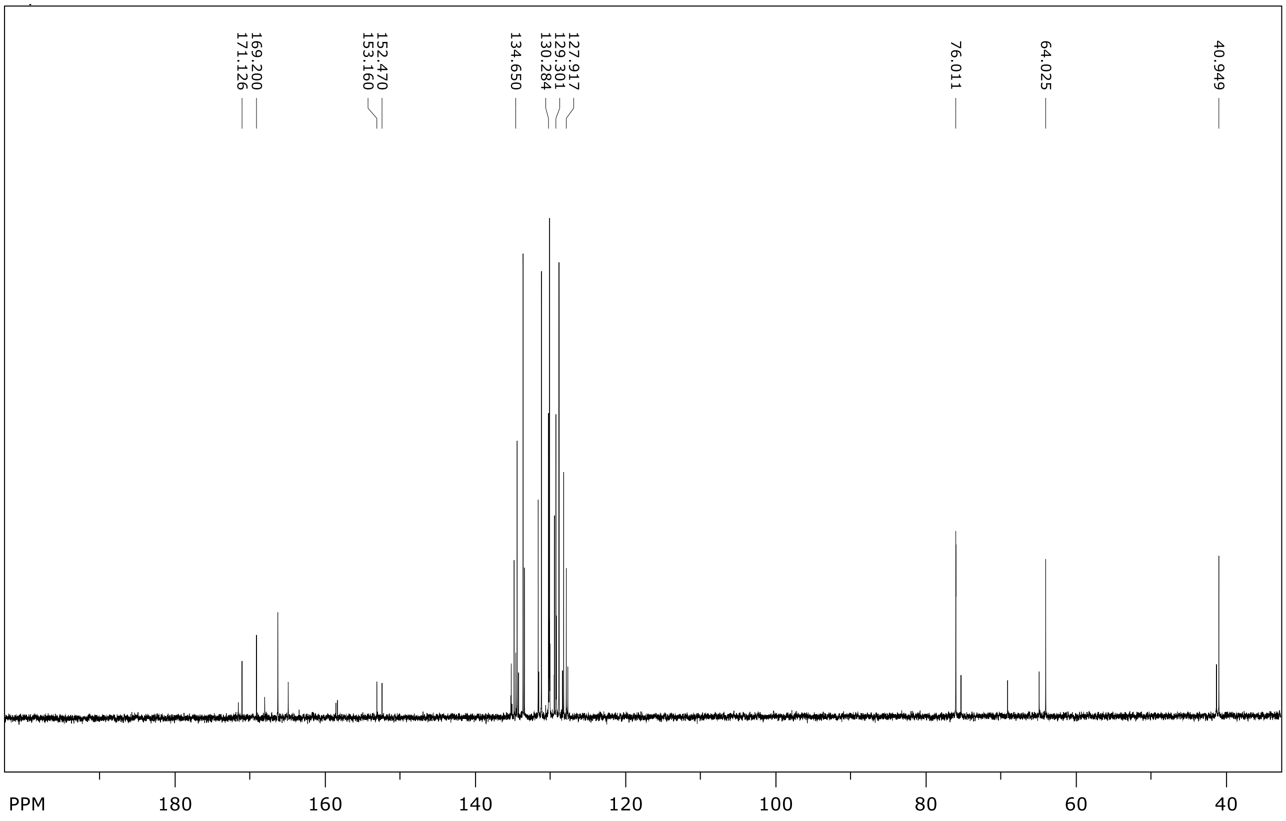

Figure S41. ^{13}C NMR (100 MHz, $(CD_3)_2CO$) of cOxa-Thr-PA

δ (ppm) 171.1 ($PhCH_2\underline{C}O$), 169.2 (C3), 153.2 (C2), 152.5 (C1), 134.7 (Ar *ipso*-C), 130.3 (Ar *ortho*-C), 129.3 (Ar *meta*-C), 127.9 (Ar *para*-C), 76.0 (C4), 76.0 (C5), 64.0 (C6)*, 41.0 ($Ph\underline{C}H_2$)*.

Other components :

Oxa-Thr-PA : δ (ppm) 171.6 ($PhCH_2\underline{C}O$), 168.1 (C3), 158.6 (C2), 158.4 (C1), 134.3 (Ar *ipso*-C), 130.2 (Ar *ortho*-C), 129.2 (Ar *meta*-C), 127.7 (Ar *para*-C), 75.3 (C4), 69.1 (C5), 64.9 (C6)*, 41.3 ($Ph\underline{C}H_2$)*.

mCPBA: δ (ppm) 165.0 ((C̲O)OOH), 135.3 (Ar C3), 134.5 (Ar C4), 131.7 (Ar C2), 130.1 (Ar C1), 129.5 (Ar C5), 128.3 (Ar C6).

mCBA: δ (ppm) 166.4 (C̲OOH), 134.9 (Ar C3), 133.7 (Ar C4), 133.5 (Ar C1), 131.2 (Ar C2), 130.2 (Ar C5), 128.9 (Ar C6).

* Signals appear as negative peaks in ^{13}C-DEPT analysis.

cOxa-Thr-PA NMR characterization (continued)

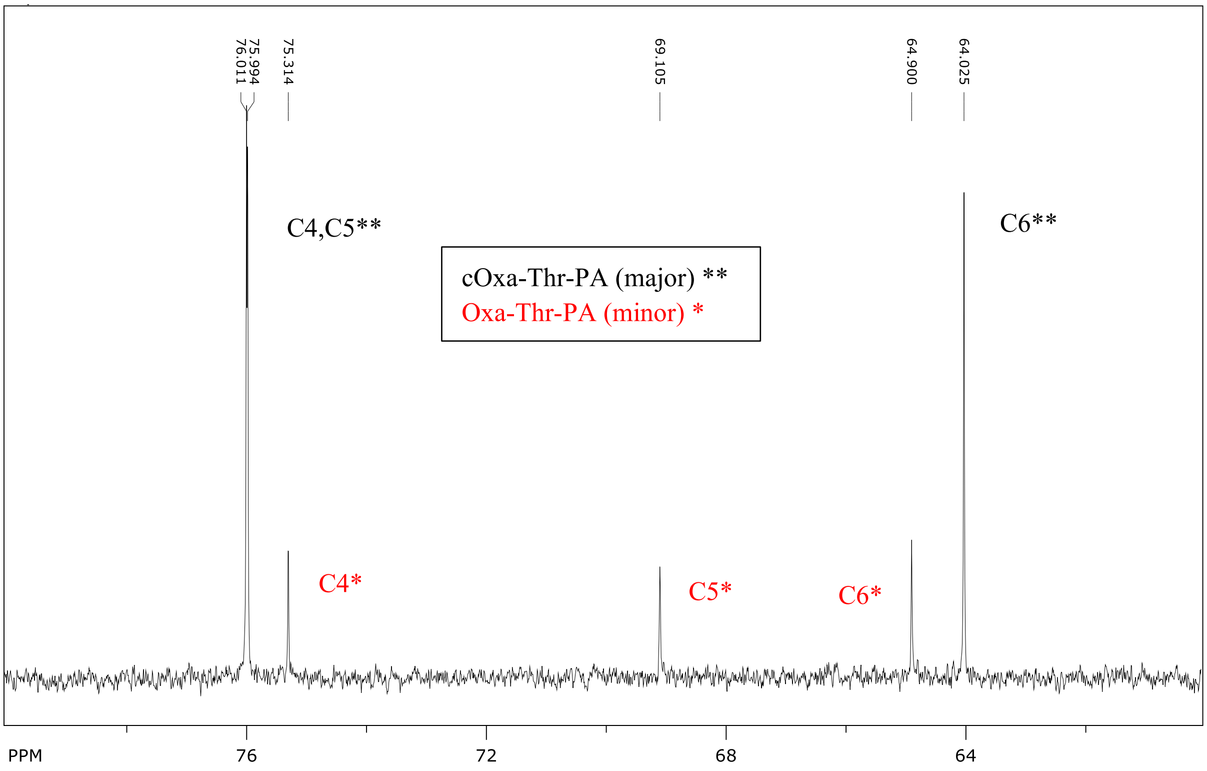

Figure S42. ^{13}C NMR (100 MHz, $(CD_3)_2CO$) of cOxa-Thr-PA (zoom 60-80 ppm).

cOxa-Thr-PA NMR characterization (continued)

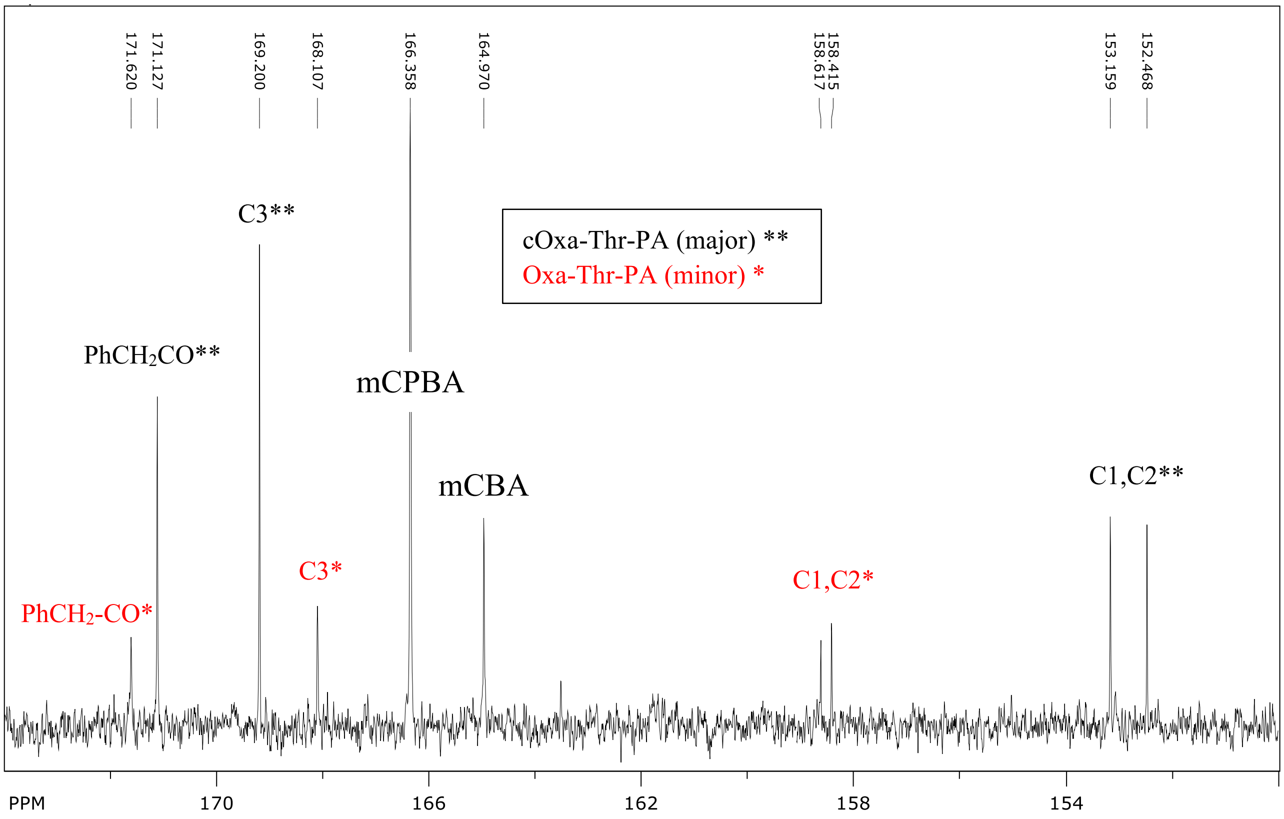

Figure S43. ^{13}C NMR (100 MHz, $(CD_3)_2CO$) of cOxa-Thr-PA (zoom 150-175 ppm).

cOxa-Thr-PA NMR characterization (continued)

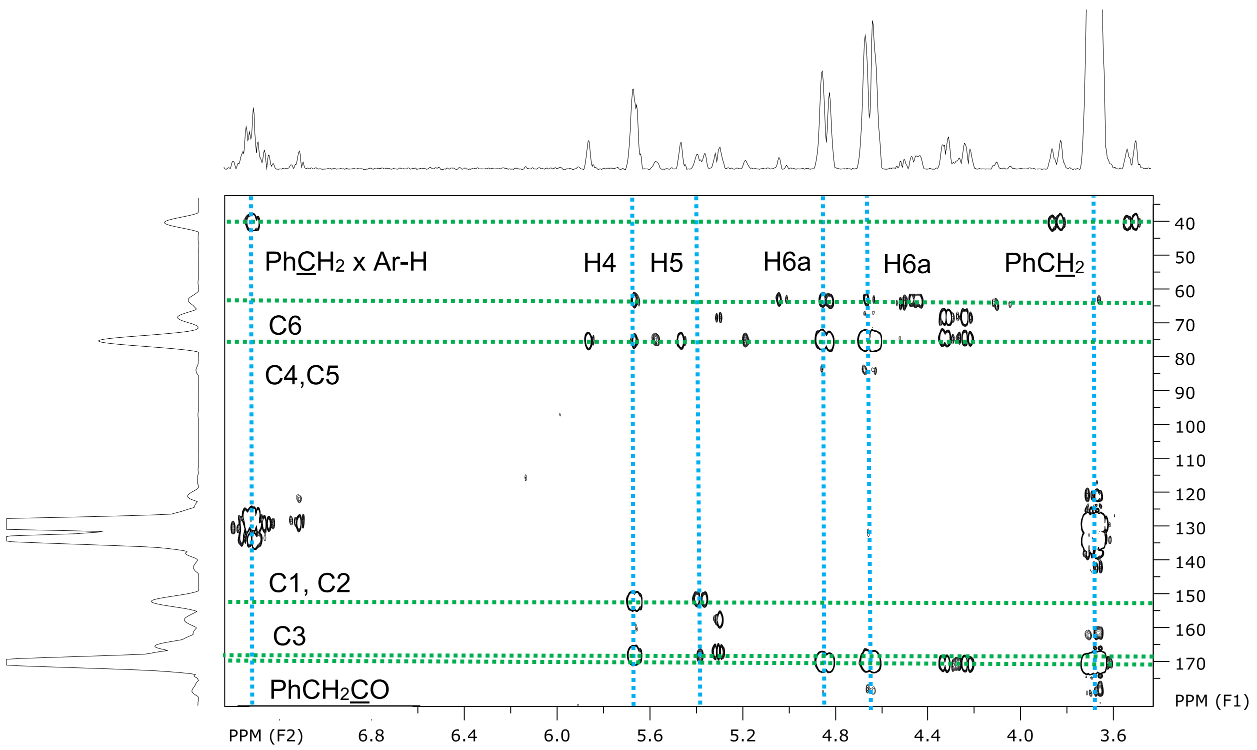

Figure S44. HMBC 2D NMR (400 MHz, $(CD_3)_2CO$) of cOxa-Thr-PA. The spectra contains crosspeaks for Oxa-Thr-PA: H4 (5.30 ppm) is correlated with C1 (158.4 ppm), C3 (168.1 ppm) and C5 (69.1 ppm); H6a and H6b are correlated with $PhCH_2\underline{C}O$ (171.6 ppm), C4 (75.3 ppm) and C5 (168.1 ppm).

Oxa-Thr-PA NMR characterization

The assignment of Oxa-Thr-PA was deduced in part from the spectra of cOxa-Thr-PA, which showed the presence of a minor component that arises from the partial hydrolysis of cOxa-Thr-PA. Within the ^{1}H-NMR spectrum of cOxa-Thr-PA (Figure S39), we observed a minor component displaying three protons in the 4-6 ppm range next to those of the major component. These protons can be assigned to H6a, H6b, H5 and H4 of the Asc side chain (-CH_2-CH(OH)-CH-); H6a and H6b appear as two quartets centered at 4.24 and 4.31 ppm, H5 appears as a multiplet centered at 4.52 ppm, and H4 as a doublet centered at 5.3 ppm. The protons from the phenylacetate moiety were nearly identical to those of cOxa-Thr-PA while the methylene protons from $PhCH_2$ were shifted slightly upfield by 0.04 ppm (Table 2). Within the ^{13}C-NMR of cOxa-Thr-PA (Figure S41), there were also several signals that represent about 1/3 of the signals for cOxa-Thr-PA that can be attributed to the minor hydrolysis product of cOxa-Thr-PA, i.e., Oxa-Thr-PA. In the region between 150 to 174 ppm (Figure S42), we observed a series of signals for the minor component at 171.6, 168.1, 158.6 and 158.4 ppm, which can be assigned to $PhCH_2$, C3, C2 and C1 of Oxa-Thr-PA. Similar to cOxa-Thr-PA, the HMBC (Figure S44, minor component) depicted correlations between H4 at 5.30 ppm with C1 at 158.4 ppm (2-bond) and C3 at 168.1 ppm (3-bond). The other carbonyl observed in the ^{13}C-NMR spectra may be assigned to C2 of the oxalate open chain structure of Oxa-Thr-PA. In the range between 60 to 80 ppm (Figure S43, minor component), we observed three signals that can be assigned to C3 at 75.3 ppm, C4 at 69.1 ppm and C5 at 64.9 ppm of Oxa-Thr-PA. The HMBC spectrum (Figure S44, minor component) supports this assignment showing correlations from H6 to C5 (2-bond), C4 (3-bond) and $PhCH_2$ (3-bond), from H5 to C4 (2-bond), C6 (2-bond), and lastly, from H4 to C5 (2-bond). The major difference in going from the cyclic (cOxa-Thr-PA) to the open chain oxalate derivative (Oxa-Thr-PA) involved a pronounced shift of H5 from 5.37 to 4.82 ppm and C5 from 76.0 to 69.1 ppm. These shifts may be attributed to the ability of the oxalate group to rotate and orientate itself above C5 and H5 in the open chain structure. Alternatively, we purified the open chain product (Oxa-Thr-PA) from the reaction mixture by HPLC and specifically subjected it to ^{1}H and ^{13}C-NMR analysis. In this case, the presence of signals from cOxa-Thr-PA as well as mCPBA and mCBA and any other byproducts of the reaction were absent. However, the purified product was unstable and gave a mixture of isomers in a ratio of 65:20:8:7 based on the ^{1}H-NMR signals at 3.81, 3.79, 3.77 and 3.74 ppm from the $PhCH_2$ group (Figure S45). The minor component observed in the spectra of the cyclic oxalate product (cOxa-Thr-PA) gave essentially the same ^{1}H- and ^{13}C-NMR spectra as the major product observed after purification of Oxa-Thr-PA (Figs. S45-S51; Table 2).

Of the four oxalate isomers observed in the spectra of Oxa-Thr-PA, it was possible to extract the ^{1}H signals from both the major (Oxa-Thr-PA-1) and minor (Oxa-Thr-PA-2) isomers representing respectively 65% and 20% of the total mixture (Figure S46). Three minor ^{1}H signals representing Oxa-Thr-PA-2 were observed at significantly different chemical shifts (5.59 (1H), 4.56 (1H) and 4.44 (1H) ppm; Figure S46, minor isomer)). Since the proton at 5.59 ppm is a multiplet, one can assign this signal to H5. The signal at 4.56 ppm is a doublet while that at 4.44 ppm is a doublet of doublets, and thus, they can be assigned to H5 and H6a/H6b, respectively. Investigation of the COSY (Figure S47, minor isomer) confirms this assignment showing that H5 (4.56 ppm) is coupled to both H4 (5.59 ppm) and H6a/H6b (4.44 ppm). Thus, these results indicate a remarkable downfield shift of H5 from 4.49 ppm for Oxa-Thr-PA-1 to 5.59 ppm for Oxa-Thr-PA-2 accompanied with an upfield shift of H4 from 5.23 ppm for Oxa-Thr-PA-1 to 4.56 ppm for Oxa-Thr-PA-2. In the ^{13}C spectra (Figs S48-S50, minor isomer), the ^{13}C signals for both the major (65%) and minor (20%) isomers were also visible in the range between 150 and 180 ppm, which represents the carbonyl groups, and in the range between 60 to 80 ppm, which represents the (-CH_2-CH(OH)-CH-) chain of Asc. By comparison with the major isomer, we assigned the second largest set of signals in each region to Oxa-Thr-PA-2. Thus, C1 and C2 of the oxalate group with nearly the same chemical shift were assigned to 161.3 and 161.7 ppm, respectively, whereas C3 with the terminal carboxylic acid group was assigned to 174.0 ppm. The shift of the carboxylic group at C3 is comparable to the shift observed for the terminal carboxylic group within Thr-PA, i.e., the derivative in which the oxalate group is absent. Lastly, the carbonyl group of $PhCH_2CO$ was assigned to 174.0 ppm in agreement with Oxa-Thr-PA-1 and other derivatives. Similar analysis of the region from 60 to 80 ppm that represent the (-CH_2-CH(OH)-CH-) chain of Asc leads to the assignment of C6 at 62.9 ppm, C5 at 69.2 and C4 at 73.4 ppm (Figure S50, minor isomer). The signal at 62.9 ppm was negative from DEPT analysis confirming that it is the methylene group at C6. Interestingly, C5 of Oxa-Thr-PA-2 moved downfield while the other two ^{13}C signals moved upfield, suggesting that the highly deshielding oxalate group has migrated from C4 to C5 position in the minor isomer. Although most HMBC correlations of the minor isomer (Figure S51, minor isomer) were weak or overlapped with those of the major isomer, H4 at 4.56 ppm of Oxa-Thr-PA-2 was clearly correlated with the C3 carbonyl group at 7.44 ppm (2 bonds). In contrast, H4 at 5.23 ppm of Oxa-Thr-PA-1 showed correlations with two carbonyl groups, C1 at 161.7 ppm (3-bond) and C3 at 170.7 ppm (2-bond). This suggests that the oxalate group of Oxa-Thr-PA-1 is no longer connected to H4 as observed in Oxa-Thr-PA-2. The lack of a strong correlation between H5 of the minor isomer and the oxalate group did not confirm the position of the oxalate group by HMBC analysis. In conclusion, NMR analysis strongly suggest that the oxalate group has migrated from C4 in the major isomer to C5 in the minor isomer.

Oxa-Thr-PA NMR characterization (continued)

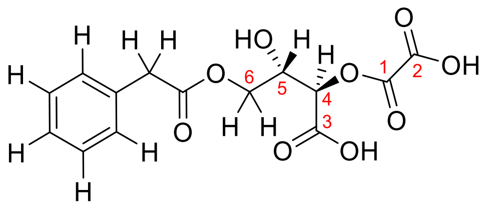

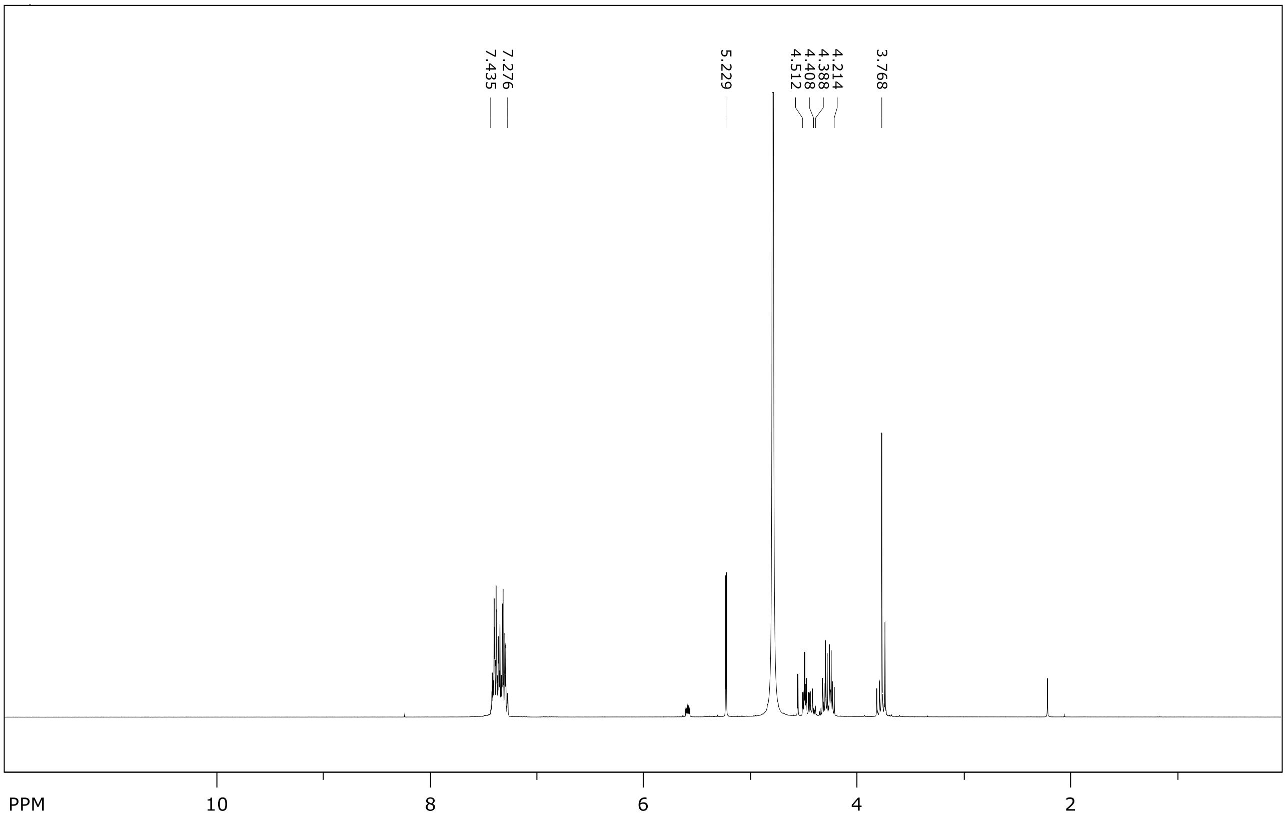

Figure S45. ^{1}H NMR (400 MHz, D_2O) of Oxa-Thr-PA

δ (ppm) 7.44-7.28 (m, 5H, Ar-H), 5.23 (d, *J* = 2.6 Hz, 1H, H4), 4.51-4.21 (m, 3H, H5, H6a, H6b), 3.77 (s, 2H, $PhCH_2$)*.

* This major peak appears with three concomitant smaller peaks (ratio 65:20:8:7), indicating that there are four isomers present, as observed by LC-MS/MS.

Oxa-Thr-PA NMR characterization (continued)

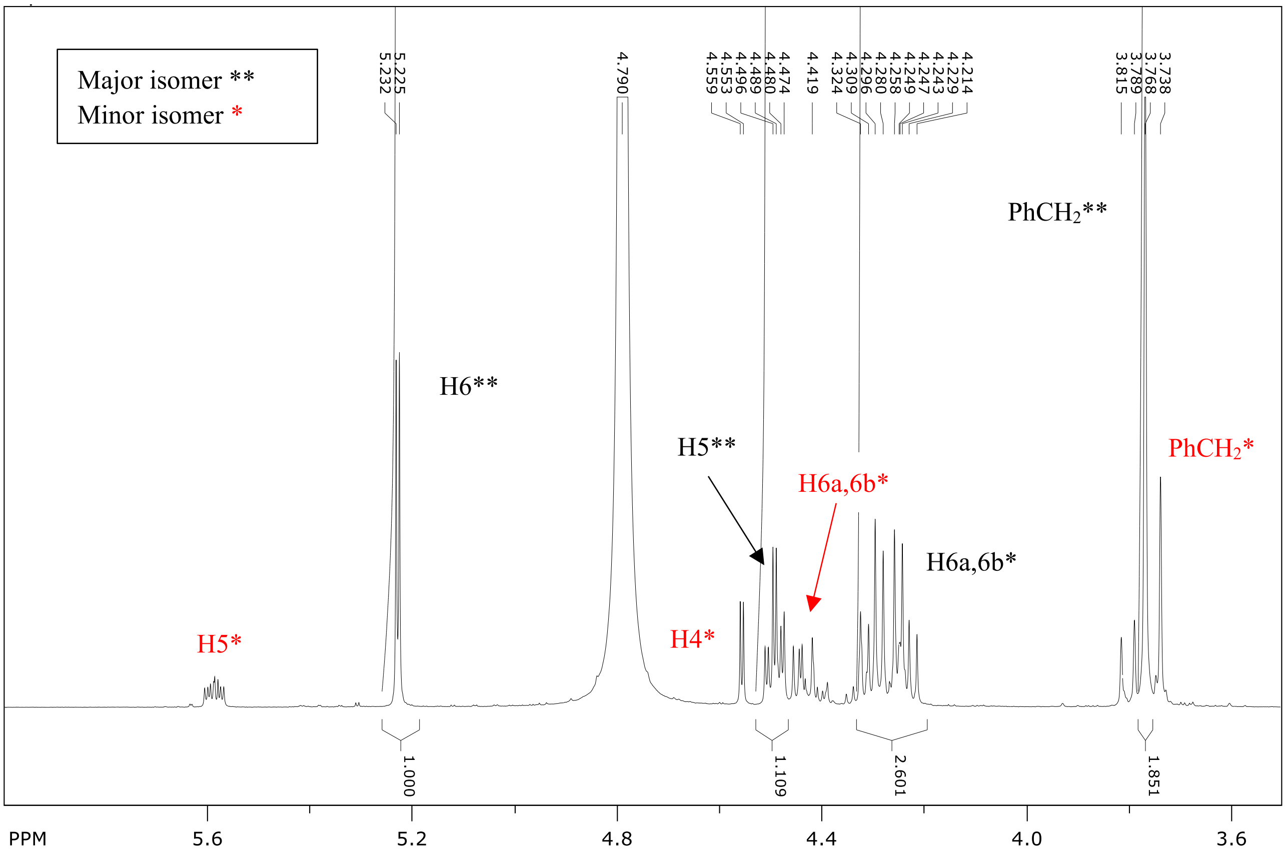

Figure S46. ^{1}H NMR (400 MHz, D_2O) of Oxa-Thr-PA (zoom).

Oxa-Thr-PA NMR characterization (continued)

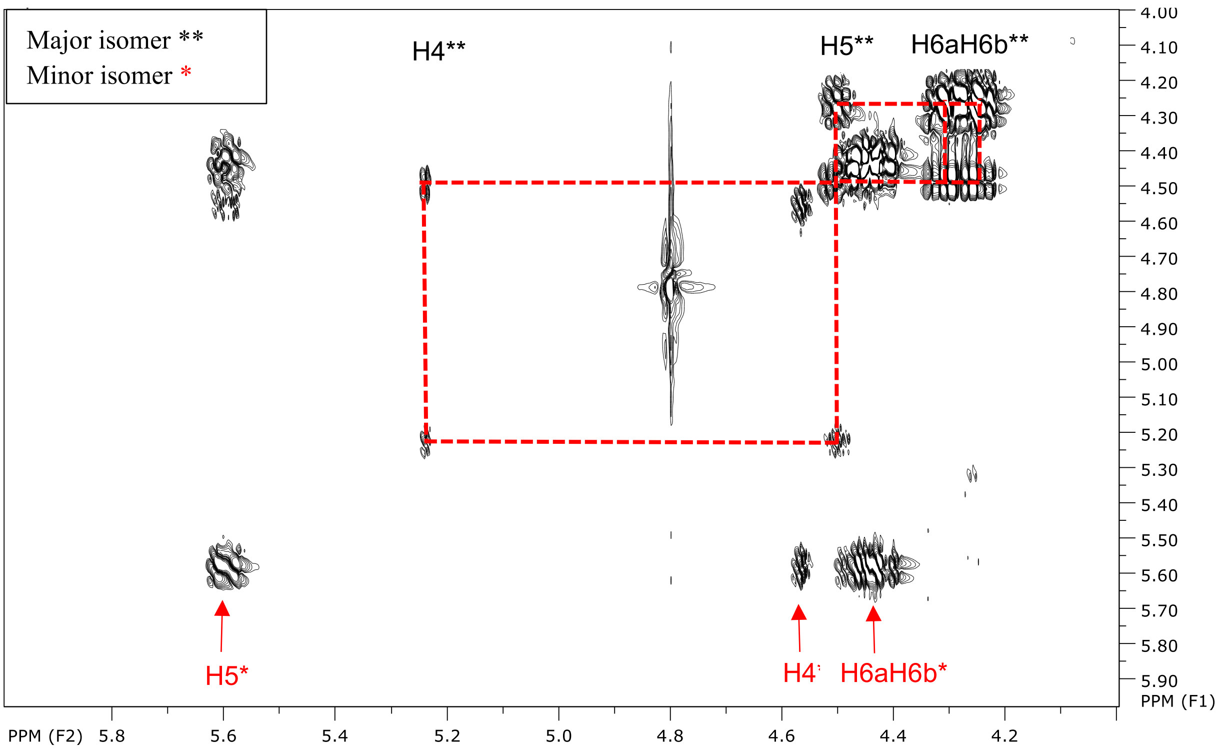

Figure S47. COSY 2D NMR (400 MHz, D_2O) of Oxa-Thr-PA. The minor isomer is more pronounced than the major isomer in this spectrum because the minor isomer is more stable and slowly converts during the time of analysis.

Oxa-Thr-PA NMR characterization (continued)

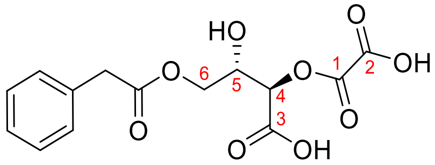

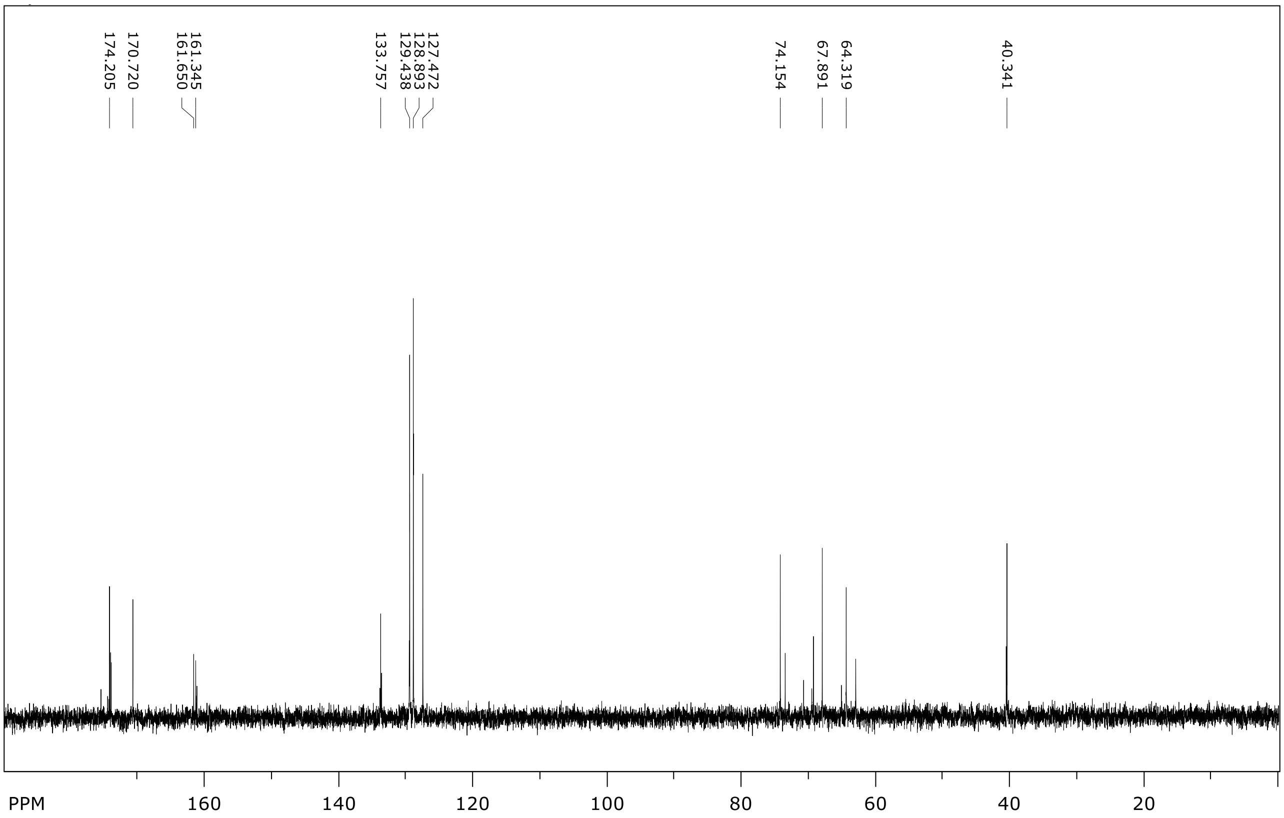

Figure S48. ^{13}C NMR (100 MHz, D_2O) of Oxa-Thr-PA

δ (ppm) 174.2 (PhCH$_2$<u>C</u>O), 170.7 (C3), 161.7 (C2), 161.3 (C1), 133.8 (Ar *ipso*-C), 129.4 (Ar *ortho*-C), 128.9 (Ar *meta*-C), 127.5 (Ar *para*-C), 74.2 (C4), 67.9 (C5), 64.3 (C6)*, 40.3 (Ph<u>C</u>H$_2$)*.

* Signals appear as negative peaks in ^{13}C-DEPT analysis.

Oxa-Thr-PA NMR characterization (continued)

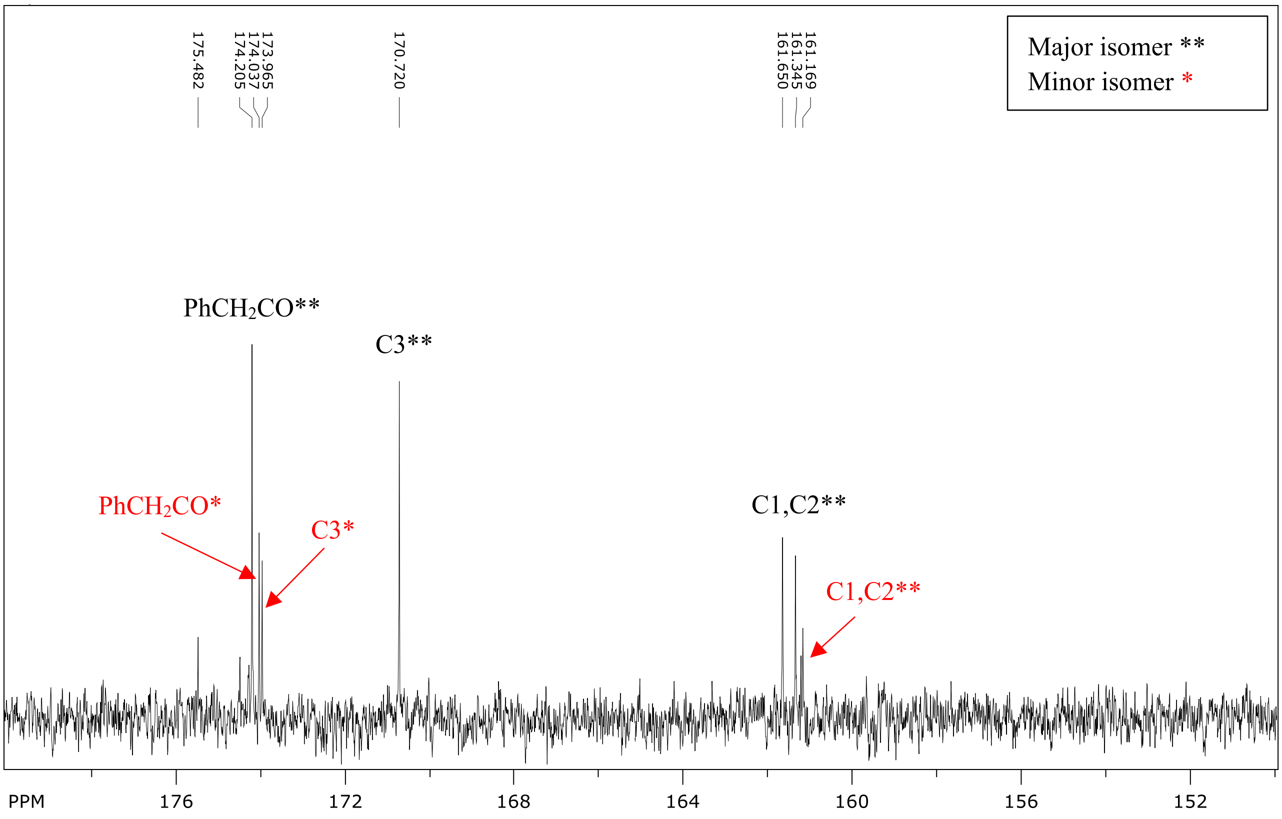

Figure S49. ^{13}C NMR (100 MHz, D_2O) of Oxa-Thr-PA (zoom 150-180 ppm).

Oxa-Thr-PA NMR characterization (continued)

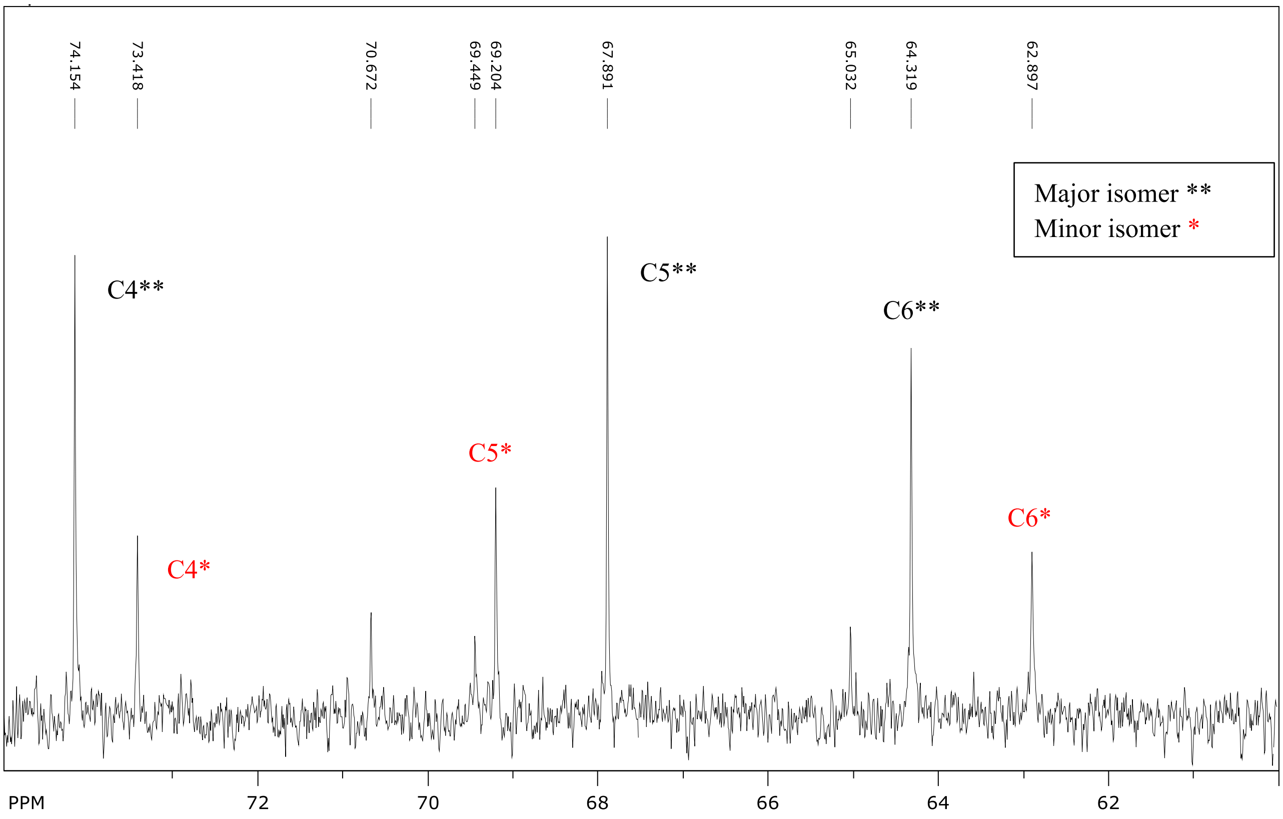

Figure S50. ^{13}C NMR (100 MHz, D_2O) of Oxa-Thr-PA (zoom 60-78 ppm).

Oxa-Thr-PA NMR characterization (continued)

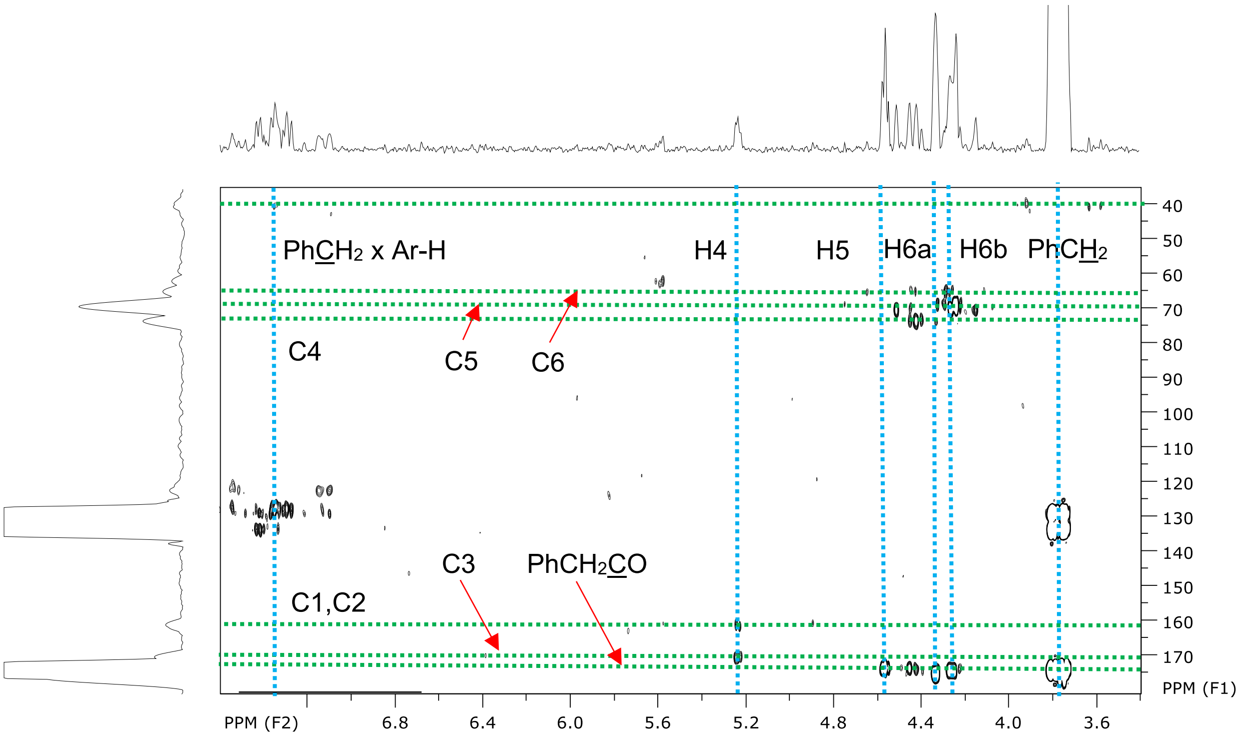

Figure S51. HMBC 2D NMR (400 MHz, D_2O) of Oxa-Thr-PA. Signals for the minor isomer were absent or overlapped with those from the major isomer. Upon amplifying the signals, weak crosspeaks were observed for the minor isomer: H5 (5.59 ppm) appeared to be correlated with C6 (62.9 ppm) and C1 (161.2 ppm).

NMR simulations of phenylacetate derivatives

	^{1}H	^{13}C
**Asc-PA**	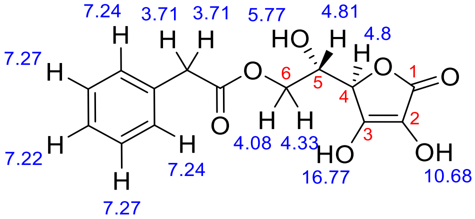	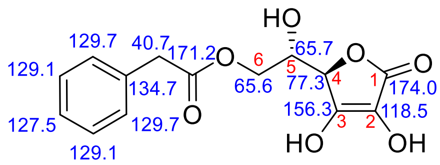
**cOxa-Thr-PA**	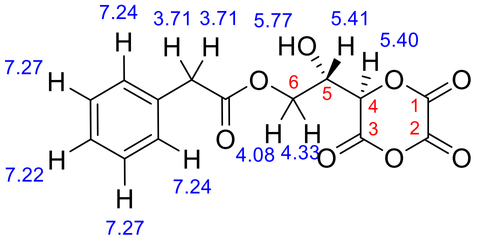	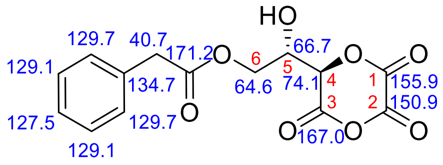
**Oxa-Thr-PA (major)**	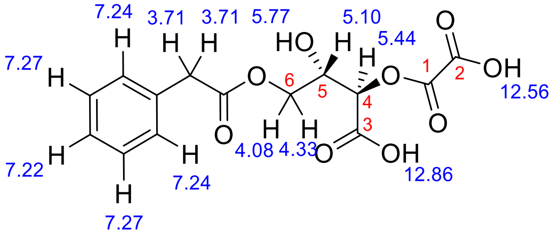	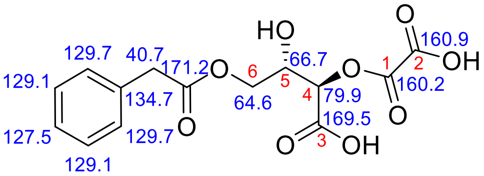
**Oxa-Thr-PA (minor)**	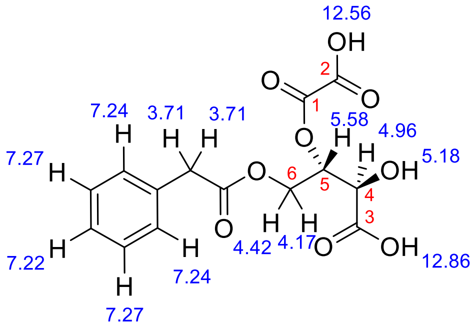	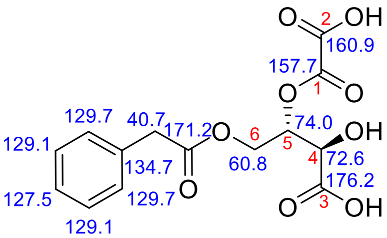
**Thr-PA**	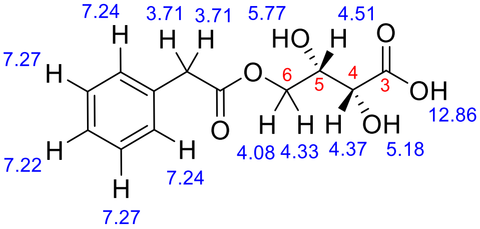	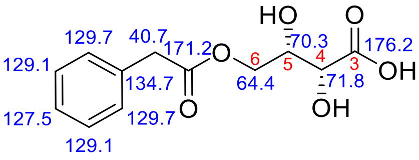

Figure S52. Simulations (in $(CD_3)_2SO$) of ^{1}H and ^{13}C NMR of phenylacetate derivatives

EDC-mediated coupling of Oxa-Thr-PA with 2-phenylethanamine

Methodology: An aqueous fraction of freshly purified Oxa-Thr-PA was treated with 10 mM solutions of 1-ethyl-3-(3′-dimethylaminopropyl)-carbodiimide hydrochloride (EDC) and N-hydroxysuccinimide (NHS) in the presence of 25 mM 2-(N-morpholino)ethanesulfonic acid (MES) for 2h at room temperature. The products were analyzed by LC-MS/MS.

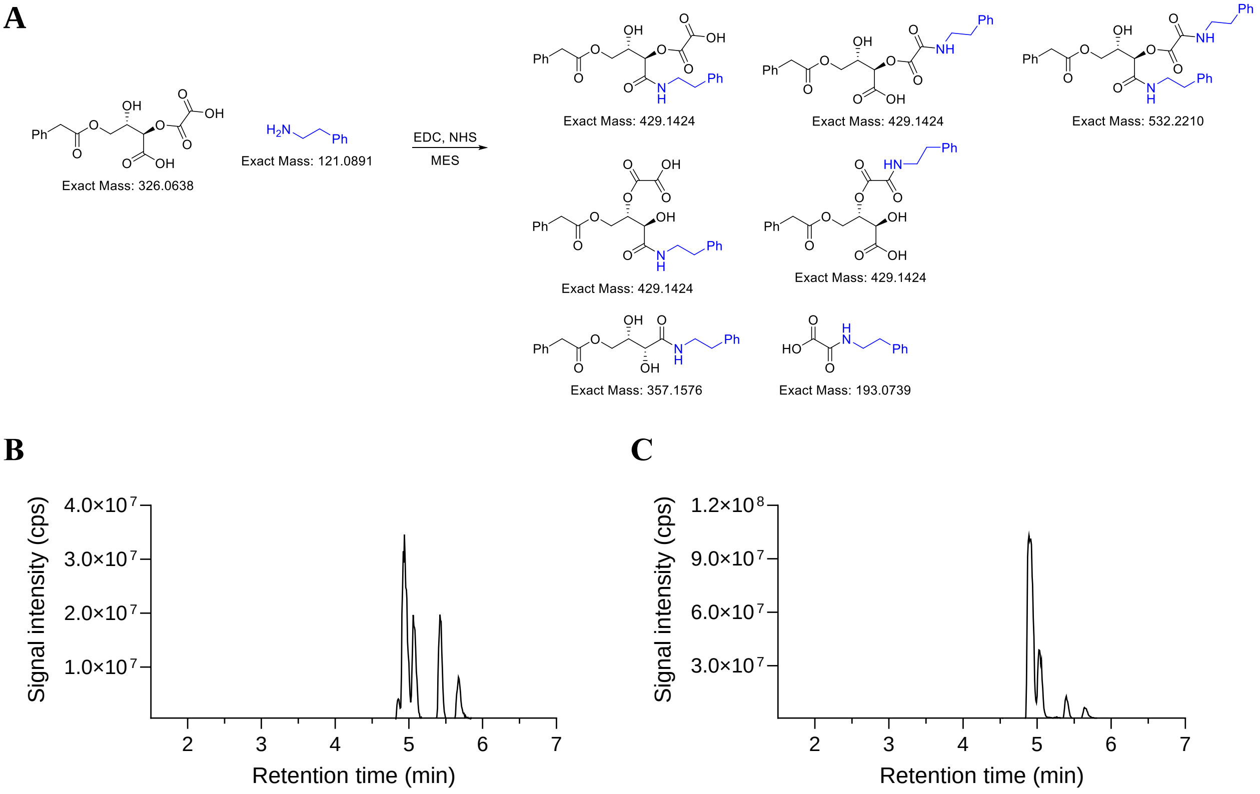

Figure S53. Coupling reaction between Oxa-Thr-PA and 2-phenylethanamine. (A) The products obtained from the reaction are four mono-amide isomers of Oxa-Thr-PA, the bis-amide derivative of Oxa-Thr-PA, the amide derivative of Thr-PA and the mono-amide derivative of oxalate. (B) LC-MS/MS of the reaction mixture (*m/z* = 428 amu, negative mode). (C) LC-MS/MS of the reaction mixture (*m/z* = 430 amu, positive mode).

EDC-mediated coupling of Oxa-Thr-PA with 2-phenylethanamine (continued)

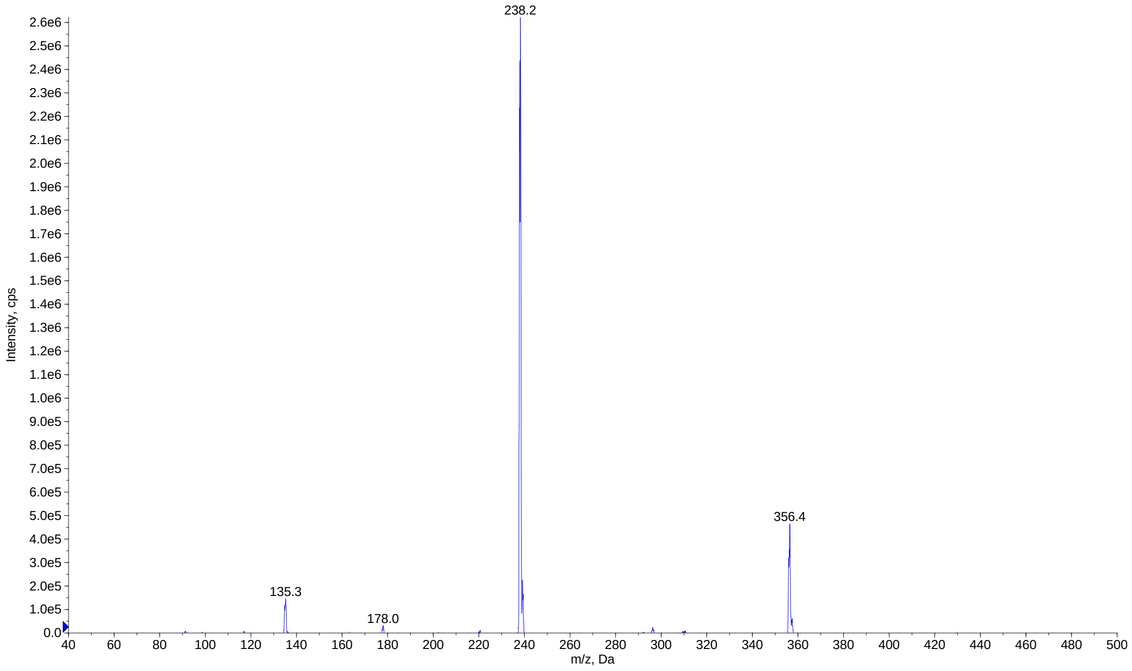

Figure S54. (-)428 MS/MS spectrum of Oxa-Thr-PA (mono-Ph$(CH_2)_2$-amide derivative #1)

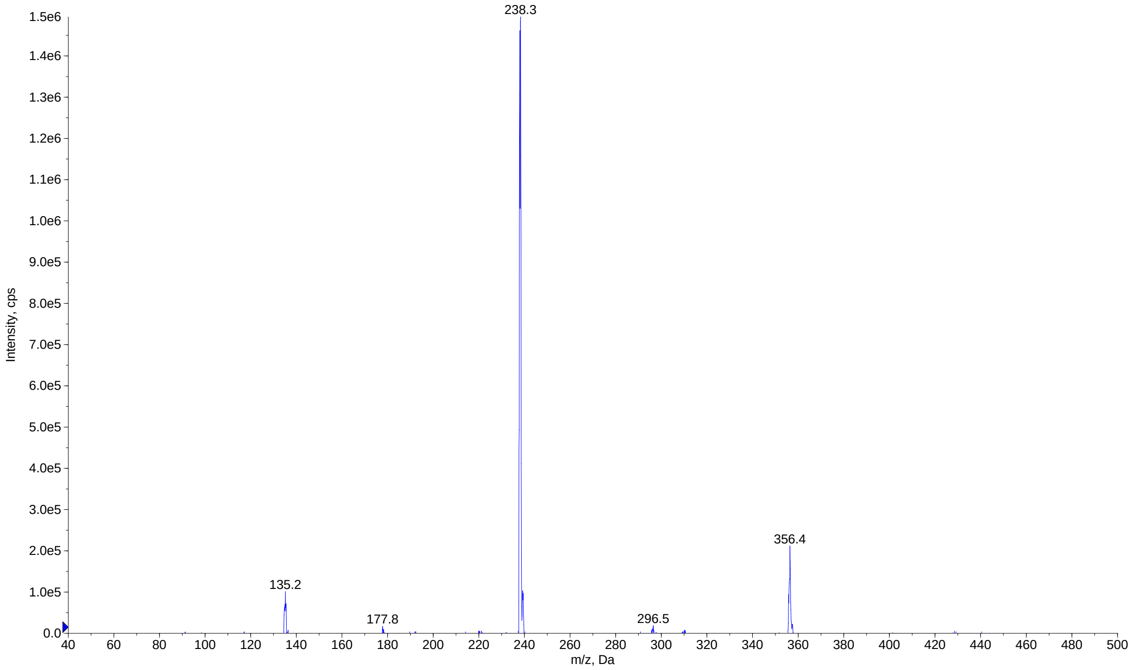

Figure S55. (-)428 MS/MS spectrum of Oxa-Thr-PA (mono-Ph$(CH_2)_2$-amide derivative #2)

EDC-mediated coupling of Oxa-Thr-PA with 2-phenylethanamine (continued)

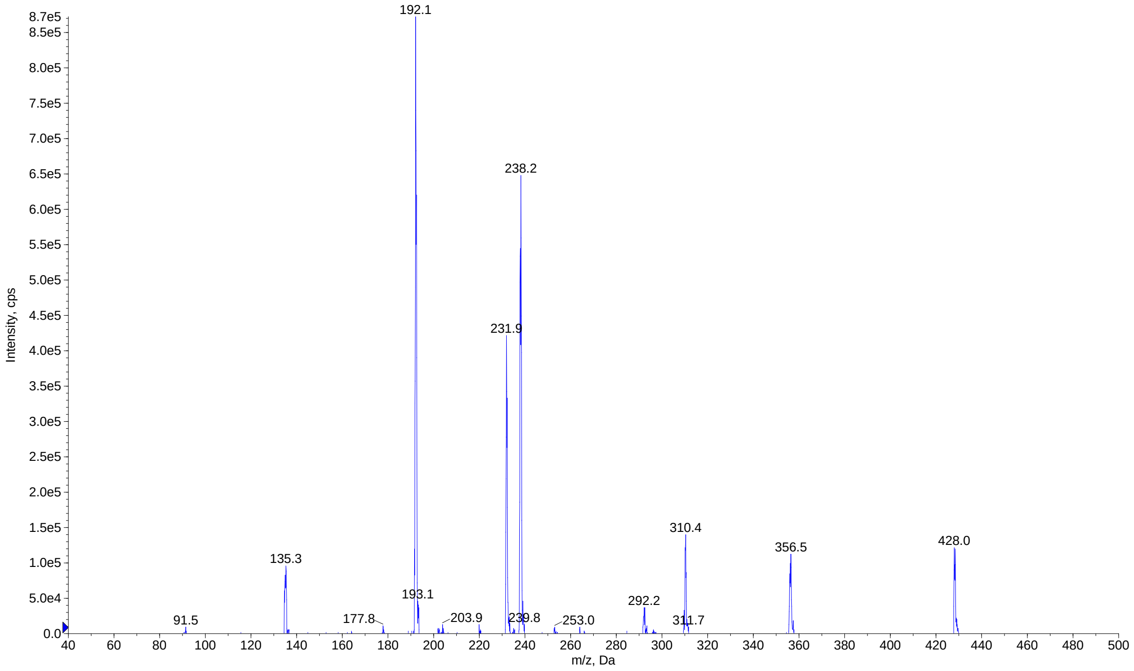

Figure S56. (-)428 MS/MS spectrum of Oxa-Thr-PA (mono-Ph$(CH_2)_2$-amide derivative #3)

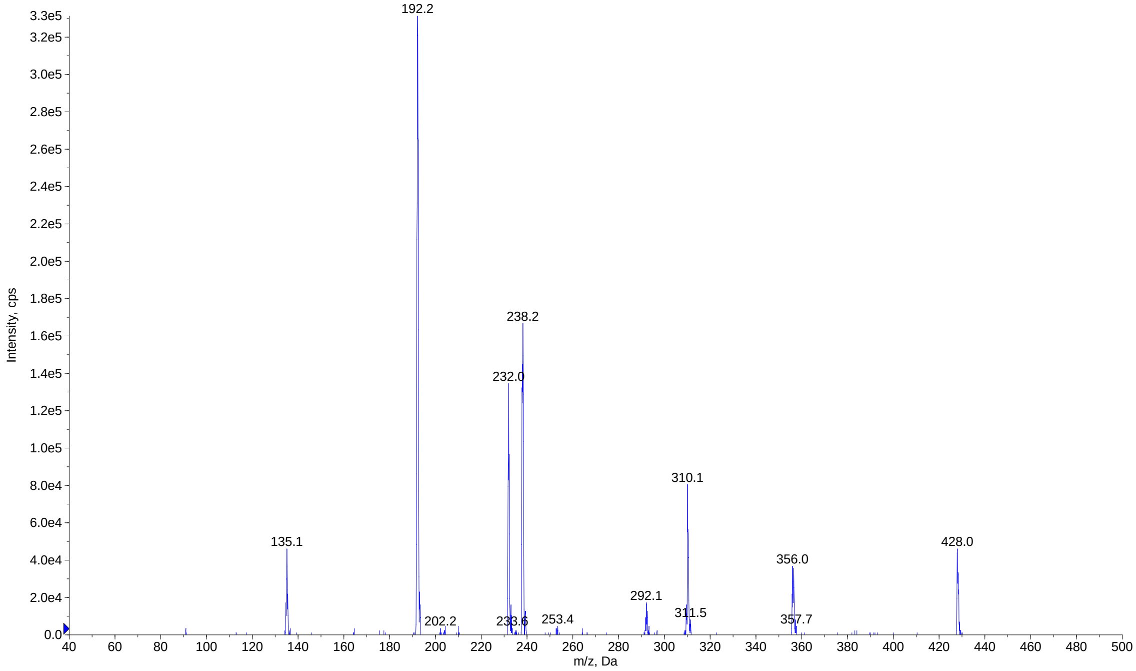

Figure S57. (-)428 MS/MS spectrum of Oxa-Thr-PA (mono-Ph$(CH_2)_2$-amide derivative #4)

EDC-mediated coupling of Oxa-Thr-PA with 2-phenylethanamine (continued)

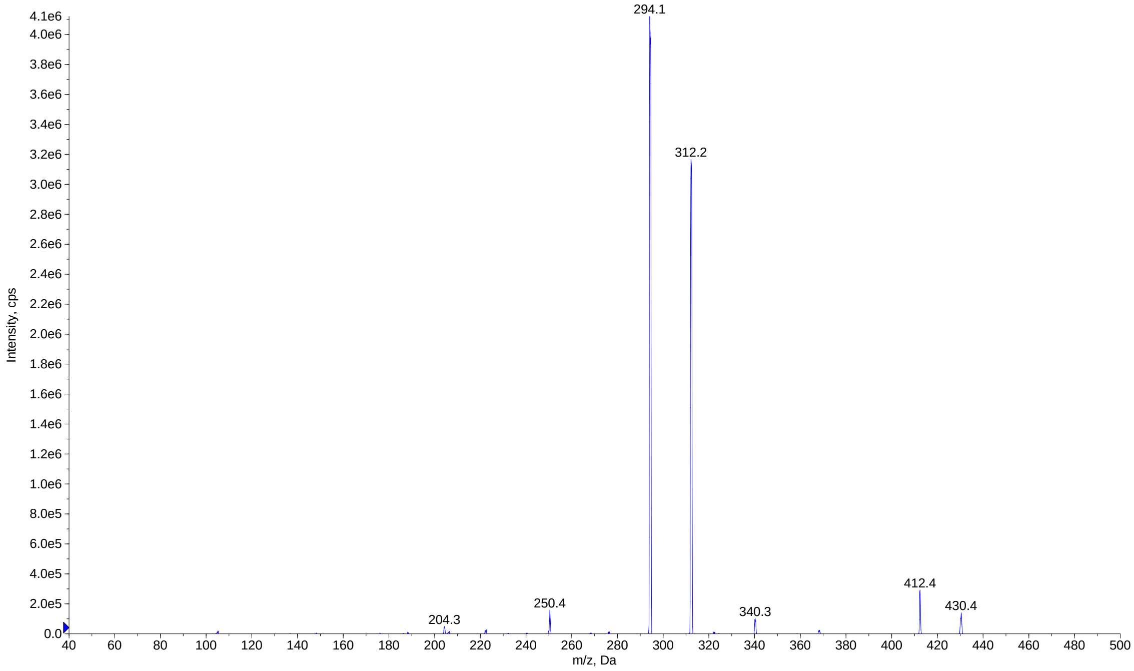

Figure S58. (+)430 MS/MS spectrum of Oxa-Thr-PA (mono-Ph$(CH_2)_2$-amide derivative #1)

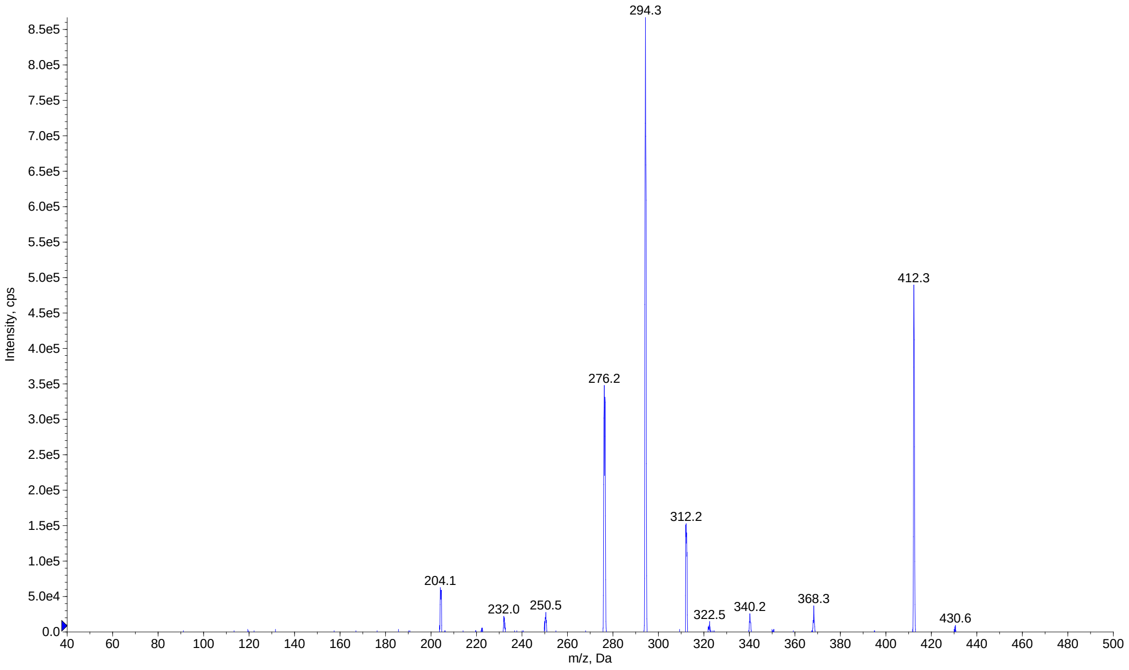

Figure S59. (+)430 MS/MS spectrum of Oxa-Thr-PA (mono-Ph$(CH_2)_2$-amide derivative #2)

EDC-mediated coupling of Oxa-Thr-PA with 2-phenylethanamine (continued)

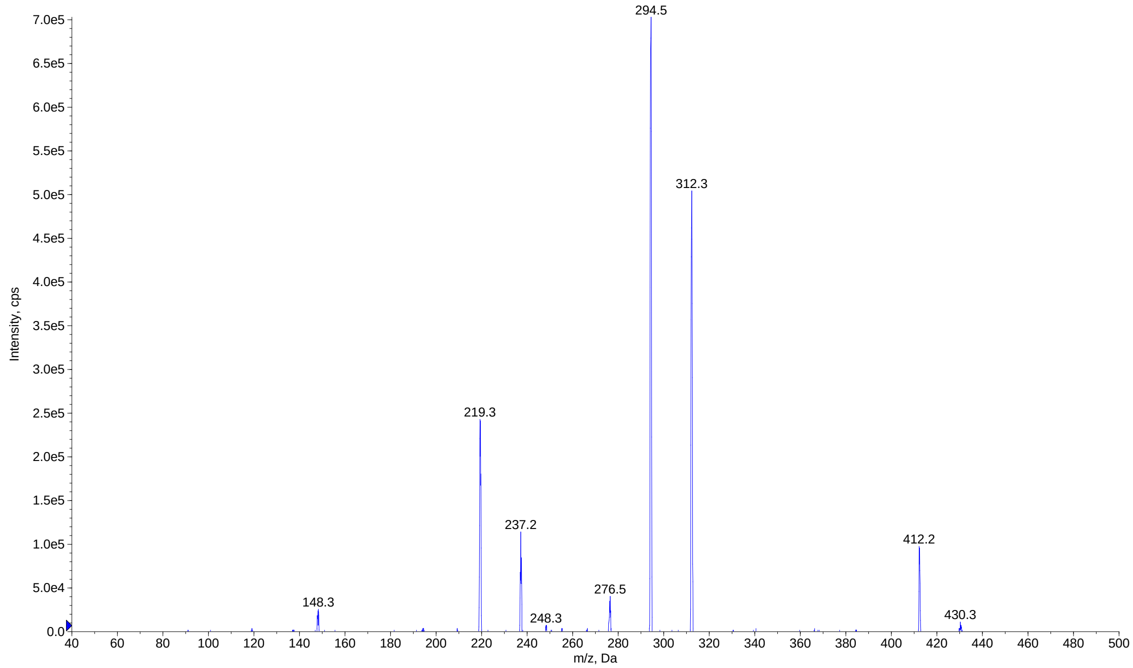

Figure S60. (+)430 MS/MS spectrum of Oxa-Thr-PA (mono-Ph(CH_2)$_2$-amide derivative #3)

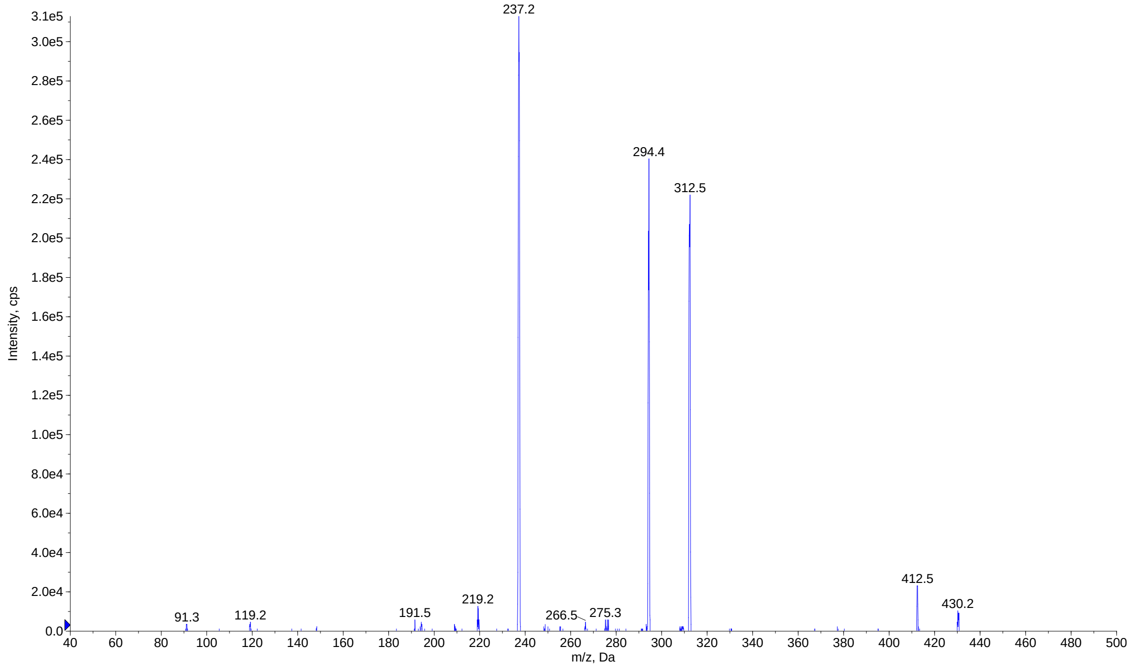

Figure S61. (+)430 MS/MS spectrum of Oxa-Thr-PA (mono-Ph(CH_2)$_2$-amide derivative #4)

EDC-mediated coupling of Oxa-Thr-PA with 2-phenylethanamine (continued)

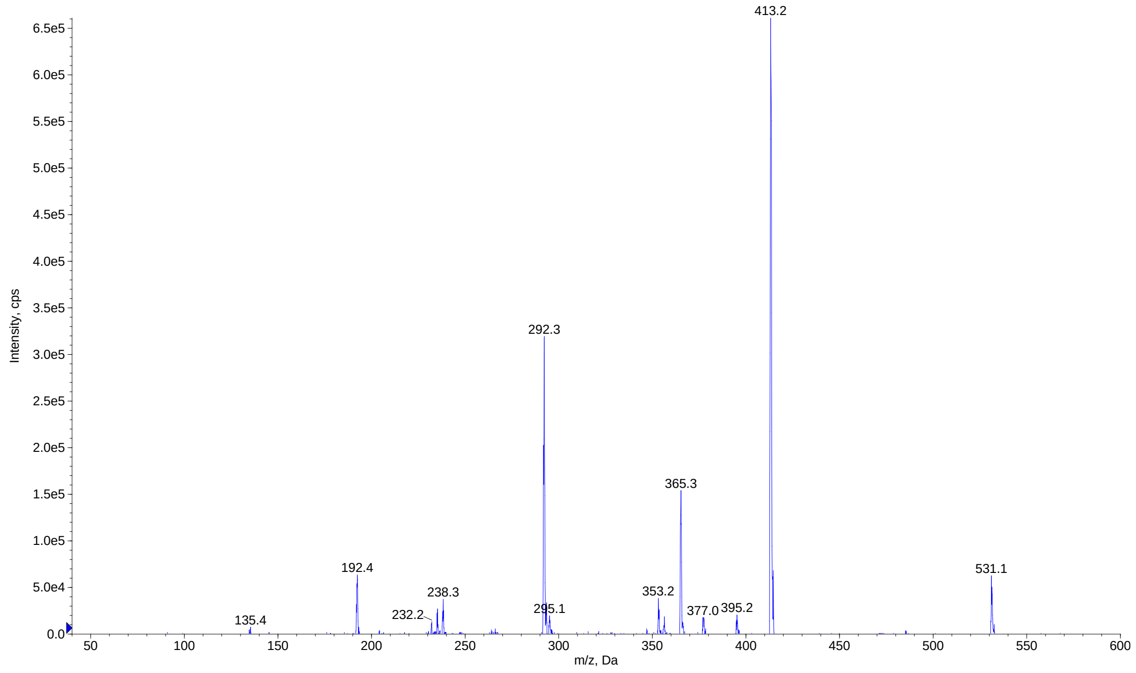

Figure S62. (-)531 MS/MS spectrum of Oxa-Thr-PA (bis-Ph$(CH_2)_2$-amide derivative)

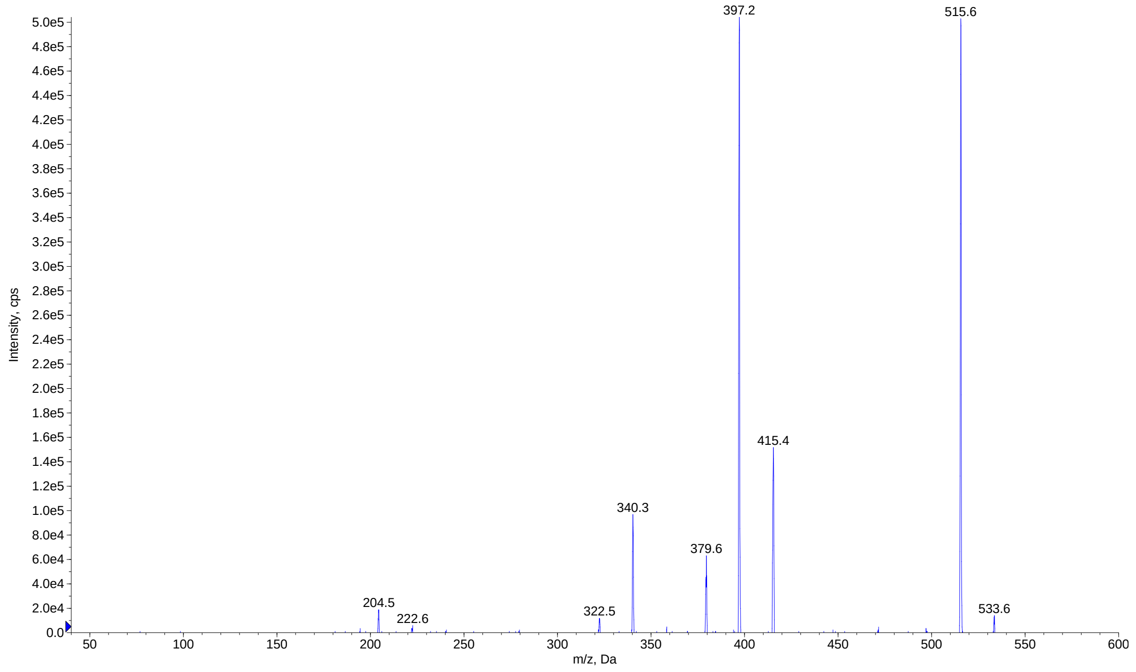

Figure S63. (+)533 MS/MS spectrum of Oxa-Thr-PA (bis-Ph$(CH_2)_2$-amide derivative)

EDC-mediated coupling of Oxa-Thr-PA with 2-phenylethanamine (continued)

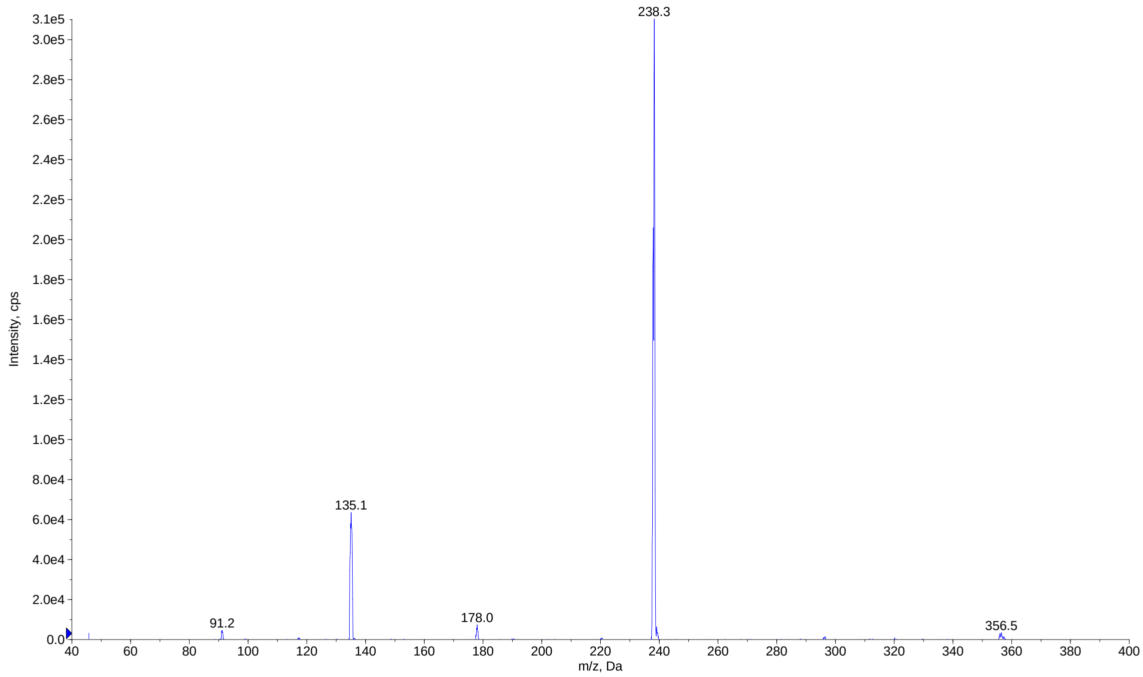

Figure S64. (-)356 MS/MS spectrum of Thr-PA ($Ph(CH_2)_2$-amide derivative)

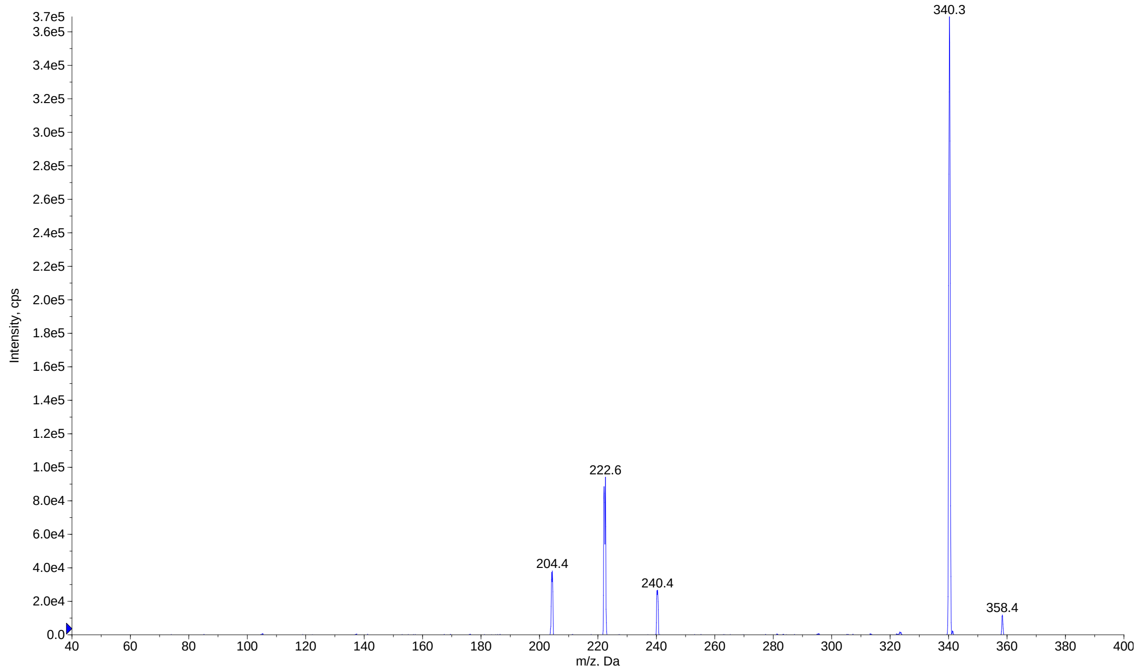

Figure S65. (+)358 MS/MS spectrum of Thr-PA ($Ph(CH_2)_2$-amide derivative)

EDC-mediated coupling of Oxa-Thr-PA with 2-phenylethanamine (continued)

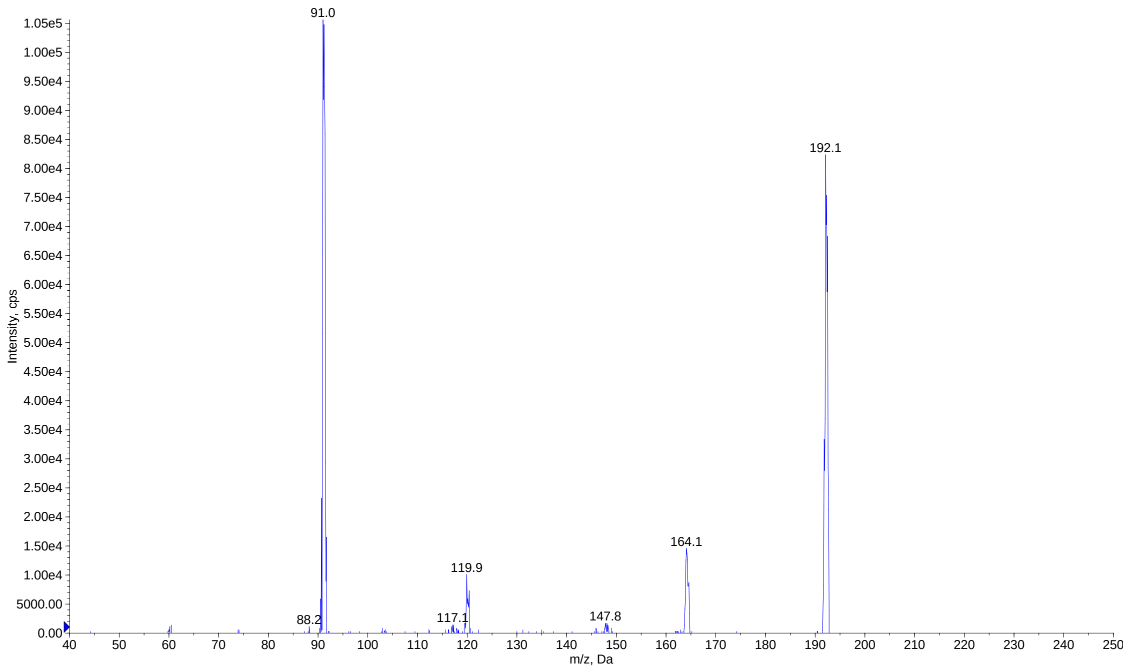

Figure S66. (-)192 MS/MS spectrum of oxalate (mono-Ph$(CH_2)_2$-amide derivative)

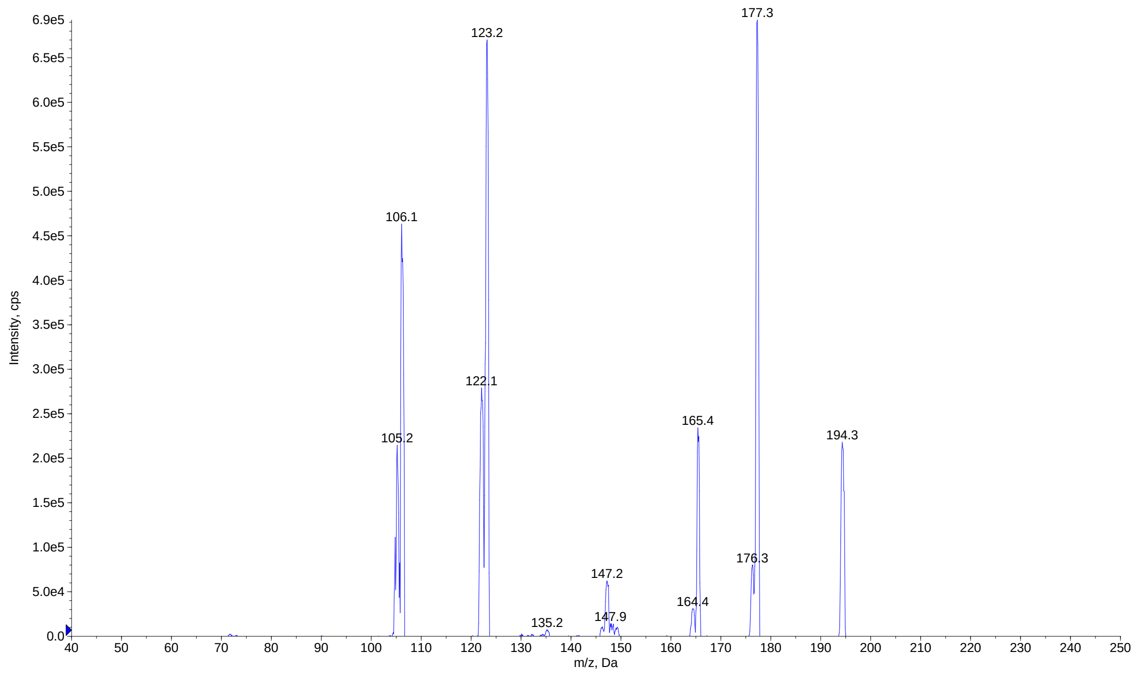

Figure S67. (+)194 MS/MS spectrum of oxalate (mono-Ph$(CH_2)_2$-amide derivative)

Oxidation of Asc-PA by peroxyl radicals derived from azo compounds

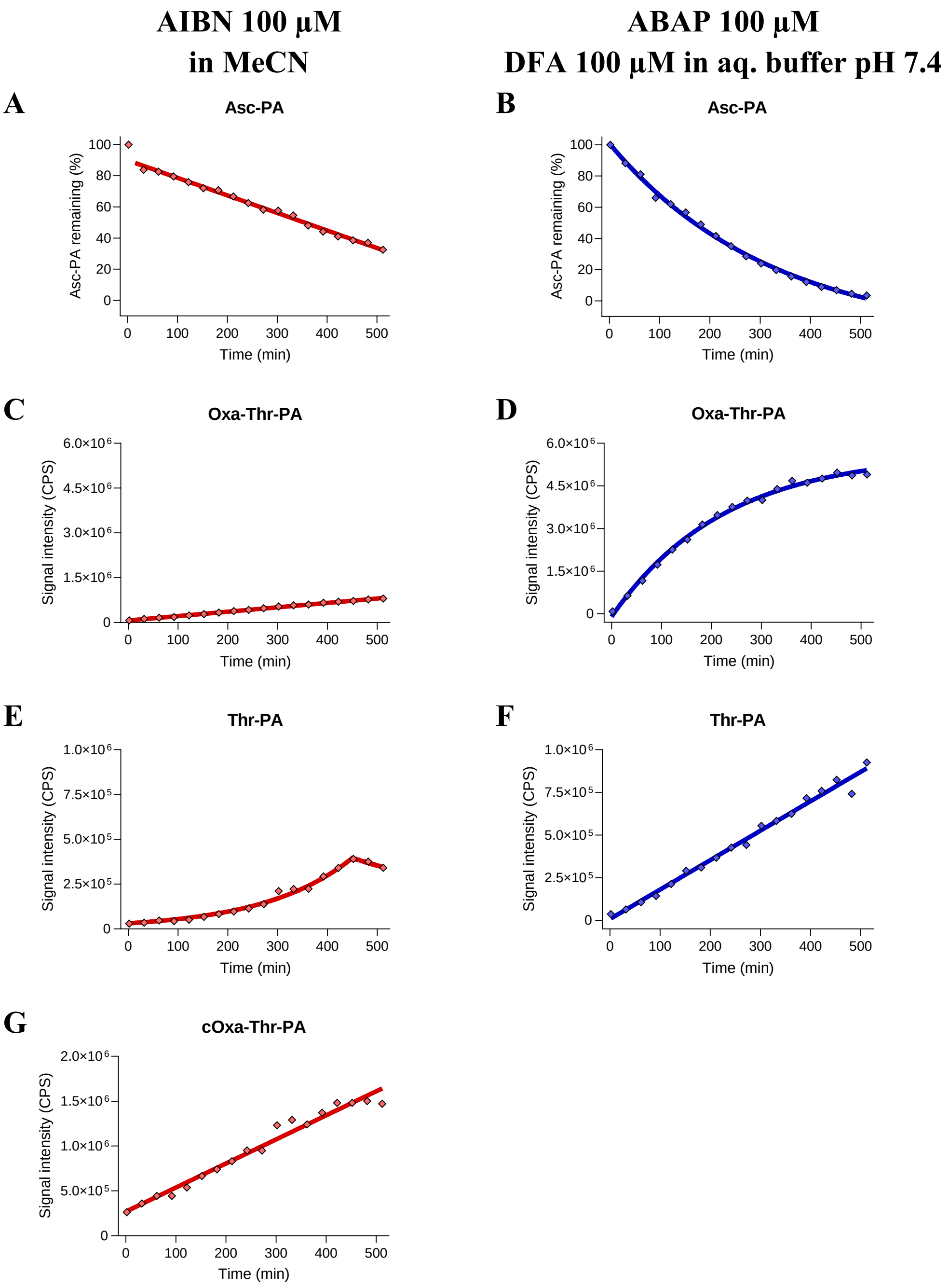

Figure S68. Formation of oxidized derivatives of Asc-PA by reaction with peroxyl radicals derived from the thermal decomposition of azo compounds, i.e., AIBN (red curves; A, C, E, G) and ABAP (blue curves; B, D, F), cOxa-Thr-PA not detected in aqueous solution
